# Genetic Characterization, Pathogenicity, and Epidemiology Analysis of Three Sub-Genotype Pigeon Newcastle Disease Virus Strains in China

**DOI:** 10.3390/microorganisms12040738

**Published:** 2024-04-04

**Authors:** Zeren Wang, Zhengyang Geng, Hongbo Zhou, Pengju Chen, Jing Qian, Aizhen Guo

**Affiliations:** 1 State Key Laboratory of Agricultural Microbiology, College of Veterinary Medicine, Huazhong Agricultural University, Wuhan 430070, China; wangzeren008@163.com (Z.W.);; 2 Zhoukou Animal and Plant Disease Prevention and Control Center, Zhoukou 466000, China; 3 Key Laboratory of Veterinary Biological Engineering and Technology, Institute of Veterinary Medicine, Jiangsu Academy of Agricultural Sciences, Nanjing 210014, China; 4 Henan Institute of Modern Chinese Veterinary Medicine, Zhengzhou 450002, China

**Keywords:** pigeon Newcastle disease virus, sub-genotype VI.2.1.1.2.2, genetic characterization, pathogenicity, molecular epidemiology

## Abstract

Pigeon Newcastle disease (ND) is a serious infectious illness caused by the pigeon Newcastle disease virus (NDV) or Paramyxovirus type 1 (PPMV-1). Genotype VI NDV is a primary factor in ND among Columbiformes (such as pigeons and doves). In a recent study, eight pigeon NDV strains were discovered in various provinces in China. These viruses exhibited mesogenic characteristics based on their MDT and ICPI values. The complete genome sequences of these eight strains showed a 90.40% to 99.19% identity match with reference strains of genotype VI, and a 77.86% to 80.45% identity match with the genotype II vaccine strain. Additionally, analysis of the F gene sequence revealed that these NDV strains were closely associated with sub-genotypes VI.2.2.2, VI.2.1.1.2.1, and VI.2.1.1.2.2. The amino acid sequence at the cleavage site of the F protein indicated virulent characteristics, with the sequences ^112^KRQKRF^117^ and ^112^RRQKRF^117^ observed. Pigeons infected with these sub-genotype strains had a low survival rate of only 20% to 30%, along with lesions in multiple tissues, highlighting the strong spread and high pathogenicity of these pigeon NDV strains. Molecular epidemiology data from the GenBank database revealed that sub-genotype VI.2.1.1.2.2 strains have been prevalent since 2011. In summary, the findings demonstrate that the prevalence of genotype VI NDV is due to strains from diverse sub-genotypes, with the sub-genotype VI.2.1.1.2.2 strain emerging as the current epidemic strain, highlighting the significance of monitoring pigeon NDV in China.

## 1. Introduction

Newcastle disease (ND) is a highly contagious viral infection that can be particularly devastating in poultry that have not been previously exposed to the virus [1]. The disease is caused by virulent strains of the Newcastle disease virus (NDV), also known as Avian Paramyxoviruses 1 (APMV-1), which belong to the *Avian orthoavulavirus* 1 (AOAV-1) genus in the *Paramyxoviridae* family [2]. The NDV genome is composed of a single-strand, negative-sense RNA with six structural proteins (NP, P, M, F, HN, L) and two non-structural proteins (V and W) produced via RNA editing from the P gene.

Genetic analysis of complete F gene nucleotide sequences is used to classify and name NDV isolates [3]. NDV strains are divided into two classes (I and II), with class I containing only one genotype (genotype 1) and class II including at least twenty genotypes (I–XXI, excluding XV) [3]. Class I viruses are non-virulent, while several class II strains (genotype II, III, VI, VII, IX) are virulent and can cause significant economic losses in the poultry industry [4,5]. Genotype VI NDV can be further divided into seven sub-genotypes (VI.1, VI.2.1.1.1, VI.2.1.1.2.1, VI.2.1.1.2.2, VI.2.1.2, VI.2.2.1, and VI.2.2.2) [3].

Genotype VI NDV, also known as pigeon paramyxovirus type-1 (PPMV-1), is a significant cause of Newcastle disease in pigeons and doves [6]. This strain was first discovered in captive pigeons in the Middle East in 1978 and has since been found in outbreaks among pet and racing pigeons in Europe and North America [7]. The virus is primarily transmitted between pigeons in squab facilities and private lofts, with occasional contamination of feed leading to transmission to domestic poultry [5,8]. Recent studies have shown that genotype VI NDV strains are circulating in several provinces in China [5,9]. Pigeons infected with this strain exhibit clinical signs similar to neurotropic ND-infected chickens, with moderate morbidity and low mortality rates. Currently, in China, there is a lack of commercialized genotype VI strains for PPMV-1 immunization. Pigeons are usually vaccinated with chicken-derived ND vaccines such as the LaSota or Clone 30 strains, which belong to the genotype II strains. Studies have revealed antigenic distinctions between genotype VI and genotype II strains [10,11,12], implying that pigeons immunized with genotype II and genotype IV vaccines may not have complete protection against genotype VI strains. The importance of the continuous surveillance of genotype VI NDV in pigeons has been emphasized in order to accurately monitor its spread and to develop effective control strategies [8,12,13,14,15,16,17,18].

This study conducted molecular and phylogenetic analyses of pigeon NDV isolates in China, evaluated their virulence, analyzed key protein characteristics, and assessed the pathogenicity of different sub-genotypes on pigeons. The results gathered in the current study could provide valuable insights for the molecular epidemiological investigation of pigeon NDV in China.

## 2. Materials and Methods

### 2.1. Virus Isolation and Virulence Tests

A total of 8 samples were collected from pigeons showing symptoms of Newcastle disease (ND) in Jiangsu (specifically Nanjing city, 1 sample in 2006), Zhejiang (Hangzhou and Wenzhou cities, 3 samples in 2017 and 2022), and Hebei (Tangshan city, 4 samples in 2023) provinces. The strains were isolated by inoculating specific pathogen-free (SPF) chicken eggs at 9 days old (Nanjing Tech-bank Bio-industry Co., Ltd, Nanjing, China. Voucher number: SCXK(su)2021-0005) [19]. The allantoic fluids were then tested for the presence of NDV using haemagglutination (HA) and haemagglutination-inhibition (HI) assays with NDV-specific antiserum and avian influenza virus (AIV) H5, H7, and H9-subtype sera (Harbin Weike Biotechnology Development Company, Harbin, China) [19]. The isolates were purified through plaque purification on primary chicken embryo fibroblasts (CEF) and amplified in 9-day-old SPF chicken embryos. The virus titer was calculated as the 50% embryo infectious dose (EID_50_/mL) using the Reed and Muench end-point method. The virus-containing allantoic fluids were stored at −80 °C until further use. ICPI tests were conducted on 1-day-old SPF chickens (Nanjing Tech-bank Bio-industry Co., Ltd, Nanjing, China. Voucher number: SCXK(su)2021-0005) and mean death time (MDT) tests were performed on 9-day-old SPF chicken eggs following the guidelines of the Office International des Epizooties manual of standards [19].

### 2.2. Viral Genome Sequencing

The viral genomic RNA was isolated from the harvested allantoic fluids using a SteadyPure Virus DNA/RNA Extraction Kit from Accurate Biotechnology (Hunan) Co., Ltd., Changsha, China, following the manufacturer’s instructions. The RNA pellets were then dissolved in 50 μL of RNase-free water and reverse transcribed using the Evo M-MLV Plus 1st Strand cDNA Synthesis Kit from Accurate Biotechnology (Hunan) Co., Ltd., Changsha, China. The resulting cDNAs were utilized as templates to generate eleven successive and overlapping DNA fragments through PCR, with specific primer pairs for genotype VI NDV strains (refer to Table S1). To identify the 3′- and 5′-ends of the viral genomes, rapid amplification of cDNA end (RACE) was carried out using the SMARTer RACE 5′/3′ Kit from Takara Biomedical Technology Co., Ltd., Beijing, China, as per the manufacturer’s instructions.

The PCR products obtained were ligated into pMD™19-T vectors from Takara Biomedical Technology Co., Ltd., Beijing, China, and transformed into *Escherichia coli* DH5α competent cells from Takara Biomedical Technology Co., Ltd., Beijing, China. At least three clones for each segment were submitted to General Biosystems Co., Ltd. (Chuzhou, China) for sequencing.

### 2.3. Sequence and Phylogeny Analysis

The nucleotide sequences of the whole genome were aligned using ClustalW software (version 2.0.10) and compared with the corresponding sequences of 21 other NDV strains from different genotypes. These alignments were then analyzed for both nucleotide and deduced amino acid sequences. MEGA software (version 7) was employed for whole genome nucleotide identity analysis and for the analysis of key amino acid site mutations in the F and HN proteins.

Phylogenetic analysis was conducted on the complete F gene sequences (1662 nt), along with 43 other strains from various genotypes or sub-genotypes using MEGA software (version 7) [20]. The evolutionary history was determined using the Maximum Likelihood method based on the General Time Reversible (GTR) model, with a discrete gamma distribution (+G), allowing for invariant sites and 500 bootstrap replicates [21]. The tree was constructed to scale, with branch lengths measured in the number of substitutions per site. The data and accession numbers of the NDV reference strains used in the study can be found in Table S2.

### 2.4. Clinicopathologic Assessment in Pigeons

A total of 40 healthy 30-day-old pigeons (Voucher number: SCXK(su)2021-0012) were randomly divided into four groups for this study, with each group consisting of 10 pigeons. These pigeons did not have Newcastle Disease Virus (NDV)-specific HI antibodies and tested negative for the NDV genome in cloacal swab samples. The pigeons were then inoculated with three sub-genotype strains (VI.2.1.1.2.2 strain corresponds to Pigeon/China/WZ2205/2022, while VI.2.1.1.2.1 strain corresponds to Pigeon/China/VI-NJ/2006, and VI.2.2.2 strain corresponds to Pigeon/China/VI-HZ/2017) of NDV via the intranasal route with 10^6^ EID_50_ of each virus in 0.2 mL of saline. A negative control group was given 0.2 mL of sterile saline. Every pigeon received its inoculation at the same hour of the day. The pigeons in the experimental groups were housed in a negative-pressure environment and had access to food and water at all times. The pigeons were monitored daily for signs of illness and mortality. On day 14 post-inoculation, pigeons from each group were sacrificed, and tissue samples (brain, trachea, and intestine) were collected and fixed in 10% neutral buffered formalin for histological examination. The tissue sections were then stained with hematoxylin and observed under a light microscope.

Every pigeon in every group had cloacal swabs collected and underwent further testing during the experiment. The cloacal swab samples collected at 3, 5, 7, 10, and 14 days post-infection were collected at the same hour each day post-infection and the supernatants were introduced into 9-day-old SPF chicken eggs to track virus shedding. After 72 h, the allantoic fluid was retrieved and the virus was quantified using a standard HA assay [19]. Samples exhibiting HA values equal to or greater than 2 log_2_ were marked as positive.

### 2.5. Molecular Epidemiology of Pigeon NDV from GenBank Database

In order to gain a better understanding of the global and Chinese prevalence of pigeon NDV, a total of 1329 pigeon NDV strains from the GenBank database (accessed on 6 February 2024) were analyzed by screening their partial or complete F gene sequences. Each sequence was carefully assessed, with any gaps or insertions causing alignment shifts being removed. Information regarding the country of origin and collection date was also documented.

## 3. Results

### 3.1. Virus Isolation and Identification

The samples were inoculated into embryonated chicken eggs to isolate strains, and the HA activity of the isolated strains was detected in the allantoic fluid of the embryos. The HA values of each virus ranged from 6 log_2_ to 9 log_2_, and all viruses were able to react with serum positive for the NDV HI test, but not with AIV H5, H7, and H9-subtype sera. After multiple rounds of plaque purification, eight NDV strains were successfully isolated and identified as Pigeon/China/VI-NJ/2006, Pigeon/China/VI-HZ/2017, Pigeon/China/WZ2201/2022, Pigeon/China/WZ2205/2022, Pigeon/China/HB2306/2023, Pigeon/China/HB2307/2023, Pigeon/China/HB2308/2023, and Pigeon/China/HB2309/2023. The MDT and ICPI values of these strains were consistent with mesogenic NDV characteristics. More detailed information about the eight isolates can be found in Table 1.

### 3.2. Genomic and Phylogenetic Analysis

A total of 11 overlapping DNA segments ranging from 1200 bp to 1700 bp were obtained through RT-PCR, with the 3′ leader and 5′ trailer lengths being 55 nt and 114 nt, respectively. A sequence analysis of eight pigeon NDV strains, totaling 15,192 nt, was assembled using DNAMAN software (version 9.0). These strains exhibited the gene order 3′-NP-P-M-F-HN-L-5′, with the open reading frame (ORF) sequences of the six structural proteins (NP, P, M, F, HN, L) measuring 1470 bp, 1188 bp, 1095 bp, 1662 bp, 1716 bp, and 6615 bp, respectively.

**Figure 1:**
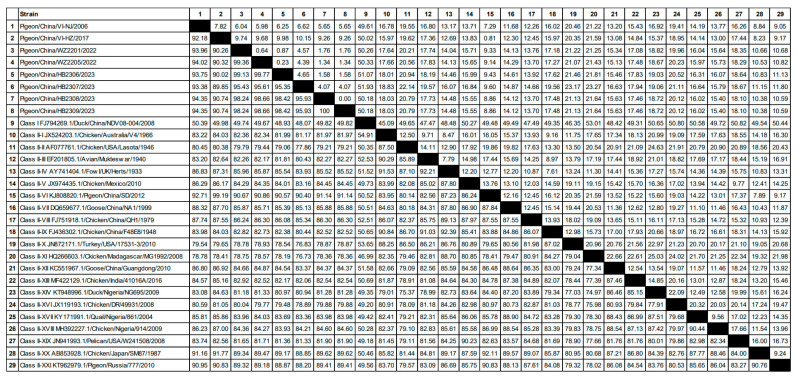
Nucleotide identity analysis was performed on the whole genome sequences of eight pigeon NDV isolates detected in this study, as well as 21 other reference NDV strains.

The analysis of the whole genome nucleotide identity of the 8 pigeon NDV strains compared to 21 reference strains of each genotype revealed distinct similarities, ranging from 48.07% to 99.19%. Among the reference strains, eight isolates were closely related to pigeon genotype VI strains, with identities ranging from 90.40% to 99.19%, and also showed similarities of 88.20% to 90.95% with another pigeon genotype XXI strain. These isolates had identities of 85.13% to 88.32% with the predominant genotype VII strains in China, and only 77.86% to 80.45% with genotype II vaccine strains. Furthermore, the whole genome nucleotide sequences of the eight isolates shared identities of 90.74% to 100% with each other (Figure 1).

**Figure 2:**
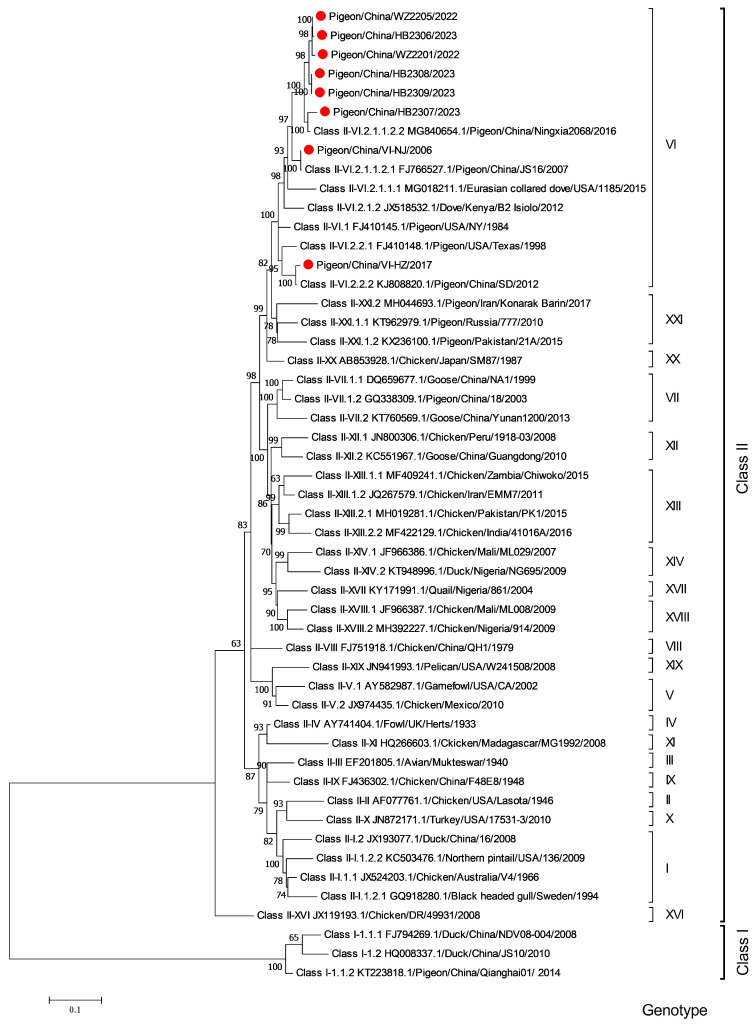
Phylogenetic analysis of the F region of eight pigeon NDV isolates detected in this study and other reference NDV strains (n = 43). A phylogenetic tree was constructed based on the complete F gene sequences using the maximum likelihood (ML) method with 500 bootstrap replicates and the Poisson model in MEGA 7.0 software. Note: Pigeon/China/VI-NJ/2006, Pigeon/China/VI-HZ/2017, Pigeon/China/WZ2201/2022, Pigeon/China/WZ2205/2022, Pigeon/China/HB2306/2023, Pigeon/China/HB2307/2023, Pigeon/China/HB2308/2023, and Pigeon/China/HB2309/2023 in this study are labeled with a red solid circle (●).

The phylogenetic tree based on the F gene revealed that eight isolates belonged to the Class II genotype VI group. The Pigeon/China/VI-NJ/2006 strain was closely related to sub-genotype VI.2.1.1.2.1, while the Pigeon/China/VI-HZ/2017 strain had the closest genetic relationship with sub-genotype VI.2.2.2. The remaining six isolates, including Pigeon/China/WZ2201/2022, Pigeon/China/WZ2205/2022, Pigeon/China/HB2306/2023, Pigeon/China/HB2307/2023, Pigeon/China/HB2308/2023, and Pigeon/China/HB2309/2023, were classified as sub-genotype VI.2.1.1.2.2 in the phylogenetic analysis (Figure 2).

The amino acid sequence at the cleavage site of the F protein of Pigeon/China/VI-HZ/2017 was ^112^KRQKRF^117^, while that of the other seven isolates was ^112^RRQKRF^117^ (Table 2), which is characteristic of virulent strains. Most pigeon NDV strains had six N-glycosylation sites (N85, N191, N366, N447, N471, and N541) in the F protein and five N-glycosylation sites (N119, N341, N433, N481, and N508) in the HN protein. Additionally, the Pigeon/China/VI-HZ/2017 strain had an additional glycosylation site (N497) in the F protein, similar to the VI.2.2.2 reference strain. However, this strain also had a mutated N508S site, similar to the VI.2.1.2 reference strain. The key amino acid sites of the F and HN proteins are shown in detail in Table 2.

### 3.3. Clinical Symptoms and Gross Pathology

Three sub-genotype VI.2.2.2 (Pigeon/China/VI-HZ/2017), VI.2.1.1.2.1 (Pigeon/China/VI-NJ/2006), and VI.2.1.1.2.2 (Pigeon/China/WZ2205/2022) strains were used to evaluate their pathogenicity in pigeons. Clinical signs were observed in pigeons infected with each of the three sub-genotype strains, starting at 2 days post-infection (dpi). The birds showed decreased appetite at 5 dpi, which became more severe as the disease progressed.

In the advanced stage of pigeon infection with three sub-genotype strains of NDV, noticeable neurological symptoms such as head tilting and tremors become apparent (Supplement Figure S1a–c). Visual changes included scattered small bleeding points in the pigeon’s brain (Supplement Figure S1e–g). Additionally, significant bleeding points were observed in the throat and trachea (Supplement Figure S1i,k,l). The muscular stomach showed bleeding spots, with scattered small bleeding points at the junction of the muscular stomach and the glandular stomach, along with bleeding and edema in the glandular gastric papilla (Supplement Figure S1m–o). Throughout the entire intestine, bleeding spots were present, accompanied by congestion and necrosis in the pancreas (Supplement Figure S1q–s). There were no clinical symptoms or gross lesions observed in the pigeons in the control group (Supplement Figure S1d,h,l,p,t). However, no significant differences were noted in clinical symptoms and gross lesions among pigeons infected with the three sub-genotype strains.

**Figure 3:**
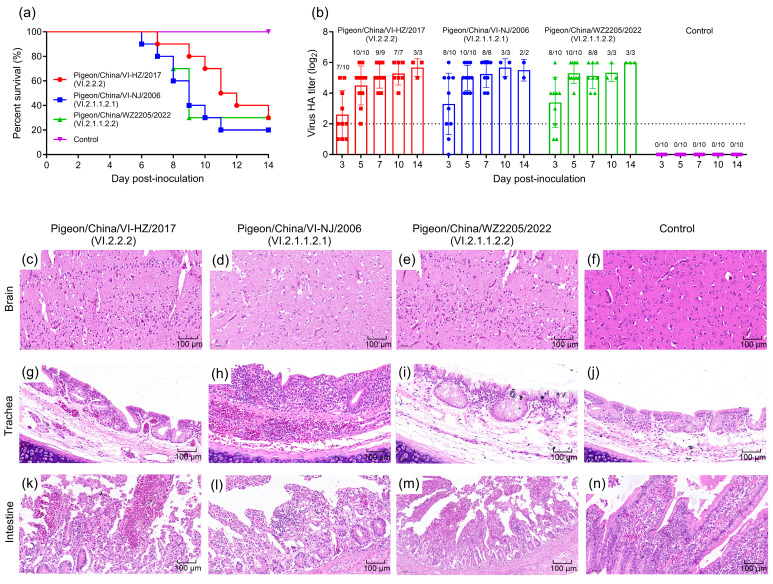
Survival rates, virus shedding, and histopathological observations of tissues were recorded from pigeons inoculated with three sub-genotypes of NDV: Pigeon/China/VI-NJ/2006, Pigeon/China/VI-HZ/2017, and Pigeon/China/WZ2205/2022. (**a**) Survival rate of pigeons inoculated with three sub-genotype strains. (**b**) Detection of virus shedding from pigeons with three sub-genotype strains on Day 14. (**c**–**e**) Edema in the brain. (**f**) No abnormality in the brain. (**g**–**i**) Necrosis and desquamation of mucous epithelial cells in the trachea. (**j**) No abnormality in the trachea. (**k**–**m**) Broken villi, dropout of epithelium, hemorrhage, and lamina propria inflammatory reaction in the intestine. (**n**) No abnormality in the intestine.

By 6 dpi, 80% (8/10) of pigeons infected with the Pigeon/China/VI-NJ/2006 strain had died. The mortality rate for pigeons infected with the Pigeon/China/VI-HZ/2017 or Pigeon/China/WZ2205/2022 strains was 70% (7/10) for both. The survival rate for pigeons infected with any of the three sub-genotype strains was 20% to 30% (Figure 3a).

As shown in Figure 3b, the cloacal swabs of three sub-genotype strain-infected pigeons were positive at 3 dpi (Pigeon/China/VI-HZ/2017 Group was 7/10, whereas Pigeon/China/VI-NJ/2006 Group and Pigeon/China/WZ2205/2022 Group were both 8/10). The highest viral load values for Pigeon/China/VI-HZ/2017, Pigeon/China/VI-NJ/2006, and Pigeon/China/WZ2205/2022 were all on day 5 (10/10). The samples from the control group were all negative for NDV throughout the experiment.

Histopathological analysis of pigeons infected with three different strains revealed brain edema in all cases (Figure 3c–e). Additionally, necrosis and desquamation of mucous epithelial cells were observed in the tracheas of infected pigeons (Figure 3g–i). The small intestine showed broken villi, epithelial dropout, and hemorrhage, along with inflammatory reactions in the lamina propria (Figure 3k–m). In contrast, tissue samples from the control group showed no significant histological changes (Figure 3f,j,n). Overall, the three sub-genotype strains exhibited neurotropism and invasiveness in the gastrointestinal tract, demonstrating strong spreading ability and high pathogenicity.

### 3.4. Prevalence of Pigeon NDV Strains in China

**Figure 4:**
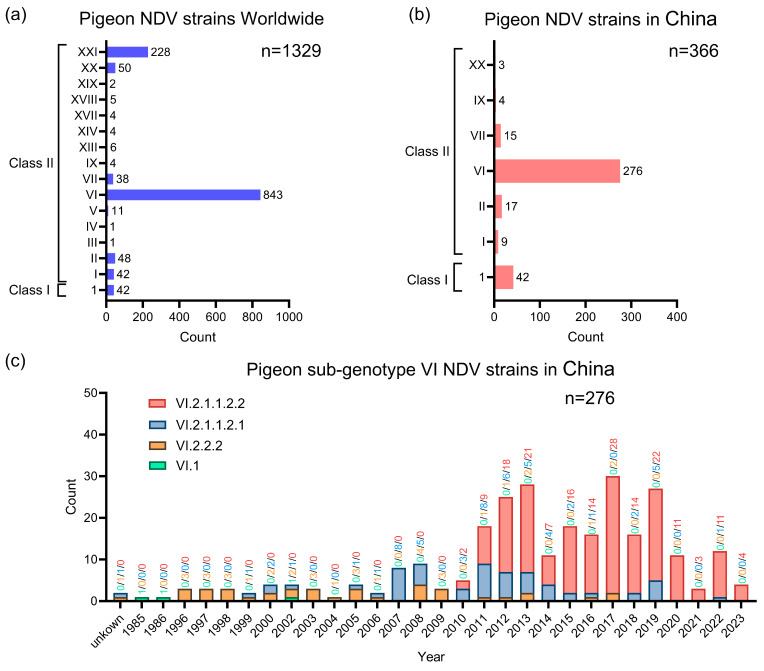
Investigating Pigeon Newcastle Disease Virus using Molecular Epidemiology. (**a**) Worldwide genotypic classification and statistics of pigeon Newcastle disease virus are essential for understanding the spread of the virus. (**b**) Genotypic classification and statistics of pigeon Newcastle disease virus in China are as follows. (**c**) Statistics on the isolation time of Chinese genotype 6 pigeon Newcastle disease virus. The numbers and colors on each column represent the number of different sub-genotype strains, with green representing genotype VI.1, orange representing genotype VI.2.2.2, blue representing genotype VI.2.1.1.2.1, and red representing genotype VI.2.1.1.2.2.

Based on the analysis of 1329 strains available in GenBank, pigeon NDV has appeared in Europe, Asia, Africa, America, and Australia (Supplement Table S3). As shown in Figure 4a, the genotypes of global pigeon NDV can be divided into 16 genotypes (genotype 1 in the Class I clade, genotypes I, II, III, IV, V, VI, VII, IX, XIII, XIV, XVII, XVIII, XIX, XX, and XXI in the Class II clade), with genotype VI (n = 843) and XXI (n = 228) being the most prevalent. In addition to the 8 isolates in this study, another 358 pigeon strains from China were identified as 9 genotype I (2.46%), 17 genotype II (4.64%), 276 genotype VI (75.41%), 15 genotype VII (4.10%), 4 genotype IX (1.09%), and 3 genotype XX (0.82%) in the Class II clade, and there were also 42 genotype 1 strains in the Class I clade (11.48%; Figure 4b). Furthermore, 276 pigeon sub-genotype VI strains, consisting of sub-genotypes VI.1, VI.2.1.1.2.1, VI.2.1.1.2.2, and VI.2.2.2, were found between 1985 and 2023 (Supplement Table S3). As shown in Figure 4c, the sub-genotype VI.2.1.1.2.2 strain first appeared in 2010 and is the most prevalent among the three sub-genotype strains. The annual frequency analysis of these sub-genotype strains suggests that the VI.2.1.1.2.2 strain has been prevalent since 2011 and has become dominant among pigeon NDV strains in China.

## 4. Discussion

Pigeon NDV or PPMV-1 is a highly pathogenic and severe infectious disease. Both young and adult pigeons can be infected, which causes significant economic losses in the pigeon industry [5,18]. Although some strains of PPMV-1 are non-pathogenic in chickens, they can cause morbidity in pigeons. The disease signs in pigeons are consistent with those in chickens and generally include a series of nervous disorders: bilateral or unilateral locomotor disturbances of the wings or legs, torticollis, and watery green diarrhea. If pigeons are infected during breeding or molting, the mortality risk tends to increase. This disease is highly contagious and can spread rapidly through a flock of pigeons. It is important for pigeon owners to practice strict biosecurity measures to prevent the spread of the virus. Additionally, there is currently no specific treatment for Pigeon ND.

Most pigeon-derived virulent isolates belong to class II of NDV. NDV strains of several genotypes have been isolated and detected in pigeons, including genotypes VI, VII, and XXI [16,22,23]. In China, the strains that infect pigeons are mainly of genotype VI [12,16,17,18]. The two strains also belong to genotype VI, suggesting that genotype VI is the dominant strain in pigeons in China. Genotype VI strains can infect many kinds of birds, including pigeons, chickens, turkeys, quails, and geese [5]. However, they do not cause obvious symptoms in other species [24]. There are still potential hazards to the aquaculture industry; therefore, it is very important to implement epidemiological testing for PPMV-1.

The classification of NDV genotypes was updated by Dimitrov et al. [3]. Former sub-genotypes VIc, VIl, VIi, VIg, and VIm were confirmed and renamed as genotypes XX and XXI [3]. Therefore, the two strains showed a close genetic distance from these three genotypes (VI, XX, and XXI). Moreover, genotype VI strains can be further divided into at least seven sub-genotypes (VI.1, VI.2.1.1.1, VI.2.1.1.2.1, VI.2.1.1.2.2, VI.2.1.2, VI.2.2.1, and VI.2.2.2) [3]. Currently, the most prevalent genotypes of PPMV-1 in other countries are VI.1, VI.2.1.1.1, VI.2.1.2, and VI.2.2.1. In China, the main genotypes are VI.2.2.2, VI.2.1.1.2.1, and VI.2.1.1.2.2 [3]. Tian et al. found that ten PPMV-1 viruses isolated in China during 1996–2019 belonged to sub-genotypes VI.2.2.2, VI.2.1.1.2.1, and VI.2.1.1.2.2 and genotype VII [16]. Additionally, Zhan et al. reported that 21 PPMV-1 isolates belonged to sub-genotypes VI.2.1.1.2.1 and VI.2.1.1.2.2 in China from 2007 to 2019 [17]. In this study, we identified eight strains of PPMV-1 from several provinces in China during 2006–2023; these were classified into sub-genotypes VI.2.2.2, VI.2.1.1.2.1, and VI.2.1.1.2.2. These results indicate that the prevalence of PPMV-1 is the result of different genotype strains circulating in China.

Furthermore, most pigeon NDV strains have a consistent number of glycosylation sites in their F and HN proteins, which are glycoproteins involved in the adsorption, binding, and membrane fusion of viral particles. Compared to other genotypes, sub-genotype VI.2.2.2 strains have a unique glycosylation site at position 497. Interestingly, we also observed a mutation (N508S) in the HN protein of the Pigeon/China/VI-HZ/2017 strain. This sub-genotype is rarely seen, so it is possible that this mutation was an accidental event. This mutation may have implications for the pathogenicity and antigenicity of the virus, and further research is needed to understand its significance.

The molecular epidemiology of the GenBank database has shown that four sub-genotype strains (VI.1, VI.2.2.2, VI.2.1.1.2.1, and VI.2.1.1.2.2) have appeared in China. The occurrence rate of sub-genotype VI.2.1.1.2.2 strains has been dominant among pigeon NDV strains since 2011 in China. To date, no genotype XXI or sub-genotypes VI.2.1.1.1, VI.2.1.2, and VI.2.2.1 strains have been monitored in China. However, it is still necessary to strengthen the long-term surveillance of NDV. This is important in order to closely monitor the potential emergence of new sub-genotypes or genotypes in China and to better understand the molecular evolution and epidemiology of NDV in the country.

## 5. Conclusions

In summary, the complete genomes of eight genotype VI viruses obtained from pigeons in China were identified and examined using a recently established phylogenetic classification system. The strains belonging to sub-genotypes VI.2.2.2, VI.2.1.1.2.1, and VI.2.1.1.2.2 exhibited high virulence and shedding capabilities in pigeons. The findings indicated that the prevalence of genotype VI NDV was influenced by strains from various sub-genotypes. While these isolates were only detected in certain provinces of China, the molecular epidemiological data suggests that the sub-genotype VI.2.1.1.2.2 NDV strain has been predominant in the country. It is crucial to understand the spread and impact of genotype VI NDV in China in order to develop effective prevention and control strategies. This could represent a starting point for future development of a pigeon ND vaccine.

## Figures and Tables

**Table 1:** Eight pigeon NDV isolates characterized in this study.

No.	Strain	MDT ^a^ (h)	ICPI ^b^	HA Titer	TCID_50_/0.1 mL	EID_50_/0.1 mL	GenBank Accession No.
1	Pigeon/China/VI-NJ/2006	68	1.71	8 log_2_	10^8.67^	10^8.67^	MZ409510.1
2	Pigeon/China/VI-HZ/2017	84	1.58	7 log_2_	10^7.33^	10^7.67^	MW412840.1
3	Pigeon/China/WZ2201/2022	90	1.20	7 log_2_	10^8.00^	10^7.67^	OP751936.1
4	Pigeon/China/WZ2205/2022	74	1.44	9 log_2_	10^8.50^	10^8.00^	OP796698.1
5	Pigeon/China/HB2306/2023	80	1.25	7 log_2_	10^7.67^	10^7.33^	OR860421
6	Pigeon/China/HB2307/2023	105	1.23	6 log_2_	10^7.50^	10^7.33^	OR860422
7	Pigeon/China/HB2308/2023	90	1.38	8 log_2_	10^7.67^	10^7.33^	OR860423
8	Pigeon/China/HB2309/2023	86	1.36	7 log_2_	10^7.67^	10^7.33^	OR860424

^a^ The mean death time (MDT) was determined by inoculating 9-day-old SPF chicken eggs for hours (<60 h for velogenic/highly virulent strains; 60–90 h for mesogenic/moderately virulent strains; >90 h for lentogenic/low virulent strains). ^b^ Values equal to 0.7 or greater are identified as virulent NDV strain.

**Table 2:** Summary table of the key amino acid sites in the F and HN proteins of eight pigeon NDV isolates detected in this study, compared with ten reference pigeon genotype VI and XXI NDV strains. In the F cleavage site, the down arrow (↓) denotes the exact spot where the protease recognizes and cleaves.

No.	Strain	Sub-Genotype	F Cleavage Site	N-Glycosylation Sites in F Protein							N-Glycosylation Sites in HN Protein				
				85	191	366	447	471	497	541	119	341	433	481	508
1	Pigeon/China/VI-NJ/2006	VI.2.1.1.2.1	^112^RRQKR↓F^117^	N	N	N	N	N	S	N	N	N	N	N	N
2	Pigeon/China/VI-HZ/2017	VI.2.2.2	^112^KRQKR↓F^117^	N	N	N	N	N	N	N	N	N	N	N	S
3	Pigeon/China/WZ2201/2022	VI.2.1.1.2.2	^112^RRQKR↓F^117^	N	N	N	N	N	S	N	N	N	N	N	N
4	Pigeon/China/WZ2205/2022	VI.2.1.1.2.2	^112^RRQKR↓F^117^	N	N	N	N	N	S	N	N	N	N	N	N
5	Pigeon/China/HB2306/2023	VI.2.1.1.2.2	^112^RRQKR↓F^117^	N	N	N	N	N	S	N	N	N	N	N	N
6	Pigeon/China/HB2307/2023	VI.2.1.1.2.2	^112^RRQKR↓F^117^	N	N	N	N	N	S	N	N	N	N	N	N
7	Pigeon/China/HB2308/2023	VI.2.1.1.2.2	^112^RRQKR↓F^117^	N	N	N	N	N	S	N	N	N	N	N	N
8	Pigeon/China/HB2309/2023	VI.2.1.1.2.2	^112^RRQKR↓F^117^	N	N	N	N	N	S	N	N	N	N	N	N
9	Pigeon/USA/NY/1984	VI.1	^112^RRQKR↓F^117^	N	N	N	N	N	S	N	N	N	N	N	N
10	Eurasian collared dove/USA/1185/2015	VI.2.1.1.1	^112^RRKKR↓F^117^	N	N	N	N	N	S	N	N	N	N	N	Y
11	Pigeon/China/JS16/2007	VI.2.1.1.2.1	^112^RRRKR↓F^117^	N	N	N	N	N	S	N	N	N	N	N	N
12	Pigeon/China/Ningxia2068/2016	VI.2.1.1.2.2	^112^RRQKR↓F^117^	N	N	N	N	N	S	N	N	N	N	N	N
13	Dove/Kenya/B2 Isiolo/2012	VI.2.1.2	^112^RRQKR↓F^117^	N	N	N	N	N	S	N	N	N	N	N	S
14	Pigeon/USA/Texas/1998	VI.2.2.1	^112^RRQKR↓F^117^	N	N	N	N	N	S	N	N	N	N	N	N
15	Pigeon/China/SD/2012	VI.2.2.2	^112^RRQKR↓F^117^	N	N	N	N	N	N	N	N	N	N	N	N
16	Pigeon/Russia/777/2010	XXI.1.1	^112^RRQKR↓F^117^	N	N	N	N	N	S	N	N	N	N	N	N
17	Pigeon/Pakistan/21A/2015	XXI.1.2	^112^RRQRR↓F^117^	N	N	N	N	N	S	N	N	N	N	N	N
18	Pigeon/Iran/Konarak Barin/2017	XXI.2	^112^RRQKR↓F^117^	N	N	N	N	N	S	N	N	N	N	N	N

## Data Availability

The NDV strain data obtained in this study is available from NCBI (https://www.ncbi.nlm.nih.gov/ (accessed on 6 February 2024), and others are presented in this study and its Supplementary Materials. The source code is available from the corresponding author upon request.

## Supplementary Materials

The following supporting information can be downloaded at: https://www.mdpi.com/article/10.3390/microorganisms12040738/s1, Table S1. Primers for sequencing the viral whole genome of the NDV in pigeons; Table S2. Basic information and genotypes of 43 NDV reference strains; Table S3. Basic information of 1329 pigeon NDV strains from GenBank database; Figure S1. Clinical symptoms and gross lesions of diseased pigeons inoculated with three sub-genotypes of NDV: Pigeon/China/VI-NJ/2006, Pi-geon/China/VI-HZ/2017, and Pigeon/China/WZ2205/2022.
