# Supplementary material for: Genetic Characterization, Pathogenicity, and Epidemiology Analysis of Three Sub-Genotype Pigeon Newcastle Disease Virus Strains in China
Source: Microorganisms. 2024 Apr 4;12(4):738. doi: 10.3390/microorganisms12040738 (PMC11051795; doi:10.3390/microorganisms12040738)
Supplement: Supplementary file 1 [file microorganisms-12-00738-s001.zip › Supplement Fig1 Clinical symptoms and gross lesions.pdf]

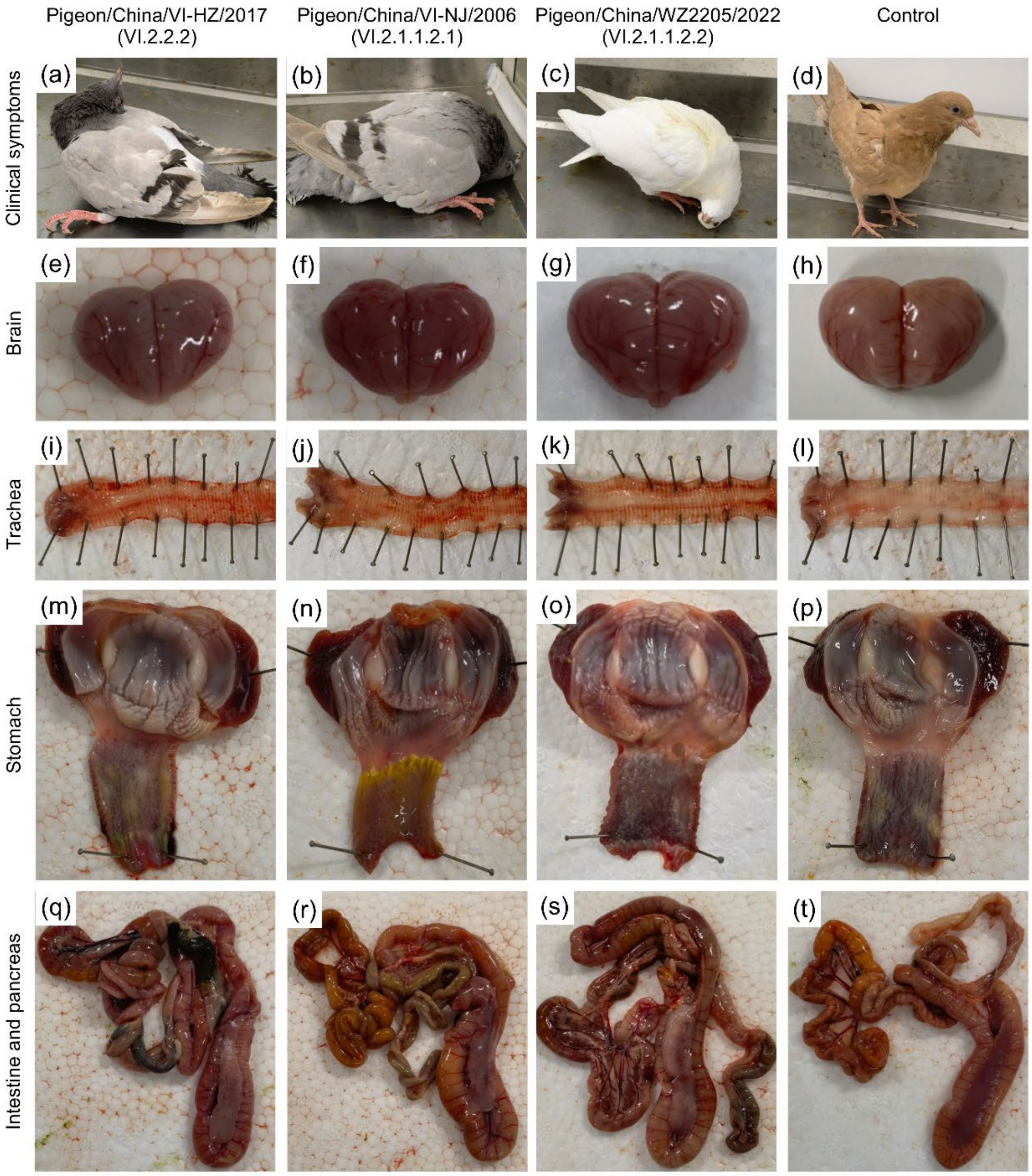

Supplement Figure S1. Clinical symptoms and gross lesions of diseased pigeons inoculated with three sub-genotypes of NDV: Pigeon/China/VI-NJ/2006, Pi-geon/China/VI-HZ/2017, and Pigeon/China/WZ2205/2022.

(a), (b) and (c) Neurological symptoms such as head tilting and tremors become apparent. (d) No neurological symptoms. (e), (f) and (g) The brain has scattered bleeding points. (h) No abnormality in the brain. (i), (j) and (k) The throat and trachea both have noticeable bleeding points. (l) No abnormality in the throat and trachea. (m), (n) and (o) The muscular stomach shows bleeding spots, with scattered small bleeding points at the junction of the muscular stomach and the glandular stomach, along with bleeding and edema in the glandular gastric papilla. (p) No abnormality in the

muscular stomach and the glandular stomach. (q), (r) and (s) Both the intestine and pancreas exhibit pronounced bleeding and necrosis throughout.. (t) No abnormality in the intestine and pancreas.
