# Supplementary material for: Genetic Characterization, Pathogenicity, and Epidemiology Analysis of Three Sub-Genotype Pigeon Newcastle Disease Virus Strains in China
Source: Microorganisms. 2024 Apr 4;12(4):738. doi: 10.3390/microorganisms12040738 (PMC11051795; doi:10.3390/microorganisms12040738)
Supplement: Supplementary file 1 [file microorganisms-12-00738-s001.zip › Supplement Table S1 Primers.pdf]

Table S1 Primers for sequencing the viral whole genome of the NDV in pigeons.

Primer	Location (nt)	Sequence (5'→3')	Size (bp)
A-1	1 – 1,530	ACCAAACAGAGAATCYGTGAG	1,530
A-2		TGAATGGTGCTCTGTGCAG	
B-1	1,304 – 2,790	GCTGCCCAACGAGTATCTGAGG	1,487
B-2		GAGCRACTGCYCGTAGATCAC	
C-1	2,552 – 4,056	TGTCGACTTTGTGCAGGC	1,505
C-2		AATGTCACTTTCTTTCC	
D-1	3,905 – 5,516	CCTCGCGCTCAATGTCACTA	1,612
D-2		CTACCTTCCGGGACAAGTG	
E-1	5,444 – 7,087	CGTGCCACCTACCTGGAGAC	1,644
E-2		GAGTAGAAAAGAATACCCTCCCTGT	
F-1	6,930 – 8,565	TTGCACTCGGATACCCTCA	1,636
F-2		TCAAGTATTTTCTTCCATT	
G-1	8,432 – 10,047	CCAGAGTCACATCTATCYTCYCCAT	1,616
G-2		AAAATCCGCCCATTAACRTT	
H-1	9,825 – 11,484	GGCTCGCCTCATTTAGGC	1,660
H-2		ACCGCTTCTTGATTGAGTAGGA	
I-1	11,237 – 12,944	CTGAGTCCTAGCATTATGACTA	1,708
I-2		AGTGAATRATGGGGTGAGAGAT	
J-1	12,839 – 14,455	AGAGGTCTCAACAACATCGTCTTAT	1,617
J-2		TCTCAAAWACTTCATTAGTCTCGGT	
K-1	13,956 – 15,192	TGACCTCRGATAAGGCAGTGAG	1,237
K-2		ACCAAACAAAGATTTGGTGAA	
