# Supplementary material for: Genetic Characterization, Pathogenicity, and Epidemiology Analysis of Three Sub-Genotype Pigeon Newcastle Disease Virus Strains in China
Source: Microorganisms. 2024 Apr 4;12(4):738. doi: 10.3390/microorganisms12040738 (PMC11051795; doi:10.3390/microorganisms12040738)
Supplement: Supplementary file 1 [file microorganisms-12-00738-s001.zip › Supplement Table S2 ref strains.pdf]

Table S2. Basic information and genotypes of 43 NDV reference strains.

Accession no.	Strains	Host	Origin	Year	Class	Sub/Genotype
FJ794269	NDV08-004	Duck	China	2008	I	1.1.1
KT223818	Qianghai01	Pigeon	China	2014	I	1.1.2
HQ008337	JS10	Duck	China	2010	I	1.2
JX524203	V4	Chicken	Australia	1966	II	I.1.1
GQ918280		Black headed gull	Sweden	1994	II	I.1.2.1
KC503476	136	Northern pintail	USA	2009	II	I.1.2.2
JX193077	16	Duck	China	2008	II	I.2
AF077761	Lasota	Chicken	USA	1946	II	II
EF201805	Mukteswar	Avian		1940	II	III
AY741404	Herts	Fowl	UK	1933	II	IV
AY582987	CA	Gamefowl	USA	2002	II	V.1
JX974435		Chicken	Mexico	2010	II	V.2
FJ410145	NY	Pigeon	USA	1984	II	VI.1
MG018211	1185	Eurasian collared dove	USA	2015	II	VI.2.1.1.1
FJ766527	JS16	Pigeon	China	2007	II	VI.2.1.1.2.1
MG840654	Ningxia2068	Pigeon	China	2016	II	VI.2.1.1.2.2
JX518532	B2Isiolo	Dove	Kenya	2012	II	VI.2.1.2
FJ410148	Texas	Pigeon	USA	1998	II	VI.2.2.1
KJ808820	SD	Pigeon	China	2012	II	VI.2.2.2
DQ659677	NA1	Goose	China	1999	II	VII.1.1
GQ338309	18	Pigeon	China	2003	II	VII.1.2
KT760569	Yunan1200	Goose	China	2013	II	VII.2
FJ751918	QH1	Chicken	China	1979	II	VIII
FJ436302	F48E8	Chicken	China	1948	II	IX
JN872171	17531-3	Turkey	USA	2010	II	X
HQ266603	MG1992	Ckicken	Madagascar	2008	II	XI
JN800306	1918_03	Chicken	Peru	2008	II	XII.1
KC551967	Guangdong	Goose	China	2010	II	XII.2
MF409241	Chiwoko	Chicken	Zambia	2015	II	XIII.1.1
JQ267579	EMM7	Chicken	Iran	2011	II	XIII.1.2
MH019281	PK1	Chicken	Pakistan	2015	II	XIII.2.1
MF422129	41016A	Chicken	India	2016	II	XIII.2.2
JF966386	ML029	Chicken	Mali	2007	II	XIV.1
KT948996	NG695	Duck	Nigeria	2009	II	XIV.2
JX119193	49931	Chicken	Dominican Republic	2008	II	XVI
KY171991	861	Quail	Nigeria	2004	II	XVII
JF966387	ML008	Chicken	Mali	2009	II	XVIII.1

MH392227	914	Chicken	Nigeria	2009	II	XVIII.2
JN941993	W241508	Pelican	USA	2008	II	XIX
AB853928	SM87	Chicken	Japan	1987	II	XX
KT962979	777	Pigeon	Russia	2010	II	XXI.1.1
KX236100	21A	Pigeon	Pakistan	2015	II	XXI.1.2
MH044693	Konarak Barin	Pigeon	Iran	2017	II	XXI.2
