# Supplementary material for: Genetic Characterization, Pathogenicity, and Epidemiology Analysis of Three Sub-Genotype Pigeon Newcastle Disease Virus Strains in China
Source: Microorganisms. 2024 Apr 4;12(4):738. doi: 10.3390/microorganisms12040738 (PMC11051795; doi:10.3390/microorganisms12040738)
Supplement: Supplementary file 1 [file microorganisms-12-00738-s001.zip › Supplement Table S3 GenBank database 1329.pdf]

Table S3. Basic information of 1329 pigeon NDV strains from GenBank database (accessed on February 6, 2024).

No	GenBank	Strain	Year	Region	Class	Genotype	Sub-genotype
1	AB070390.1	Avian_orthoavulavirus_1_APMV1/pigeon/Japan/FK-1/84	1984	Japan	Class II	VI	VI.2.1.1.1
2	AB070391.1	Avian_orthoavulavirus_1_APMV1/pigeon/Japan/Toyama/84	1984	Japan	Class II	VI	VI.2.1.1.1
3	AB070392.1	Avian_orthoavulavirus_1_APMV1/pigeon/Japan/Ibaraki/84	1984	Japan	Class II	VI	VI.1
4	AB070393.1	Avian_orthoavulavirus_1_APMV1/pigeon/Japan/Nagano-8/84	1984	Japan	Class II	VI	VI.2.1.1.1
5	AB070407.1	Avian_orthoavulavirus_1_APMV1/pigeon/Japan/Tochigi/86	1986	Japan	Class II	VI	VI.1
6	AB070409.1	Avian_orthoavulavirus_1_APMV1/pigeon/Japan/Niigata/88	1988	Japan	Class II	VI	VI.2.1.1.1
7	AB070412.1	Avian_orthoavulavirus_1_APMV1/pigeon/Japan/Kushiro/91	1991	Japan	Class II	VI	VI.2.1.1.1
8	AB070413.1	Avian_orthoavulavirus_1_APMV1/pigeon/Japan/Tokachi/91	1991	Japan	Class II	VI	VI.2.1.1.1
9	AB070416.1	Avian_orthoavulavirus_1_APMV1/pigeon/Japan/Ehime/93	1993	Japan	Class II	VI	VI.2.1.1.1
10	AB070417.1	Avian_orthoavulavirus_1_APMV1/pigeon/Japan/Kumamoto/95	1995	Japan	Class II	VI	VI.2.1.1.1
11	AB070419.1	Avian_orthoavulavirus_1_APMV1/pigeon/Japan/Tochigi/95	1995	Japan	Class II	VI	VI.2.1.1.1
12	AB070420.1	Avian_orthoavulavirus_1_APMV1/pigeon/Japan/Utsunomiya/95	1995	Japan	Class II	VI	VI.2.1.1.1
13	AB070422.1	Avian_orthoavulavirus_1_APMV1/pigeon/Japan/Shiga/96	1996	Japan	Class II	VI	VI.2.1.1.1
14	AB070423.1	Avian_orthoavulavirus_1_APMV1/pigeon/Japan/Fukushima/96	1996	Japan	Class II	VI	VI.2.1.1.1
15	AB070426.1	Avian_orthoavulavirus_1_APMV1/pigeon/Japan/Saitama/97	1997	Japan	Class II	VI	VI.2.1.1.1
16	AB070434.1	Avian_orthoavulavirus_1_APMV1/pigeon/Japan/Gunma/2000	2000	Japan	Class II	VI	VI.2.1.1.1
17	AF204754.1	Newcastle_disease_virus_isolate_APMV1/Pigeon/Kerala/India/NDV-KPG/_1998	1998	India	Class II	XXI	XXI.2
18	AF325434.1	Pigeon_paramyxovirus-1	2000	Argentina	Class II	XXI	XXI.2
19	AF358785.1	Newcastle_disease_virus_strain_Ch/98-1_	1998	China	Class II	VI	VI.2.2.2
20	AF452098.1	Newcastle_disease_virus	2001	USA	Class II	VI	VI.2.1.1.1
21	AF503647.1	Newcastle_disease_virus_pigeon/Portugal/8893/98	1998	Portugal	Class II	VI	VI.2.1.1.2.2
22	AF506762.1	Newcastle_disease_virus_strain_98/247	1998	Belgium	Class II	VI	VI.2.1.1.1

23	AF506763.1	Newcastle_disease_virus_strain_99/3	1999	Belgium	Class II	VI	VI.2.1.1.1
24	AF506764.1	Newcastle_disease_virus_strain_98/316	1998	Belgium	Class II	VI	VI.2.1.1.2.1
25	AF506765.1	Newcastle_disease_virus_strain_98/253	1998	Belgium	Class II	VI	VI.2.1.1.2.1
26	AF506766.1	Newcastle_disease_virus_strain_98/317	1998	Belgium	Class II	VI	VI.2.1.1.2.1
27	AF506767.1	Newcastle_disease_virus_strain_98/103	1998	Belgium	Class II	VI	VI.2.1.1.2.1
28	AF506768.1	Newcastle_disease_virus_strain_98/169	1998	Belgium	Class II	VI	VI.2.1.1.2.1
29	AF510738.1	Newcastle_disease_virus_isolate_2K7/Pigeon/Chennai/India	?	India	Class II	II	II
30	AF520965.1	Newcastle_disease_virus_isolate_2736/00	2000	Italy	Class II	VI	VI.2.1.1.1
31	AF520966.1	Newcastle_disease_virus_isolate_907/00	2000	Italy	Class II	VI	VI.1
32	AF520967.1	Newcastle_disease_virus_isolate_1166/00	2000	Italy	Class II	VI	VI.2.1.1.2.1
33	AF520968.1	Newcastle_disease_virus_isolate_1444/00	2000	Italy	Class II	VI	VI.1
34	AF520969.1	Newcastle_disease_virus_isolate_1811/00	2000	Italy	Class II	VI	VI.1
35	AF520970.1	Newcastle_disease_virus_isolate_4400/00	2000	Italy	Class II	VI	VI.1
36	AF520971.1	Newcastle_disease_virus_isolate_177/01	2001	Italy	Class II	VI	VI.2.1.1.1
37	AF520972.1	Newcastle_disease_virus_isolate_7007/01	2001	Italy	Class II	VI	VI.2.1.1.2.1
38	AJ306304.1	Newcastle_disease_virus_strain_99299	1999	France	Class II	VI	VI.2.1.1.2.1
39	AJ306305.1	Newcastle_disease_virus_strain_99106	1999	France	Class II	VI	VI.2.1.1.2.1
40	AJ415880.1	Pigeon_paramyxovirus-1_strain_99143	1999	France	Class II	II	II
41	AY008324.1	Newcastle_disease_virus_isolate_pigeon/1307/U.S./75	1975	USA	Class II	XIX	XIX
42	AY008325.1	Newcastle_disease_virus_isolate_pigeon/21042/U.S./98	1998	USA	Class II	XXI	XXI.2
43	AY008326.1	Newcastle_disease_virus_isolate_pigeon/3981/U.S.(MD)/84	1984	USA	Class II	XXI	XXI.1.1
44	AY008327.1	Newcastle_disease_virus_isolate_pigeon/44407/U.S./84	1984	USA	Class II	XIX	XIX
45	AY008328.1	Newcastle_disease_virus_isolate_pigeon/5658/U.S./75	1975	USA	Class II	V	V.3
46	AY008329.1	Newcastle_disease_virus_isolate_pigeon/NY/U.S./84	1984	USA	Class II	VI	VI.1
47	AY008330.1	Newcastle_disease_virus_isolate_pigeon/17498/U.S./98	1998	USA	Class II	VI	VI.2.2.1
48	AY130862.1	Newcastle_disease_virus_dove/U.S./9248-10/98	1998	USA	Class II	VII	VII.2

49	AY135742.1	Newcastle_disease_virus_isolate_BBGPI95039	1995	Bulgaria	Class II	XX	XX
50	AY135747.1	Newcastle_disease_virus_isolate_BITPI87079	1987	Italy	Class II	V	V.3
51	AY135749.1	Newcastle_disease_virus_isolate_PDEPI94102	1994	Germany	Class II	VI	VI.2.2.1
52	AY150092.1	Newcastle_disease_virus_strain_Iraq78	1978	Iraq	Class II	VI	VI.1
53	AY150093.1	Newcastle_disease_virus_strain_IT-227/82	1982	Italy	Class II	VI	VI.2.1.1.1
54	AY150094.1	Newcastle_disease_virus_strain_MT-217/82	1982	European	Class II	VI	VI.2.1.1.1
55	AY150095.1	Newcastle_disease_virus_strain_DE-209/83	1983	European	Class II	VI	VI.1
56	AY150096.1	Newcastle_disease_virus_strain_DE-302/83	1983	European	Class II	VI	VI.1
57	AY150097.1	Newcastle_disease_virus_strain_DK-279/83	1983	European	Class II	VI	VI.1
58	AY150098.1	Newcastle_disease_virus_strain_HU-58/83	1983	European	Class II	VI	VI.1
59	AY150099.1	Newcastle_disease_virus_strain_HU-61/83	1983	North American	Class II	VI	VI.2.2.1
60	AY150100.1	Newcastle_disease_virus_strain_SE-2/83	1983	European	Class II	VI	VI.1
61	AY150102.1	Newcastle_disease_virus_strain_HR-267/84	1984	European	Class II	VI	VI.1
62	AY150103.1	Newcastle_disease_virus_strain_HU-238/84	1984	North American	Class II	VI	VI.2.2.1
63	AY150105.1	Newcastle_disease_virus_strain_NO-1/85	1985	European	Class II	VI	VI.1
64	AY150107.1	Newcastle_disease_virus_strain_HU-342/86	1986	European	Class II	VI	VI.2.2.2
65	AY150108.1	Newcastle_disease_virus_strain_DE-33(MU)/87	1987	European	Class II	VI	VI.1
66	AY150109.1	Newcastle_disease_virus_strain_DE-35(MU)/87	1987	European	Class II	VI	VI.2.1.1.1
67	AY150110.1	Newcastle_disease_virus_strain_DE-47/87	1987	North American	Class II	VI	VI.2.2.1
68	AY150111.1	Newcastle_disease_virus_strain_EG-3/87	1987	European	Class II	VI	VI.2.1.1.1
69	AY150112.1	Newcastle_disease_virus_strain_EG-6/87	1987	European	Class II	VI	VI.2.1.1.1
70	AY150113.1	Newcastle_disease_virus_strain_HU-417/87	1987	North American	Class II	VI	VI.2.2.1

71	AY150114.1	Newcastle_disease_virus_strain_HU-491/87	1987	European	Class II	VI	VI.1
72	AY150115.1	Newcastle_disease_virus_strain_IT-125/87	1987	European	Class II	VI	VI.2.1.1.2.1
73	AY150116.1	Newcastle_disease_virus_strain_US-17/87	1987	North American	Class II	VI	VI.2.2.1
74	AY150117.1	Newcastle_disease_virus_strain_CA-24/88	1988	North American	Class II	VI	VI.2.2.1
75	AY150118.1	Newcastle_disease_virus_strain_HU-541/88	1988	European	Class II	VI	VI.1
76	AY150119.1	Newcastle_disease_virus_strain_CA-28/89	1989	North American	Class II	VI	VI.2.2.1
77	AY150120.1	Newcastle_disease_virus_strain_HU-655/89	1989	European	Class II	VI	VI.1
78	AY150121.1	Newcastle_disease_virus_strain_IT-132/89	1989	European	Class II	VI	VI.1
79	AY150122.1	Newcastle_disease_virus_strain_CA-41/90	1990	North American	Class II	VI	VI.2.2.1
80	AY150123.1	Newcastle_disease_virus_strain_DE-72/90	1990	European	Class II	VI	VI.2.1.1.1
81	AY150124.1	Newcastle_disease_virus_strain_DE-76/90	1990	North American	Class II	VI	VI.2.2.1
82	AY150125.1	Newcastle_disease_virus_strain_IT-137/90	1990	European	Class II	VI	VI.1
83	AY150126.1	Newcastle_disease_virus_strain_SE-3/90	1990	North American	Class II	VI	VI.2.2.1
84	AY150127.1	Newcastle_disease_virus_strain_CA-48/91	1991	North American	Class II	VI	VI.2.2.1
85	AY150128.1	Newcastle_disease_virus_strain_CA-56/92	1992	North American	Class II	VI	VI.2.2.1
86	AY150129.1	Newcastle_disease_virus_strain_DE-48/92	1992	European	Class II	VI	VI.2.1.1.1
87	AY150131.1	Newcastle_disease_virus_strain_DE-92/92	1992	European	Class II	VI	VI.2.1.1.2.1
88	AY150132.1	Newcastle_disease_virus_strain_ES-3/92	1992	European	Class II	VI	VI.2.1.1.1

89	AY150133.1	Newcastle_disease_virus_strain_DE-51/93	1993	European	Class II	VI	VI.2.1.1.1
90	AY150134.1	Newcastle_disease_virus_strain_DE-60/93	1993	European	Class II	VI	VI.2.1.1.1
91	AY150136.1	Newcastle_disease_virus_strain_DE-65/93	1993	North American	Class II	VI	VI.2.2.1
92	AY150137.1	Newcastle_disease_virus_strain_DE-97/93	1993	European	Class II	VI	VI.2.1.1.2.1
93	AY150138.1	Newcastle_disease_virus_strain_DK-12/93	1993	North American	Class II	VI	VI.2.2.1
94	AY150139.1	Newcastle_disease_virus_strain_DE-54/94	1994	European	Class II	VI	VI.2.1.1.1
95	AY150140.1	Newcastle_disease_virus_strain_DE-55/94	1994	European	Class II	VI	VI.2.1.1.1
96	AY150142.1	Newcastle_disease_virus_strain_IT-146/94	1994	European	Class II	VI	VI.1
97	AY150143.1	Newcastle_disease_virus_strain_DE-57/95	1995	European	Class II	VI	VI.2.1.1.1
98	AY150144.1	Newcastle_disease_virus_strain_HR-65/95	1995	European	Class II	XXI	XXI.1.1
99	AY150145.1	Newcastle_disease_virus_strain_IT-150/95	1995	European	Class II	VI	VI.1
100	AY150146.1	Newcastle_disease_virus_strain_IT-151/95	1995	European	Class II	VI	VI.1
101	AY150147.1	Newcastle_disease_virus_strain_IT-152/96	1996	European	Class II	VI	VI.1
102	AY150148.1	Newcastle_disease_virus_strain_IT-153/96	1996	European	Class II	VI	VI.1
103	AY150149.1	Newcastle_disease_virus_strain_DE-2653/98	1998	European	Class II	VI	VI.2.1.1.1
104	AY150150.1	Newcastle_disease_virus_strain_DE-2654/98	1998	European	Class II	VI	VI.2.1.1.1
105	AY150151.1	Newcastle_disease_virus_strain_DE-2663/98	1998	European	Class II	VI	VI.2.1.1.1
106	AY150152.1	Newcastle_disease_virus_strain_MX-11/98	1998	North American	Class II	VI	VI.2.2.1
107	AY150153.1	Newcastle_disease_virus_strain_DE-1383/99	1999	European	Class II	VI	VI.2.1.1.1
108	AY150154.1	Newcastle_disease_virus_strain_DE-690/00	2000	European	Class II	VI	VI.2.1.1.2.1
109	AY150155.1	Newcastle_disease_virus_strain_HU-1G/00	2000	European	Class II	VI	VI.2.1.1.2.1
110	AY150156.1	Newcastle_disease_virus_strain_SE-6/00	2000	European	Class II	VI	VI.2.1.1.2.1
111	AY150157.1	Newcastle_disease_virus_strain_SE-7/00	2000	European	Class II	VI	VI.2.1.1.2.1

112	AY150158.1	Newcastle_disease_virus_strain_SE-8/00	2000	European	Class II	VI	VI.2.1.1.2.1
113	AY150159.1	Newcastle_disease_virus_strain_SE-9/00	2000	European	Class II	VI	VI.2.1.1.2.1
114	AY150160.1	Newcastle_disease_virus_strain_DE-1266/01	2001	European	Class II	VI	VI.2.1.1.2.1
115	AY150161.1	Newcastle_disease_virus_strain_SE-10/01	2001	European	Class II	VI	VI.2.1.1.2.1
116	AY150162.1	Newcastle_disease_virus_strain_HR-111/01	2001	European	Class II	XXI	XXI.1.1
117	AY150163.1	Newcastle_disease_virus_strain_HR-155/01	2001	European	Class II	XXI	XXI.1.1
118	AY150164.1	Newcastle_disease_virus_strain_YU(Vo)-595/01	2001	European	Class II	VI	VI.2.1.1.2.1
119	AY150165.1	Newcastle_disease_virus_strain_HR-3/02	2002	European	Class II	XXI	XXI.1.1
120	AY190518.1	Newcastle_disease_virus_isolate_HU-1/02	2002	European	Class II	VI	VI.2.1.1.2.1
121	AY208697.1	Newcastle_disease_virus_isolate_PPMV1/pigeon/China/HLJ-4/02	2002	China	Class II	VI	VI.2.1.1.2.1
122	AY236999.1	Newcastle_disease_virus_Pigeon/ChennaiIndia/2k27/2002	2002	India	Class II	XIII	XIII.2.2
123	AY249136.1	Newcastle_disease_virus_isolate_pigeon/2k6/Chennai/India/2002	2002	India	Class II	II	II
124	AY252120.1	Newcastle_disease_virus_strain_HU-1114/90	1990	European	Class II	VI	VI.1
125	AY288995.1	Newcastle_disease_virus_dove/Italy/2736/00	2000	Italy	Class II	VI	VI.2.1.1.1
126	AY288996.1	Newcastle_disease_virus_pigeon/Italy/1166/00	2000	Italy	Class II	VI	VI.2.1.1.1
127	AY324318.1	Newcastle_disease_virus_isolate_APMV-1/2k6/Pigeon/Chennai/India	?	India	Class II	II	II
128	AY325798.1	Pigeon_paramyxovirus-1_precursor_	2003	China	Class II	VI	VI.2.2.2
129	AY372139.1	Newcastle_disease_virus_isolate_P85-4-26	1985	China	Class II	VI	VI.1
130	AY372141.1	Newcastle_disease_virus_isolate_P84-1-5	1984	China	Class II	VII	VII.1.2
131	AY372142.1	Newcastle_disease_virus_isolate_P95-2-17	1995	China	Class II	II	II
132	AY372143.1	Newcastle_disease_virus_isolate_P95-3-22	1995	China	Class II	VII	VII.1.2
133	AY372144.1	Newcastle_disease_virus_isolate_P95-3-23	1995	China	Class II	II	II
134	AY372145.1	Newcastle_disease_virus_isolate_P96-9-4	1996	China	Class II	VII	VII.1.2
135	AY378322.1	Newcastle_disease_virus_isolate_APMV-1/2k27/Pigeon/Chennai/India	?	India	Class II	XIII	XIII.2.2
136	AY390288.1	Newcastle_disease_virus_isolate_YZ9712	1997	China	Class II	VI	VI.2.2.2
137	AY390289.1	Newcastle_disease_virus_isolate_NC9701	1997	China	Class II	VI	VI.2.2.2

138	AY390290.1	Newcastle_disease_virus_isolate_NP9904	1999	China	Class II	VI	VI.2.2.2
139	AY390291.1	Newcastle_disease_virus_isolate_PB9601	1996	China	Class II	VI	VI.2.2.2
140	AY390292.1	Newcastle_disease_virus_isolate_SZa9803	1998	China	Class II	VI	VI.2.2.2
141	AY390314.1	Newcastle_disease_virus_strain_PK9910	1999	China	Class II	II	II
142	AY428960.1	Newcastle_disease_virus_PPMV1/Pigeon/CA/2003	2003	USA	Class II	VI	VI.2.1.1.1
143	AY438667.1	Newcastle_disease_virus_isolate_Dove/U.S.(CA)/9547/03	2003	USA	Class II	V	V.1
144	AY444501.1	Newcastle_disease_virus_strain_pigeon/U.S.(TX)/17498/98	1998	USA	Class II	VI	VI.2.2.1
145	AY445669.1	Newcastle_disease_virus_isolate_ZA469/PPMV1/02	2002	South Africa	Class II	VI	VI.2.1.1.1
146	AY471721.1	Newcastle_disease_virus_strain_PAEPI00377	2000	United Arab Emirates	Class II	I	I.1.1
147	AY471722.1	Newcastle_disease_virus_strain_PIEPI00246	2000	Ireland	Class II	VI	VI.2.1.1.2.1
148	AY471723.1	Newcastle_disease_virus_strain_PUKPI02430	2002	United Kingdom	Class II	VI	VI.2.1.1.2.1
149	AY471724.1	Newcastle_disease_virus_strain_-IEPI00242	2000	Ireland	Class II	VI	VI.2.1.1.2.1
150	AY471725.1	Newcastle_disease_virus_strain_PUKPI02428	2002	United Kingdom	Class II	VI	VI.2.1.1.2.2
151	AY471726.1	Newcastle_disease_virus_strain_PUKPI00250	2000	United Kingdom	Class II	VI	VI.2.1.1.2.1
152	AY471727.1	Newcastle_disease_virus_strain_PUKPI02429	2002	United Kingdom	Class II	VI	VI.2.1.1.2.2
153	AY471728.1	Newcastle_disease_virus_strain_PAEPI00318	2000	United Arab Emirates	Class II	VI	VI.2.1.1.2.1
154	AY471729.1	Newcastle_disease_virus_strain_PBEPI98389	1998	Belgium	Class II	VI	VI.2.1.1.2.1
155	AY471730.1	Newcastle_disease_virus_strain_PUKPI02423	2002	United Kingdom	Class II	VI	VI.2.1.1.2.1
156	AY471731.1	Newcastle_disease_virus_strain_PUKPI02433	2002	United	Class II	VI	VI.2.1.1.2.1

				Kingdom			
157	AY471732.1	Newcastle_disease_virus_strain_PIEPI01363	2001	Ireland	Class II	VI	VI.2.1.1.2.1
158	AY471733.1	Newcastle_disease_virus_strain_PIEPI01369	2001	Ireland	Class II	VI	VI.2.1.1.2.1
159	AY471734.1	Newcastle_disease_virus_strain_PUKPI02440	2002	United Kingdom	Class II	VI	VI.2.1.1.2.1
160	AY471735.1	Newcastle_disease_virus_strain_PCAPI91374	1991	Canada	Class II	VI	VI.2.1.1.2.1
161	AY471736.1	Newcastle_disease_virus_strain_PUKPI01421	2001	United Kingdom	Class II	VI	VI.2.1.1.2.1
162	AY471737.1	Newcastle_disease_virus_strain_PIEPI02436	2002	Ireland	Class II	VI	VI.2.1.1.2.2
163	AY471738.1	Newcastle_disease_virus_strain_PDKPI00316	2000	Germany	Class II	VI	VI.2.1.1.2.1
164	AY471739.1	Newcastle_disease_virus_strain_PDKPI01322	2001	Germany	Class II	VI	VI.2.1.1.2.1
165	AY471740.1	Newcastle_disease_virus_strain_PSEPI00317	2000	Sweden	Class II	VI	VI.2.1.1.2.1
166	AY471742.1	Newcastle_disease_virus_strain_PITDO01321	2001	Italy	Class II	VI	VI.2.1.1.2.1
167	AY471743.1	Newcastle_disease_virus_strain_PIEPI01387	2001	Ireland	Class II	VI	VI.2.1.1.2.1
168	AY471744.1	Newcastle_disease_virus_strain_PIEPI00339	2001	Ireland	Class II	VI	VI.2.1.1.2.1
169	AY471745.1	Newcastle_disease_virus_strain_PAEPI00366	2000	United Arab Emirates	Class II	VI	VI.2.1.1.2.1
170	AY471746.1	Newcastle_disease_virus_strain_PUKPI00248	2000	United Kingdom	Class II	VI	VI.2.1.1.2.1
171	AY471747.1	Newcastle_disease_virus_strain_PBEPI98327	1998	Belgium	Class II	VI	VI.2.1.1.2.1
172	AY471748.1	Newcastle_disease_virus_strain_PUKPI86240	1986	United Kingdom	Class II	VI	VI.1
173	AY471749.1	Newcastle_disease_virus_strain_PUKPI01376	2001	United Kingdom	Class II	VI	VI.2.1.1.2.1
174	AY471750.1	Newcastle_disease_virus_strain_PBEPI98328	1998	Belgium	Class II	VI	VI.2.1.1.2.1
175	AY471751.1	Newcastle_disease_virus_strain_PUKPI02422	2002	United	Class II	VI	VI.2.1.1.2.1

				Kingdom			
176	AY471752.1	Newcastle_disease_virus_strain_PUKPI01383	2001	United Kingdom	Class II	VI	VI.2.1.1.2.1
177	AY471753.1	Newcastle_disease_virus_strain_PUKPI01426	2001	United Kingdom	Class II	VI	VI.2.1.1.2.1
178	AY471754.1	Newcastle_disease_virus_strain_PUKPI02435	2002	United Kingdom	Class II	VI	VI.2.1.1.2.1
179	AY471755.1	Newcastle_disease_virus_strain_PUKPI02431	2002	United Kingdom	Class II	VI	VI.2.1.1.2.1
180	AY471756.1	Newcastle_disease_virus_strain_PUKPI02434	2002	United Kingdom	Class II	VI	VI.2.1.1.2.1
181	AY471757.1	Newcastle_disease_virus_strain_PDEPI95293	1995	Germany	Class II	VI	VI.2.1.1.1
182	AY471758.1	Newcastle_disease_virus_strain_PUKPI90213	1990	United Kingdom	Class II	VI	VI.2.1.1.1
183	AY471759.1	Newcastle_disease_virus_strain_PATPI95295	1995	Austria	Class II	VI	VI.2.1.1.1
184	AY471760.1	Newcastle_disease_virus_strain_PATPI98206	1998	Austria	Class II	VI	VI.2.1.1.1
185	AY471761.1	Newcastle_disease_virus_strain_PDKPI93202	1993	Germany	Class II	VI	VI.2.1.1.1
186	AY471762.1	Newcastle_disease_virus_strain_PDEPI94409	1994	Germany	Class II	VI	VI.2.1.1.1
187	AY471763.1	Newcastle_disease_virus_strain_PDEPI94216	1994	Germany	Class II	VI	VI.2.1.1.1
188	AY471764.1	Newcastle_disease_virus_strain_PUKPI94400	1994	United Kingdom	Class II	VI	VI.2.1.1.1
189	AY471765.1	Newcastle_disease_virus_strain_PFRPI98372	1998	France	Class II	VI	VI.2.1.1.1
190	AY471766.1	Newcastle_disease_virus_strain_PUKPI98355	1998	United Kingdom	Class II	VI	VI.2.1.1.1
191	AY471767.1	Newcastle_disease_virus_strain_PDEPI94217	1994	Germany	Class II	VI	VI.2.1.1.1
192	AY471768.1	Newcastle_disease_virus_strain_PIEPI96349	1996	Ireland	Class II	VI	VI.2.1.1.1

193	AY471769.1	Newcastle_disease_virus_strain_PITPI99232	1999	Italy	Class II	VI	VI.2.1.1.1
194	AY471771.1	Newcastle_disease_virus_strain_PATPI97205	1997	Austria	Class II	VI	VI.2.1.1.1
195	AY471774.1	Newcastle_disease_virus_strain_PPTPI01315	2001	Portugal	Class II	VI	VI.2.1.1.2.1
196	AY471775.1	Newcastle_disease_virus_strain_PPTPI99395	1999	Portugal	Class II	VI	VI.2.1.1.2.1
197	AY471776.1	Newcastle_disease_virus_strain_PPTPI98388	1998	Portugal	Class II	VI	VI.2.1.1.2.2
198	AY471777.1	Newcastle_disease_virus_strain_PPTPI99393	1999	Portugal	Class II	VI	VI.2.1.1.2.1
199	AY471778.1	Newcastle_disease_virus_strain_PATPI97345	1997	Austria	Class II	VI	VI.2.1.1.2.1
200	AY471779.1	Newcastle_disease_virus_strain_PAEPI98367	1998	United Arab Emirates	Class II	VI	VI.2.1.2
201	AY471780.1	Newcastle_disease_virus_strain_PAEPI99361	1999	United Arab Emirates	Class II	VI	VI.2.1.2
202	AY471781.1	Newcastle_disease_virus_strain_PAEPI96210	1996	United Arab Emirates	Class II	VI	VI.2.1.2
203	AY471782.1	Newcastle_disease_virus_strain_PAEPI97415	1997	United Arab Emirates	Class II	VI	VI.2.1.2
204	AY471786.1	Newcastle_disease_virus_strain_PSAPI98390	1998	Saudi Arabia	Class II	VI	VI.2.1.2
205	AY471788.1	Newcastle_disease_virus_strain_PILPI01381	2001	Israel	Class II	VI	VI.2.1.2
206	AY471789.1	Newcastle_disease_virus_strain_PATPI00323	2000	Austria	Class II	XXI	XXI.1.1
207	AY471790.1	Newcastle_disease_virus_strain_PUKPI89228	1989	United Kingdom	Class II	VI	VI.2.2.1
208	AY471791.1	Newcastle_disease_virus_strain_PUKPI89283	1989	United Kingdom	Class II	VI	VI.2.2.1
209	AY471792.1	Newcastle_disease_virus_strain_PUKPI90218	1990	United Kingdom	Class II	VI	VI.1
210	AY471793.1	Newcastle_disease_virus_strain_PUKPI88286	1988	United	Class II	VI	VI.2.2.1

				Kingdom			
211	AY471794.1	Newcastle_disease_virus_strain_PFIPI92333	1992	Finland	Class II	VI	VI.2.2.1
212	AY471795.1	Newcastle_disease_virus_strain_PCAPI93347	1993	Canada	Class II	VI	VI.2.2.1
213	AY471796.1	Newcastle_disease_virus_strain_PCAPI90411	1990	Canada	Class II	VI	VI.2.2.1
214	AY471797.1	Newcastle_disease_virus_strain_PCAPI91412	1991	Canada	Class II	VI	VI.2.2.1
215	AY471798.1	Newcastle_disease_virus_strain_PUKPI90236	1990	United Kingdom	Class II	VI	VI.1
216	AY471799.1	Newcastle_disease_virus_strain_PUKPI91344	1991	United Kingdom	Class II	VI	VI.1
217	AY471800.1	Newcastle_disease_virus_strain_PUKPI91233	1991	United Kingdom	Class II	VI	VI.1
218	AY471801.1	Newcastle_disease_virus_strain_PUKPI90221	1990	United Kingdom	Class II	VI	VI.1
219	AY471802.1	Newcastle_disease_virus_strain_PUKPI90222	1990	United Kingdom	Class II	VI	VI.1
220	AY471803.1	Newcastle_disease_virus_strain_PUKPI90230	1990	United Kingdom	Class II	VI	VI.1
221	AY471804.1	Newcastle_disease_virus_strain_PUKPI90237	1990	United Kingdom	Class II	VI	VI.1
222	AY471805.1	Newcastle_disease_virus_strain_PUKPI91353	1991	United Kingdom	Class II	VI	VI.1
223	AY471807.1	Newcastle_disease_virus_strain_PIEPI92332	1992	Ireland	Class II	VI	VI.1
224	AY471810.1	Newcastle_disease_virus_strain_PUKPI90397	1990	United Kingdom	Class II	VI	VI.1
225	AY471811.1	Newcastle_disease_virus_strain_PUKPI--417	?	United Kingdom	Class II	VI	VI.1

226	AY471812.1	Newcastle_disease_virus_strain_PUKPI--418	?	United Kingdom	Class II	VI	VI.1
227	AY471813.1	Newcastle_disease_virus_strain_PUKPI93399	1993	United Kingdom	Class II	VI	VI.2.1.1.1
228	AY471814.1	Newcastle_disease_virus_strain_PUKPI93404	1993	United Kingdom	Class II	VI	VI.1
229	AY471815.1	Newcastle_disease_virus_strain_PIEPI91375	1991	Ireland	Class II	VI	VI.2.1.1.1
230	AY471816.1	Newcastle_disease_virus_strain_PUKPI93396	1993	United Kingdom	Class II	VI	VI.1
231	AY471817.1	Newcastle_disease_virus_strain_PUKDO96438	1996	United Kingdom	Class II	VI	VI.1
232	AY471819.1	Newcastle_disease_virus_strain_PUKPI90214	1990	United Kingdom	Class II	VI	VI.1
233	AY471820.1	Newcastle_disease_virus_strain_PUKPI88256	1988	United Kingdom	Class II	VI	VI.1
234	AY471821.1	Newcastle_disease_virus_strain_PUKPI88284	1988	United Kingdom	Class II	VI	VI.1
235	AY471822.1	Newcastle_disease_virus_strain_PUKPI88290	1988	United Kingdom	Class II	VI	VI.1
236	AY471823.1	Newcastle_disease_virus_strain_PUKPI88238	1988	United Kingdom	Class II	VI	VI.1
237	AY471824.1	Newcastle_disease_virus_strain_PUKPI86244	1986	United Kingdom	Class II	VI	VI.1
238	AY471825.1	Newcastle_disease_virus_strain_PUKPI90275	1990	United Kingdom	Class II	VI	VI.1
239	AY471826.1	Newcastle_disease_virus_strain_PUKPI89229	1989	United	Class II	VI	VI.1

				Kingdom			
240	AY471827.1	Newcastle_disease_virus_strain_PUKPI89231	1989	United Kingdom	Class II	VI	VI.1
241	AY471828.1	Newcastle_disease_virus_strain_PUKPI90280	1990	United Kingdom	Class II	VI	VI.1
242	AY471829.1	Newcastle_disease_virus_strain_PIEPI90215	1990	Ireland	Class II	VI	VI.1
243	AY471830.1	Newcastle_disease_virus_strain_PUKPI88224	1988	United Kingdom	Class II	VI	VI.1
244	AY471831.1	Newcastle_disease_virus_strain_PUKPI88249	1988	United Kingdom	Class II	VI	VI.1
245	AY471832.1	Newcastle_disease_virus_strain_PUKPI88234	1988	United Kingdom	Class II	VI	VI.1
246	AY471833.1	Newcastle_disease_virus_strain_PUKPI86247	1986	United Kingdom	Class II	VI	VI.1
247	AY471834.1	Newcastle_disease_virus_strain_PHUPI84357	1984	Hungary	Class II	VI	VI.1
248	AY471835.1	Newcastle_disease_virus_strain_PUKPI94401	1994	United Kingdom	Class II	VI	VI.2.1.1.1
249	AY471836.1	Newcastle_disease_virus_strain_PUKPI89226	1989	United Kingdom	Class II	VI	VI.2.1.1.1
250	AY471837.1	Newcastle_disease_virus_strain_PITPI94406	1994	Italy	Class II	VI	VI.1
251	AY471838.1	Newcastle_disease_virus_strain_PITPI98371	1998	Italy	Class II	VI	VI.1
252	AY471840.1	Newcastle_disease_virus_strain_PITPI97341	1997	Italy	Class II	VI	VI.1
253	AY471841.1	Newcastle_disease_virus_strain_PITPI96407	1996	Italy	Class II	VI	VI.1
254	AY471842.1	Newcastle_disease_virus_strain_PITPI90414	1990	Italy	Class II	VI	VI.1
255	AY471844.1	Newcastle_disease_virus_strain_PATPI96335	1996	Austria	Class II	VI	VI.1
256	AY471845.1	Newcastle_disease_virus_strain_PATPI96413	1996	Austria	Class II	VI	VI.1

257	AY471846.1	Newcastle_disease_virus_strain_PITDO00289	2000	Italy	Class II	VI	VI.1
258	AY471847.1	Newcastle_disease_virus_strain_PUKPI84259	1984	United Kingdom	Class II	VI	VI.2.1.1.1
259	AY471848.1	Newcastle_disease_virus_strain_PUKPI84260	1984	United Kingdom	Class II	VI	VI.2.1.1.1
260	AY471849.1	Newcastle_disease_virus_strain_PBEPI84352	1984	Belgium	Class II	VI	VI.1
261	AY471850.1	Newcastle_disease_virus_strain_PITPI84354	1984	Italy	Class II	VI	VI.2.1.1.1
262	AY471851.1	Newcastle_disease_virus_strain_PUKPI83264	1983	United Kingdom	Class II	VI	VI.2.1.1.1
263	AY471852.1	Newcastle_disease_virus_strain_PUKPI83299	1983	United Kingdom	Class II	VI	VI.2.1.1.1
264	AY471854.1	Newcastle_disease_virus_strain_PUKPI86239	1986	United Kingdom	Class II	VI	VI.1
265	AY471855.1	Newcastle_disease_virus_strain_PUKPI84342	1984	United Kingdom	Class II	VI	VI.2.1.1.1
266	AY471856.1	Newcastle_disease_virus_strain_PHKPI86358	1986	China	Class II	VI	VI.1
267	AY471857.1	Newcastle_disease_virus_strain_PIQPI78442	1978	Iraq	Class II	VI	VI.1
268	AY581301.1	Newcastle_disease_virus_isolate_PiAD388/Quail/India	?	India	Class II	VI	VI.2.1.1.2.2
269	AY581302.1	Newcastle_disease_virus_isolate_PiAD33/Guinea_fowl/India	?	India	Class II	XIII	XIII.1.2
270	AY581303.1	Newcastle_disease_virus_isolate_PiAD18/Guinea_fowl/India	?	India	Class II	XIII	XIII.1.2
271	AY581304.1	Newcastle_disease_virus_isolate_PiAD55/Pigeon/India	?	India	Class II	XXI	XXI.1.2
272	AY581305.1	Newcastle_disease_virus_isolate_PiADPrd/Pigeon/India	?	India	Class II	XXI	XXI.1.2
273	AY635814.1	Newcastle_disease_virus_isolate_Gxp22	?	China	Class II	VI	VI.2.1.1.2.1
274	AY635815.1	Newcastle_disease_virus_isolate_Gxp40	?	China	Class II	VII	VII.1.1
275	AY734535.1	Newcastle_disease_virus_isolate_Pigeon/Argentina/Tigre_6/99	1999	Argentina	Class II	XXI	XXI.2
276	AY734536.1	Newcastle_disease_virus_isolate_Pigeon/Argentina/Capital_3/97	1997	Argentina	Class II	VI	VI.2.1.2

277	DQ417113.1	Newcastle_disease_virus_isolate_STP96_	1996	China	Class II	VI	VI.2.2.2
278	DQ485274.1	Newcastle_disease_virus_isolate_dove/Guangxi15/2005	2005	China	Class II	I	I.1.1
279	DQ904250.1	Newcastle_disease_virus_strain_NDV/pigeon/QC/SHY-01922/2006	2006	Canada	Class II	VI	VI.2.1.1.1
280	EF026584.1	Newcastle_disease_virus_strain_248_VB	1998	Belgium	Class II	VI	VI.2.1.1.2.1
281	EF030950.1	Newcastle_disease_virus_strain_PIZA06N642	2006	South Africa	Class II	VI	VI.2.1.1.1
282	EF030951.1	Newcastle_disease_virus_strain_CKZA06N606	2005	South Africa	Class II	VI	VI.2.1.1.2.1
283	EF030952.1	Newcastle_disease_virus_strain_DOZA05AM68313	2005	South Africa	Class II	I	I.1.1
284	EF030953.1	Newcastle_disease_virus_strain_DOZA05N240	2005	South Africa	Class II	VI	VI.2.1.1.2.1
285	EF030954.1	Newcastle_disease_virus_strain_DOZA05N247	2005	South Africa	Class II	VI	VI.2.1.1.1
286	EF030955.1	Newcastle_disease_virus_strain_DOZA05N417	2005	South Africa	Class II	VI	VI.2.1.1.1
287	EF030956.1	Newcastle_disease_virus_strain_DOZA05N539	2005	South Africa	Class II	VI	VI.2.1.1.1
288	EF030957.1	Newcastle_disease_virus_strain_DOZA06N549	2006	South Africa	Class II	VI	VI.2.1.1.1
289	EF030958.1	Newcastle_disease_virus_strain_DOZA06N589	2006	South Africa	Class II	VI	VI.2.1.1.1
290	EF030959.1	Newcastle_disease_virus_strain_DOZA06N591	2006	South Africa	Class II	VI	VI.2.1.1.2.1
291	EF030960.1	Newcastle_disease_virus_strain_DOZA06N621	2006	South Africa	Class II	VI	VI.2.1.1.1
292	EF030961.1	Newcastle_disease_virus_strain_DOZA06UP470	2006	South Africa	Class II	VI	VI.2.1.1.2.1
293	EF030962.1	Newcastle_disease_virus_strain_PIZA04N230	2004	South Africa	Class II	VI	VI.2.1.1.1
294	EF030963.1	Newcastle_disease_virus_strain_PIZA05N277	2005	South Africa	Class II	VI	VI.2.1.1.2.1
295	EF030964.1	Newcastle_disease_virus_strain_PIZA06N635	2006	South Africa	Class II	VI	VI.2.1.1.1
296	EF364037.1	Newcastle_disease_virus_isolate_Vh_1342-05	2005	Slovakia	Class II	VI	VI.2.1.1.2.1
297	EF364038.1	Newcastle_disease_virus_isolate_Vh_1343-05	2005	Slovakia	Class II	VI	VI.2.1.1.2.1
298	EF520716.1	Newcastle_disease_virus_strain_pigeon/NY/US/1984	1984	USA	Class II	VI	VI.1
299	EF534701.1	Newcastle_disease_virus_nonfunctional	?	Brazil	Class II	V	V.1
300	EF589137.1	Newcastle_disease_virus_strain_GZ	?	China	Class II	XX	XX
301	EU240576.1	Newcastle_disease_virus_isolate_Vh_357-06	2006	Slovakia	Class II	VI	VI.2.1.1.2.1
302	EU240578.1	Newcastle_disease_virus_isolate_Vh_448-06	2006	Slovakia	Class II	VI	VI.2.1.1.2.1

303	EU240580.1	Newcastle_disease_virus_isolate_Vh_529-06	2006	Slovakia	Class II	VI	VI.2.1.1.2.1
304	EU240582.1	Newcastle_disease_virus_isolate_Vh_536-06	2006	Slovakia	Class II	VI	VI.2.1.1.2.1
305	EU240583.1	Newcastle_disease_virus_isolate_Vh_538-06	2006	Slovakia	Class II	VI	VI.2.1.1.2.1
306	EU416228.1	Newcastle_disease_virus_isolate_SCP1	?	China	Class II	VI	VI.2.2.2
307	EU477188.1	Avian_orthoavulavirus_1_strain_dove/US/TX-B2580/2004	2004	USA	Class II	VI	VI.2.1.1.1
308	EU477189.1	Avian_orthoavulavirus_1_strain_pigeon/US/RI166/2000	2000	USA	Class II	VI	VI.2.1.1.1
309	EU477190.1	Avian_orthoavulavirus_1_strain_pigeon/US/TX3503/2004	2004	USA	Class II	VI	VI.2.1.1.1
310	EU477191.1	Avian_orthoavulavirus_1_strain_Eurasian_collared_dove/US/TX3988/2004	2004	USA	Class II	VI	VI.2.1.1.1
311	EU477192.1	Avian_orthoavulavirus_1_strain_Eurasian_collared_dove/US/TX4156/2005	2005	USA	Class II	VI	VI.2.1.1.1
312	EU477193.1	Avian_orthoavulavirus_1_strain_Eurasian_collared_dove/US/TX2334/2003	2003	USA	Class II	VI	VI.2.1.1.1
313	EU477194.1	Avian_orthoavulavirus_1_strain_Eurasian_collared_dove/US/TX3817/2004	2004	USA	Class II	VI	VI.2.1.1.1
314	EU477195.2	Avian_orthoavulavirus_1_strain_Eurasian_collared_dove/US/TX6295/2006	2006	USA	Class II	VI	VI.2.1.1.1
315	EU477196.1	Avian_orthoavulavirus_1_strain_Eurasian_collared_dove/US/TX6306/2007	2007	USA	Class II	VI	VI.2.1.1.1
316	EU477197.1	Avian_orthoavulavirus_1_strain_Eurasian_collared_dove/US/TX6338/2007	2007	USA	Class II	VI	VI.2.1.1.1
317	EU477198.1	Avian_orthoavulavirus_1_strain_pigeon/US/TX3377/2004	2004	USA	Class II	VI	VI.2.1.1.1
318	EU477199.1	Avian_orthoavulavirus_1_strain_Eurasian_collared_dove/US/TX3908/2004	2004	USA	Class II	VI	VI.2.1.1.1
319	EU477200.1	Avian_orthoavulavirus_1_strain_mourning_dove/US/TX4048/2004	2004	USA	Class II	VI	VI.2.1.1.1
320	EU477201.1	Avian_orthoavulavirus_1_strain_Eurasian_collared_dove/US/TX4078/2004	2004	USA	Class II	VI	VI.2.1.1.1
321	EU477202.1	Avian_orthoavulavirus_1_strain_pigeon/US/TX4142/2005	2005	USA	Class II	VI	VI.2.1.1.1
322	EU518682.1	Avian_orthoavulavirus_1_isolate_dove/Mexico(Distrito_Federal)/462/2004	2004	Mexico	Class II	V	V.3
323	EU780902.1	Newcastle_disease_virus_isolate_NDV/pigeon/Dnipro/2007	2007	Ukraine	Class II	I	I.1.1
324	FJ410145.1	Pigeon_paramyxovirus-1_strain_PPMV-1/New_York/1984	1984	USA	Class II	VI	VI.1
325	FJ410147.1	Pigeon_paramyxovirus-1_strain_PPMV-1/Maryl/1984	1984	USA	Class II	VI	VI.1
326	FJ410148.1	Pigeon_paramyxovirus-1_strain_PPMV-1/Texas/1998	1998	USA	Class II	VI	VI.2.2.1
327	FJ480825.1	Newcastle_disease_virus_strain_PG/CH/JS/1/05	2005	China	Class II	VI	VI.2.2.2
328	FJ480826.1	Newcastle_disease_virus_strain_PG/CH/JS/1/06	2006	China	Class II	VI	VI.2.2.2

329	FJ766526.1	Newcastle_disease_virus_isolate_JS/07/22/Pi	2007	China	Class II	VI	VI.2.1.1.2.1
330	FJ766527.1	Newcastle_disease_virus_isolate_JS/07/16/Pi	2007	China	Class II	VI	VI.2.1.1.2.1
331	FJ766530.1	Newcastle_disease_virus_isolate_JS/07/04/Pi	2007	China	Class II	VI	VI.2.1.1.2.1
332	FJ766531.1	Newcastle_disease_virus_isolate_JS/07/03/Pi	2007	China	Class II	VI	VI.2.1.1.2.1
333	FJ865434.1	Pigeon_paramyxovirus-1_strain_s-1	2002	China	Class II	VI	VI.1
334	FJ986192.2	Newcastle_disease_virus_isolate_2K3/Chennai/Tamil_Nadu	2000	India	Class II	IV	IV
335	FM200797.1	Newcastle_disease_virus_partialstrain_pigeon/Nigeria/NIE95/2007	2007	Nigeria	Class II	VI	VI.2.1.2
336	GQ168928.1	Newcastle_disease_virus_strain_JSP0204	2002	China	Class II	VI	VI.2.2.2
337	GQ245801.1	Newcastle_disease_virus_isolate_SD-15-08-_Pi	2008	China	Class II	VII	VII.1.1
338	GQ281085.1	Newcastle_disease_virus_isolate_JS-35-07-Pi	2007	China	Class II	VI	VI.2.1.1.2.1
339	GQ281086.1	Newcastle_disease_virus_isolate_WX-10-07-Pi	2007	China	Class II	VI	VI.2.1.1.2.1
340	GQ281087.1	Newcastle_disease_virus_isolate_YZ-21-07-Pi	2007	China	Class II	VI	VI.2.1.1.2.1
341	GQ281088.1	Newcastle_disease_virus_isolate_YZ-23-07-Pi	2007	China	Class II	VI	VI.2.1.1.2.1
342	GQ429292.1	Pigeon_paramyxovirus-1_strain_AV324/96_	1996	Ireland	Class II	VI	VI.2.1.1.1
343	GQ429293.1	Newcastle_disease_virus_isolate_dove/Italy/2736/00	2000	Italy	Class II	VI	VI.2.1.1.1
344	GU002424.1	Newcastle_disease_virus_strain_SLO-304/03	2003	Slovenia	Class II	VI	VI.1
345	GU002426.1	Newcastle_disease_virus_strain_SLO-308/03	2003	Slovenia	Class II	VI	VI.1
346	GU002428.1	Newcastle_disease_virus_strain_SLO-17/04	2004	Slovenia	Class II	VI	VI.2.1.1.2.1
347	GU002430.1	Newcastle_disease_virus_strain_SLO-263/04	2004	Slovenia	Class II	VI	VI.2.1.1.2.2
348	GU002432.1	Newcastle_disease_virus_strain_SLO-872/04	2004	Slovenia	Class II	VI	VI.2.1.1.2.1
349	GU002434.1	Newcastle_disease_virus_strain_SLO-218/05	2005	Slovenia	Class II	VI	VI.2.1.1.2.1
350	GU002436.1	Newcastle_disease_virus_strain_SLO-718/05	2005	Slovenia	Class II	VI	VI.2.1.1.2.1
351	GU002438.1	Newcastle_disease_virus_strain_SLO-802/05	2005	Slovenia	Class II	VI	VI.2.1.1.2.1
352	GU002442.1	Newcastle_disease_virus_strain_SLO-75/06	2006	Slovenia	Class II	VI	VI.2.1.1.2.1
353	GU002444.1	Newcastle_disease_virus_strain_SLO-912/08	2008	Slovenia	Class II	VI	VI.2.1.1.2.2
354	GU227738.1	Newcastle_disease_virus_strain_NDV/Serbia/749/2007	2007	Serbia	Class II	VII	VII.1.1

355	GU551934.1	Newcastle_disease_virus_strain_carrier-pigeon/Guangdong/2008	2008	China	Class II	VI	VI.2.1.1.2.1
356	GU591896.1	Newcastle_disease_virus_isolate_NDV09-001	2009	China	Class II	II	II
357	GU591897.1	Newcastle_disease_virus_isolate_NDV09-002	2009	China	Class II	II	II
358	HE972209.1	Newcastle_disease_virusgenomic_RNA_strain_APMV-1/pigeon/Luxembourg/119/2006	2006	Luxembourg	Class II	VI	VI.2.1.1.2.1
359	HE972212.1	Newcastle_disease_virusgenomic_RNA_strain_APMV-1/pigeon/Luxembourg/3821-1/2007	2007	Luxembourg	Class II	VI	VI.2.1.1.2.1
360	HE972216.1	Newcastle_disease_virus_partialgenomic_RNA_strain_APMV-1/pigeon/Luxembourg/2657-2/2006	2006	Luxembourg	Class II	I	I.1.1
361	HG326601.1	Newcastle_disease_virus_viral_strain_pigeon/Nigeria/NIE07-061/2007	2007	Nigeria	Class II	VI	VI.2.1.2
362	HG326602.1	Newcastle_disease_virus_viral_strain_pigeon/Nigeria/NIE07-062/2007	2007	Nigeria	Class II	VI	VI.2.1.2
363	HG326603.1	Newcastle_disease_virus_viral_strain_pigeon/Nigeria/NIE07-063/2007	2007	Nigeria	Class II	VI	VI.2.1.2
364	HG326604.1	Newcastle_disease_virus_viral_strain_pigeon/Nigeria/NIE09-1898/2009	2009	Nigeria	Class II	VI	VI.2.1.2
365	HG424625.1	Newcastle_disease_virus_strain_pigeon/Nigeria/NIE13-005/2013	2013	Nigeria	Class II	XXI	XXI.2
366	HG424626.1	Newcastle_disease_virus_strain_pigeon/Nigeria/NIE13-008/2013	2013	Nigeria	Class II	XXI	XXI.2
367	HG424627.1	Newcastle_disease_virus_strain_pigeon/Nigeria/NIE13-092/2013	2013	Nigeria	Class II	VI	VI.2.1.2
368	HG424628.1	Newcastle_disease_virus_strain_pigeon/Nigeria/NIE13-093/2013	2013	Nigeria	Class II	VI	VI.2.1.2
369	HG424629.1	Newcastle_disease_virus_strain_pigeon/Nigeria/NIE13-009/2013	2013	Nigeria	Class II	VI	VI.2.1.2
370	HM001215.1	Newcastle_disease_virus_isolate_P-DQ00-4	2000	China	Class II	VI	VI.2.2.2
371	HM001216.1	Newcastle_disease_virus_isolate_P-QD98-11	1998	China	Class II	VII	VII.1.1
372	HM063425.1	Newcastle_disease_virus_isolate_P4_	1999	China	Class II	VI	VI.2.1.1.2.1
373	HM627541.1	Newcastle_disease_virus_isolate_PPMV-1/PL/H2/10	2010	Poland	Class II	XXI	XXI.1.1
374	HM776583.1	Newcastle_disease_virus_isolate_JN08	2008	China	Class II	VI	VI.2.1.1.2.1
375	HQ456873.1	Newcastle_disease_virus_isolate_NGPG5207	2007	Nigeria	Class II	XVII	XVII
376	HQ847859.1	Pigeon_paramyxovirus_1_strain_W01-121	2001	USA	Class II	VI	VI.2.1.1.1
377	JF713701.1	Newcastle_disease_virus_isolate_SD10	2010	China	Class II	VI	VI.2.1.1.2.1
378	JF749831.1	Newcastle_disease_virus_isolate_Pigeon/Uruguay/Montevideo/02/08	2008	Uruguay	Class II	VI	VI.2.1.2
379	JF823992.1	Avian_orthoavulavirus_1_isolate_Pi/Rus/Moscow/47/02	2002	Russia	Class II	VI	VI.2.1.1.2.2
380	JF823993.1	Avian_orthoavulavirus_1_isolate_Pi/Rus/Kemerovo/762-1/05	2005	Russia	Class II	VI	VI.2.1.1.2.2

381	JF823994.1	Avian_orthoavulavirus_1_isolate_Pi/Rus/Kemerovo/762-2/05	2005	Russia	Class II	VI	VI.2.1.1.2.2
382	JF823995.1	Avian_orthoavulavirus_1_isolate_Pi/Rus/Vladimir/772/06	2006	Russia	Class II	VI	VI.2.1.1.2.1
383	JF823997.1	Avian_orthoavulavirus_1_isolate_Pi/Rus/Voronezh/446/01	2001	Russia	Class II	VI	VI.2.2.1
384	JF823998.1	Avian_orthoavulavirus_1_isolate_Pi/Rus/Moscow/54/02	2002	Russia	Class II	VI	VI.2.2.1
385	JF823999.1	Avian_orthoavulavirus_1_isolate_Pi/Rus/Altay/446/04	2004	Russia	Class II	XXI	XXI.1.1
386	JF824000.1	Avian_orthoavulavirus_1_isolate_Pi/Rus/Altay/447/04	2004	Russia	Class II	XXI	XXI.1.1
387	JF824001.1	Avian_orthoavulavirus_1_isolate_Pi/Rus/Kemerovo/754-1/05	2005	Russia	Class II	XXI	XXI.1.1
388	JF824002.1	Avian_orthoavulavirus_1_isolate_Pi/Rus/Kemerovo/754-2/05	2005	Russia	Class II	XXI	XXI.1.1
389	JF824003.1	Avian_orthoavulavirus_1_isolate_Pi/Rus/Kemerovo/754-3/05	2005	Russia	Class II	XXI	XXI.1.1
390	JF824004.1	Avian_orthoavulavirus_1_isolate_Pi/Rus/Kemerovo/754-4/05	2005	Russia	Class II	XXI	XXI.1.1
391	JF824005.1	Avian_orthoavulavirus_1_isolate_Pi/Rus/Kemerovo/0004/09	2009	Russia	Class II	XXI	XXI.1.1
392	JF824006.1	Avian_orthoavulavirus_1_isolate_Pi/Rus/Altay/448/04	2004	Russia	Class II	XXI	XXI.1.1
393	JF824007.1	Avian_orthoavulavirus_1_isolate_Pi/Rus/Kemerovo/563-1/05	2005	Russia	Class II	XXI	XXI.1.1
394	JF824008.1	Avian_orthoavulavirus_1_isolate_Pi/Rus/Kemerovo/563-3/05	2005	Russia	Class II	XXI	XXI.1.1
395	JF824009.1	Avian_orthoavulavirus_1_isolate_Pi/Rus/Kemerovo/563-5/05	2005	Russia	Class II	XXI	XXI.1.1
396	JF824010.1	Avian_orthoavulavirus_1_isolate_Pi/Rus/Kemerovo/592-1/05	2005	Russia	Class II	XXI	XXI.1.1
397	JF824011.1	Avian_orthoavulavirus_1_isolate_Pi/Rus/Kemerovo/592-2/05	2005	Russia	Class II	XXI	XXI.1.1
398	JF824012.1	Avian_orthoavulavirus_1_isolate_Pi/Rus/Kemerovo/593-2/05	2005	Russia	Class II	XXI	XXI.1.1
399	JF824013.1	Avian_orthoavulavirus_1_isolate_Pi/Rus/Kemerovo/0267/09	2009	Russia	Class II	XXI	XXI.1.1
400	JF824014.1	Avian_orthoavulavirus_1_isolate_Pi/Rus/Kemerovo/0263/09	2009	Russia	Class II	XXI	XXI.1.1
401	JF824015.1	Avian_orthoavulavirus_1_isolate_Pi/Rus/Kemerovo/0265/09	2009	Russia	Class II	XXI	XXI.1.1
402	JF824016.1	Avian_orthoavulavirus_1_isolate_Pi/Rus/Kemerovo/0268/09	2009	Russia	Class II	XXI	XXI.1.1
403	JF824017.1	Avian_orthoavulavirus_1_isolate_Pi/Rus/Moscow/810/06	2006	Russia	Class II	XXI	XXI.1.1
404	JF824018.1	Avian_orthoavulavirus_1_isolate_Pi/Rus/Moscow/407/04	2004	Russia	Class II	XXI	XXI.1.1
405	JF824019.1	Avian_orthoavulavirus_1_isolate_Pi/Rus/Moscow/424/04	2004	Russia	Class II	XXI	XXI.1.1
406	JF824020.1	Avian_orthoavulavirus_1_isolate_Pi/Rus/Moscow/455/04	2004	Russia	Class II	XXI	XXI.1.2

407	JF824021.1	Avian_orthoavulavirus_1_isolate_Pi/Rus/Moscow/456/04	2004	Russia	Class II	XXI	XXI.1.1
408	JF824022.1	Avian_orthoavulavirus_1_isolate_Pi/Rus/Moscow/515/04	2004	Russia	Class II	XXI	XXI.1.1
409	JF824023.1	Avian_orthoavulavirus_1_isolate_Pi/Rus/Tver/708-4/05	2005	Russia	Class II	XXI	XXI.1.1
410	JF824024.1	Avian_orthoavulavirus_1_isolate_Pi/Rus/Tver/708-5/05	2005	Russia	Class II	XXI	XXI.1.1
411	JF824025.1	Avian_orthoavulavirus_1_isolate_Pi/Rus/Tver/737-2/05	2005	Russia	Class II	XXI	XXI.1.1
412	JF824026.1	Avian_orthoavulavirus_1_isolate_Pi/Rus/Kovrov/0017/09	2009	Russia	Class II	XXI	XXI.1.1
413	JF824027.1	Avian_orthoavulavirus_1_isolate_Pi/Rus/Tula/0529/08	2008	Russia	Class II	XXI	XXI.1.1
414	JF824028.1	Avian_orthoavulavirus_1_isolate_Pi/Rus/Kursk/0484/09	2009	Russia	Class II	XXI	XXI.1.1
415	JF824029.1	Avian_orthoavulavirus_1_isolate_Pi/Rus/Vladimir/495/04	2004	Russia	Class II	XXI	XXI.1.1
416	JF824030.1	Avian_orthoavulavirus_1_isolate_Pi/Rus/Nizhny_Novgorod/668/05	2005	Russia	Class II	XXI	XXI.1.1
417	JF824031.1	Avian_orthoavulavirus_1_isolate_Pi/Rus/Moscow/680/05	2005	Russia	Class II	XXI	XXI.1.2
418	JF824032.1	Avian_orthoavulavirus_1_isolate_Pi/Rus/Vladimir/687/05	2005	Russia	Class II	XXI	XXI.1.1
419	JF824033.1	Avian_orthoavulavirus_1_isolate_Pi/Rus/Tver/701/05	2005	Russia	Class II	XXI	XXI.1.1
420	JF824034.1	Avian_orthoavulavirus_1_isolate_Pi/Rus/Tver/736-1/05	2005	Russia	Class II	XXI	XXI.1.1
421	JF824035.1	Avian_orthoavulavirus_1_isolate_Pi/Rus/Ivanovo/0347/09	2009	Russia	Class II	XXI	XXI.1.1
422	JF824036.1	Avian_orthoavulavirus_1_isolate_Pi/Rus/Tver/708-1/05	2005	Russia	Class II	XXI	XXI.1.1
423	JF824037.1	Avian_orthoavulavirus_1_isolate_Pi/Rus/Tver/708-2/05	2005	Russia	Class II	XXI	XXI.1.1
424	JF824038.1	Avian_orthoavulavirus_1_isolate_Pi/Rus/Tver/738-3/05	2005	Russia	Class II	XXI	XXI.1.1
425	JF824039.1	Avian_orthoavulavirus_1_isolate_Pi/Rus/Yaroslavl/723-1/05	2005	Russia	Class II	XXI	XXI.1.1
426	JF824040.1	Avian_orthoavulavirus_1_isolate_Pi/Rus/Yaroslavl/742-8/05	2005	Russia	Class II	XXI	XXI.1.1
427	JF824041.1	Avian_orthoavulavirus_1_isolate_Pi/Rus/Yaroslavl/743-9/05	2005	Russia	Class II	XXI	XXI.1.1
428	JF824042.1	Avian_orthoavulavirus_1_isolate_Pi/Rus/Yaroslavl/723-2/05	2005	Russia	Class II	XXI	XXI.1.1
429	JF824043.1	Avian_orthoavulavirus_1_isolate_Pi/Rus/Nizhny_Novgorod/0206/06	2006	Russia	Class II	XXI	XXI.1.1
430	JF824044.1	Avian_orthoavulavirus_1_isolate_Pi/Rus/Archangelsk/0858/07	2007	Russia	Class II	XXI	XXI.1.1
431	JF824045.1	Avian_orthoavulavirus_1_isolate_Pi/Rus/Vladimir/0044/08	2008	Russia	Class II	XXI	XXI.1.1
432	JF824046.1	Avian_orthoavulavirus_1_isolate_Pi/Rus/Moscow/0207/06	2006	Russia	Class II	XXI	XXI.1.1

433	JF824047.1	Avian_orthoavulavirus_1_isolate_Pi/Rus/Kostroma/0843/07	2007	Russia	Class II	XXI	XXI.1.1
434	JF824048.1	Avian_orthoavulavirus_1_isolate_Pi/Rus/Arhangelsk/0002/09	2009	Russia	Class II	XXI	XXI.1.1
435	JF824049.1	Avian_orthoavulavirus_1_isolate_Pi/Rus/Arhangelsk/0003/09	2009	Russia	Class II	XXI	XXI.1.1
436	JF824050.1	Avian_orthoavulavirus_1_isolate_Pi/Rus/Kemerovo/0005/09	2009	Russia	Class II	XXI	XXI.1.1
437	JF824051.1	Avian_orthoavulavirus_1_isolate_Pi/Rus/Vologda/0016/09	2009	Russia	Class II	XXI	XXI.1.1
438	JF824052.1	Avian_orthoavulavirus_1_isolate_Pi/Rus/Tver/0067/08	2008	Russia	Class II	XXI	XXI.1.1
439	JF824053.1	Avian_orthoavulavirus_1_isolate_Pi/Rus/Tver/0068/08	2008	Russia	Class II	XXI	XXI.1.1
440	JF824054.1	Avian_orthoavulavirus_1_isolate_Pi/Rus/Tver/0069/08	2008	Russia	Class II	XXI	XXI.1.1
441	JF824055.1	Avian_orthoavulavirus_1_isolate_Pi/Rus/Komi-Uhta/0070/08	2008	Russia	Class II	XXI	XXI.1.1
442	JF824056.1	Avian_orthoavulavirus_1_isolate_Pi/Rus/Komi_Sosnogorsk/0071/08	2008	Russia	Class II	XXI	XXI.1.1
443	JF824057.1	Avian_orthoavulavirus_1_isolate_Pi/Rus/Komi_Sosnogorsk/0072/08	2008	Russia	Class II	XXI	XXI.1.1
444	JF824058.1	Avian_orthoavulavirus_1_isolate_Pi/Rus/Komi/0073/08	2008	Russia	Class II	XXI	XXI.1.1
445	JF824059.1	Avian_orthoavulavirus_1_isolate_Pi/Rus/Komi/0081/08	2008	Russia	Class II	XXI	XXI.1.1
446	JF824060.1	Avian_orthoavulavirus_1_isolate_Pi/Rus/Leningrad/0258/09	2009	Russia	Class II	XXI	XXI.1.1
447	JF824061.1	Avian_orthoavulavirus_1_isolate_Pi/Rus/Tula/0296/09	2009	Russia	Class II	XXI	XXI.1.1
448	JF824062.1	Avian_orthoavulavirus_1_isolate_Pi/Rus/Kostroma/0318/08	2008	Russia	Class II	XXI	XXI.1.1
449	JF824063.1	Avian_orthoavulavirus_1_isolate_Pi/Rus/Ivanovo/0348/09	2009	Russia	Class II	XXI	XXI.1.1
450	JF824064.1	Avian_orthoavulavirus_1_isolate_Pi/Rus/Moscow/0453/09	2009	Russia	Class II	XXI	XXI.1.1
451	JF824065.1	Avian_orthoavulavirus_1_isolate_Pi/Rus/Kursk/0485/09	2009	Russia	Class II	XXI	XXI.1.1
452	JF824066.1	Avian_orthoavulavirus_1_isolate_Pi/Rus/Tula/0500/08	2008	Russia	Class II	XXI	XXI.1.1
453	JF824067.1	Avian_orthoavulavirus_1_isolate_Pi/Rus/Tver/1600/08	2008	Russia	Class II	XXI	XXI.1.1
454	JF824068.1	Avian_orthoavulavirus_1_isolate_Pi/Rus/Tver/1601/08	2008	Russia	Class II	XXI	XXI.1.1
455	JF827027.1	Newcastle_disease_virus_isolate_Pi/Rus/Kemerovo/0267/09	2009	Russia	Class II	XXI	XXI.1.1
456	JN638234.1	Newcastle_disease_virus_strain_NDV/DOVE/IT/11RS98_102VIR/2011	2011	Italy	Class II	XXI	XXI.2
457	JN638235.1	Newcastle_disease_virus_strain_NDV/DOVE/IT/11RS100_104VIR/2011	2011	Italy	Class II	XXI	XXI.2
458	JN638236.1	Newcastle_disease_virus_strain_NDV/DOVE/IT/10RS6171_7154VIR/2010	2010	Italy	Class II	XXI	XXI.2

459	JN638237.1	Newcastle_disease_virus_strain_NDV/DOVE/IT/10RS6431_RS7313VIR/2010	2010	Italy	Class II	VI	VI.2.1.1.2.1
460	JN806235.1	Newcastle_disease_virus_strain_APMV-1/pigeon/Almaty/1063/05	2005	Kazakhstan	Class II	XXI	XXI.1.1
461	JN806236.1	Newcastle_disease_virus_strain_APMV-1/pigeon/Almaty/1064/05	2005	Kazakhstan	Class II	XXI	XXI.1.1
462	JN806237.1	Newcastle_disease_virus_strain_APMV-1/pigeon/Almaty/1065/05	2005	Kazakhstan	Class II	XXI	XXI.1.1
463	JN872160.1	Pigeon_paramyxovirus_1_isolate_Pigeon/Minnesota/511296/07	2007	USA	Class II	VI	VI.2.1.1.1
464	JN872166.1	Pigeon_paramyxovirus_1_isolate_Dove/Italy/VIR_24/2008	2008	Italy	Class II	VI	VI.2.1.1.2.1
465	JN872167.1	Pigeon_paramyxovirus_1_isolate_Dove/FL/455682-2/2006	2006	USA	Class II	VI	VI.2.1.1.1
466	JN872170.1	Pigeon_paramyxovirus_1_isolate_Pigeon/Texas/5254-12/2010	2010	USA	Class II	VI	VI.2.1.1.1
467	JN872172.1	Pigeon_paramyxovirus_1_isolate_Raptor/Minnesota/18575-4/2009	2009	USA	Class II	VI	VI.2.1.1.1
468	JN872173.1	Pigeon_paramyxovirus_1_isolate_Pigeon/New_York/44407/1984	1984	USA	Class II	VI	VI.1
469	JN872174.1	Pigeon_paramyxovirus_1_isolate_New_Jersey/9564/2010	2010	USA	Class II	VI	VI.2.1.1.1
470	JN872175.1	Pigeon_paramyxovirus_1_isolate_Pigeon/Minnesota/723/2009	2009	USA	Class II	VI	VI.2.1.1.1
471	JN872176.1	Pigeon_paramyxovirus_1_isolate_Pheasant/Massachusetts/359425/2005	2005	USA	Class II	VI	VI.2.1.1.1
472	JN872178.1	Pigeon_paramyxovirus_1_isolate_Pigeon/Louisiana/331309/2004	2004	USA	Class II	VI	VI.2.1.1.1
473	JN872179.1	Pigeon_paramyxovirus_1_isolate_Pigeon/Nevada/241851/2003	2003	USA	Class II	VI	VI.2.1.1.1
474	JN872180.1	Pigeon_paramyxovirus_1_isolate_Pigeon/Texas/209682/2002	2002	USA	Class II	VI	VI.2.2.1
475	JN872182.1	Pigeon_paramyxovirus_1_isolate_Pigeon/New_York/12339/1998	1998	USA	Class II	VI	VI.2.2.1
476	JN872183.1	Pigeon_paramyxovirus_1_isolate_Pigeon/Maryl/2075/1998	1998	USA	Class II	VI	VI.2.1.1.1
477	JN872184.1	Pigeon_paramyxovirus_1_isolate_Pigeon/Wyoming/20210/1997	1997	USA	Class II	VI	VI.2.2.1
478	JN872185.1	Pigeon_paramyxovirus_1_isolate_Pigeon/New_York/32851-2/1995	1995	USA	Class II	VI	VI.2.2.1
479	JN872186.1	Pigeon_paramyxovirus_1_isolate_Pigeon/Indiana/18002/1991	1991	USA	Class II	VI	VI.2.2.1
480	JN872187.1	Pigeon_paramyxovirus_1_isolate_Pigeon/Illinois/11748/1993	1993	USA	Class II	VI	VI.2.2.1
481	JN872188.1	Pigeon_paramyxovirus_1_isolate_Pigeon/Minnesota/2446/1989	1989	USA	Class II	VI	VI.2.2.1
482	JN872190.1	Pigeon_paramyxovirus_1_isolate_Pigeon/South_Dakota/486839/2007	2007	USA	Class II	VI	VI.2.1.1.1
483	JN941994.1	Pigeon_paramyxovirus_1_isolate_Pigeon/Michigan/1924/2008	2008	USA	Class II	VI	VI.2.1.1.1
484	JN941995.1	Pigeon_paramyxovirus_1_isolate_Pigeon/New_York/1989-19/2008	2008	USA	Class II	VI	VI.2.1.1.2.1

485	JN941996.1	Pigeon_paramyxovirus_1_isolate_Pigeon/Pennsylvania/2062/2008	2008	USA	Class II	VI	VI.2.1.1.1
486	JN941997.1	Pigeon_paramyxovirus_1_isolate_Pigeon/Florida/2181-43/2008	2008	USA	Class II	VI	VI.2.1.1.2.1
487	JN941998.1	Pigeon_paramyxovirus_1_isolate_Pigeon/Pennsylvania/2282/2008	2008	USA	Class II	VI	VI.2.1.1.1
488	JN941999.1	Pigeon_paramyxovirus_1_isolate_Pigeon/Minnesota/77/2008	2008	USA	Class II	VI	VI.2.1.1.1
489	JN942000.1	Pigeon_paramyxovirus_1_isolate_Dove/Florida/1287/2008	2008	USA	Class II	VI	VI.2.1.1.1
490	JN942095.1	Pigeon_paramyxovirus_1_isolate_Pigeon/Oklahoma/7668/1997	1997	USA	Class II	VI	VI.2.2.1
491	JN942098.1	Pigeon_paramyxovirus_1_isolate_Pigeon/North_Carolina/56404/2000	2000	USA	Class II	VI	VI.2.2.1
492	JN967786.1	Pigeon_paramyxovirus_1_isolate_Pigeon/New_Jersey/58300-4/2000	2000	USA	Class II	VI	VI.2.2.1
493	JN967787.1	Pigeon_paramyxovirus_1_isolate_Pigeon/Illinois/37397/1987	1987	USA	Class II	VI	VI.1
494	JN967788.1	Pigeon_paramyxovirus_1_isolate_Pigeon/Maryl/11936/1985	1985	USA	Class II	VI	VI.1
495	JN986839.1	Newcastle_disease_virus_strain_PPMV-1/pigeon/IE/806/04	2004	Ireland	Class II	VI	VI.2.1.1.2.2
496	JQ039385.1	Newcastle_disease_virus_strain_dove/Nigeria/VRD07-163/2007	2007	Nigeria	Class II	XXI	XXI.1.1
497	JQ039387.1	Newcastle_disease_virus_strain_pigeon/Nigeria/VRD08-37BRpe(7-9)/2008	2008	Nigeria	Class II	VI	VI.2.1.2
498	JQ039388.1	Newcastle_disease_virus_strain_pigeon/Nigeria/VRD08-37(10-11-13)/2008	2008	Nigeria	Class II	VI	VI.2.1.2
499	JQ039389.1	Newcastle_disease_virus_strain_pigeon/Nigeria/VRD07-369/2007	2007	Nigeria	Class II	XXI	XXI.1.1
500	JQ039391.1	Newcastle_disease_virus_strain_pigeon/Nigeria/VRD07-231/2007	2007	Nigeria	Class II	VI	VI.2.1.2
501	JQ039395.1	Newcastle_disease_virus_strain_pigeon/Nigeria/VRD07-173/2007	2007	Nigeria	Class II	XXI	XXI.1.1
502	JQ268609.1	Newcastle_disease_virus_strain_Miangyang12505	2011	China	Class II	VI	VI.2.1.1.2.1
503	JQ290284.1	Pigeon_paramyxovirus_1_strain_P/Anhui/1/09	2009	China	Class II	VI	VI.2.2.2
504	JQ779825.1	Newcastle_disease_virus_isolate_08RS-491	2008	Italy	Class II	VI	VI.2.1.1.2.1
505	JQ979176.1	Newcastle_disease_virus_strain_pigeon/China/SDLC/2011	2011	China	Class II	VI	VI.2.1.1.2.2
506	JQ993431.1	Newcastle_disease_virus_strain_pigeon/China/SDS/2011	2011	China	Class II	VI	VI.2.1.1.2.2
507	JX028551.1	Newcastle_disease_virus_isolate_BD-C162/10	2010	Bangladesh	Class II	XIII	XIII.2.2
508	JX028552.2	Avian_orthoavulavirus_1_isolate_BD-P01/10	2010	Bangladesh	Class II	XXI	XXI.1.2
509	JX094510.1	Newcastle_disease_virus_strain_sms12	2012	China	Class II	VI	VI.2.1.1.2.1
510	JX244789.1	Newcastle_disease_virus_isolate_Pigeon/China/JM/11/00	2000	China	Class II	VI	VI.2.1.1.2.1

511	JX244790.1	Newcastle_disease_virus_isolate_Pigeon/China/ZQ/17/00	2000	China	Class II	VII	VII.1.1
512	JX244791.1	Newcastle_disease_virus_isolate_Pigeon/China/SD/54/06	2006	China	Class II	VII	VII.1.1
513	JX244792.1	Newcastle_disease_virus_isolate_Pigeon/China/SD/55/06	2006	China	Class II	I	I.1.1
514	JX244793.1	Newcastle_disease_virus_isolate_Pigeon/China/93/08	2008	China	Class II	II	II
515	JX244794.1	Newcastle_disease_virus_isolate_Pigeon/China/100/08	2008	China	Class II	VI	VI.2.2.2
516	JX244795.1	Newcastle_disease_virus_isolate_Pigeon/China/101/08	2008	China	Class II	II	II
517	JX244796.1	Newcastle_disease_virus_isolate_Pigeon/China/102/08	2008	China	Class II	II	II
518	JX244797.1	Newcastle_disease_virus_isolate_Pigeon/China/103/08	2008	China	Class II	II	II
519	JX244798.1	Newcastle_disease_virus_isolate_Pigeon/China/104/08	2008	China	Class II	VI	VI.2.1.1.2.1
520	JX244799.1	Newcastle_disease_virus_isolate_Pigeon/China/105/08	2008	China	Class II	VI	VI.2.1.1.2.1
521	JX244800.1	Newcastle_disease_virus_isolate_Pigeon/China/106/08	2008	China	Class II	VI	VI.2.1.1.2.1
522	JX244801.1	Newcastle_disease_virus_isolate_Pigeon/China/107/08	2008	China	Class II	VI	VI.2.2.2
523	JX244802.1	Newcastle_disease_virus_isolate_Pigeon/China/109/08	2008	China	Class II	II	II
524	JX244803.1	Newcastle_disease_virus_isolate_Pigeon/China/110/08	2008	China	Class II	VI	VI.2.2.2
525	JX244804.1	Newcastle_disease_virus_isolate_Pigeon/China/111/08	2008	China	Class II	II	II
526	JX244805.1	Newcastle_disease_virus_isolate_Pigeon/China/112/08	2008	China	Class II	VI	VI.2.2.2
527	JX244806.1	Newcastle_disease_virus_isolate_Pigeon/China/113/08	2008	China	Class II	VII	VII.1.1
528	JX244807.1	Newcastle_disease_virus_isolate_Pigeon/China/KP/114/08	2008	China	Class II	II	II
529	JX486550.1	Pigeon_paramyxovirus_1_strain_pi/CH/LGD/110208	2011	China	Class II	VI	VI.2.1.1.2.1
530	JX486551.1	Pigeon_paramyxovirus_1_strain_pi/CH/LJL/100605	2010	China	Class II	VI	VI.2.1.1.2.2
531	JX486552.1	Pigeon_paramyxovirus_1_strain_pi/CH/LLN/110713	2011	China	Class II	VI	VI.2.1.1.2.2
532	JX486553.1	Pigeon_paramyxovirus_1_strain_pi/CH/LHLJ/110813	2011	China	Class II	VI	VI.2.1.1.2.1
533	JX486554.1	Pigeon_paramyxovirus_1_strain_pi/CH/LHLJ/110822	2011	China	Class II	VI	VI.2.1.1.2.1
534	JX486555.1	Pigeon_paramyxovirus_1_strain_pi/CH/LGD/110945	2011	China	Class II	VI	VI.2.1.1.2.1
535	JX486556.1	Pigeon_paramyxovirus_1_strain_pi/CH/LGD/110947	2011	China	Class II	VI	VI.2.1.1.2.1
536	JX486557.1	Pigeon_paramyxovirus_1_strain_pi/CH/LJL/120404	2012	China	Class II	VI	VI.2.1.1.2.1

537	JX518532.1	Pigeon_paramyxovirus_1	2012	Kenya	Class II	VI	VI.2.1.2
538	JX844034.1	Newcastle_disease_virus_strain_NDV/pigeon/Finl/6985/2008	2008	Finland	Class II	I	I.1.1
539	JX844035.1	Newcastle_disease_virus_strain_NDV/pigeon/Finl/17557/2008	2008	Finland	Class II	I	I.1.1
540	JX844036.1	Newcastle_disease_virus_strain_NDV/pigeon/Finl/15475/2009	2009	Finland	Class II	VI	VI.2.1.1.2.1
541	JX855039.1	Newcastle_disease_virus_isolate_pigeon/China/Shaanxi/g3/2003	2003	China	Class II	II	II
542	JX855040.1	Newcastle_disease_virus_isolate_pigeon/China/Shaanxi/g10/2006	2006	China	Class II	XX	XX
543	JX901109.1	Pigeon_paramyxovirus_1_strain_PPMV-1/Belgium/98-238/1998	1998	Belgium	Class II	VI	VI.2.1.1.2.1
544	JX901111.1	Pigeon_paramyxovirus_1_strain_PPMV-1/Belgium/98-321/1998	1998	Belgium	Class II	VI	VI.2.1.1.2.1
545	JX901119.1	Pigeon_paramyxovirus_1_strain_PPMV-1/Belgium/05-01824/2005	2005	Belgium	Class II	VI	VI.2.1.1.2.1
546	JX901120.1	Pigeon_paramyxovirus_1_strain_PPMV-1/Belgium/05-03936-8/2005	2005	Belgium	Class II	VI	VI.2.1.1.2.1
547	JX901121.1	Pigeon_paramyxovirus_1_strain_PPMV-1/Belgium/07-04943/2007	2007	Belgium	Class II	VI	VI.2.1.1.2.2
548	JX901122.1	Pigeon_paramyxovirus_1_strain_PPMV-1/Belgium/11-07574/2011	2011	Belgium	Class II	VI	VI.2.1.1.2.2
549	JX901123.1	Pigeon_paramyxovirus_1_strain_PPMV-1/Belgium/11-08304/2011	2011	Belgium	Class II	VI	VI.2.1.1.2.2
550	JX901124.1	Pigeon_paramyxovirus_1_strain_PPMV-1/Belgium/11-09620/2011	2011	Belgium	Class II	VI	VI.2.1.1.2.2
551	JX901312.1	Newcastle_disease_virus_isolate_APMV1/Pigeon/USA/0101/2001	2001	USA	Class II	VI	VI.2.2.1
552	JX901313.1	Newcastle_disease_virus_isolate_APMV1/Pigeon/USA/0102/2001	2001	USA	Class II	VI	VI.2.1.1.1
553	JX901314.1	Newcastle_disease_virus_isolate_APMV1/Dove/USA/0103/2001	2001	USA	Class II	VI	VI.2.1.1.1
554	JX901315.1	Newcastle_disease_virus_isolate_APMV1/Pigeon/USA/0106/2001	2001	USA	Class II	VI	VI.2.1.1.1
555	JX901317.1	Newcastle_disease_virus_isolate_APMV1/Pigeon/USA/0201/2002	2002	USA	Class II	VI	VI.2.1.1.1
556	JX901319.1	Newcastle_disease_virus_isolate_APMV1/Pigeon/PA/USA/0501/2005	2005	USA	Class II	VI	VI.2.1.1.1
557	JX901320.1	Newcastle_disease_virus_isolate_APMV1/Pigeon/PA/USA/0502/2005	2005	USA	Class II	VI	VI.2.1.1.1
558	JX901323.1	Newcastle_disease_virus_isolate_APMV1/Pigeon/PA/USA/0601/2006	2006	USA	Class II	VI	VI.2.1.1.1
559	JX901324.1	Newcastle_disease_virus_isolate_APMV1/Pigeon/PA/USA/0602/2006	2006	USA	Class II	VI	VI.2.1.1.1
560	JX901325.1	Newcastle_disease_virus_isolate_APMV1/Pigeon/NY/USA/0603/2006	2006	USA	Class II	VI	VI.2.1.1.1
561	JX901326.1	Newcastle_disease_virus_isolate_APMV1/Pigeon/NJ/USA/0604/2006	2006	USA	Class II	VI	VI.2.1.1.1
562	JX901327.1	Newcastle_disease_virus_isolate_APMV1/Pigeon/PA/USA/0605/2006	2006	USA	Class II	VI	VI.2.1.1.1

563	JX901328.1	Newcastle_disease_virus_isolate_APMV1/Pigeon/NJ/USA/0607/2006	2006	USA	Class II	VI	VI.2.1.1.1
564	JX901329.1	Newcastle_disease_virus_isolate_APMV1/Pigeon/PA/USA/0701/2007	2007	USA	Class II	VI	VI.2.1.1.1
565	JX901330.1	Newcastle_disease_virus_isolate_APMV1/Pigeon/PA/USA/0702/2007	2007	USA	Class II	VI	VI.2.1.1.1
566	JX901331.1	Newcastle_disease_virus_isolate_APMV1/Pigeon/PA/USA/0702.2/2007	2007	USA	Class II	II	II
567	JX901332.1	Newcastle_disease_virus_isolate_APMV1/Pigeon/MO/USA/0703/2007	2007	USA	Class II	VI	VI.2.1.1.1
568	JX901333.1	Newcastle_disease_virus_isolate_APMV1/Pigeon/PA/USA/0704/2007	2007	USA	Class II	VI	VI.2.1.1.1
569	JX901334.1	Newcastle_disease_virus_isolate_APMV1/Pigeon/PA/USA/0705/2007	2007	USA	Class II	VI	VI.2.1.1.1
570	JX901335.1	Newcastle_disease_virus_isolate_APMV1/Pigeon/PA/USA/0706/2007	2007	USA	Class II	VI	VI.2.1.1.1
571	JX901336.1	Newcastle_disease_virus_isolate_APMV1/Pigeon/NC/USA/0707/2007	2007	USA	Class II	VI	VI.2.1.1.1
572	JX901337.1	Newcastle_disease_virus_isolate_APMV1/Pigeon/CT/USA/0708/2007	2007	USA	Class II	VI	VI.2.1.1.1
573	JX901338.1	Newcastle_disease_virus_isolate_APMV1/Pigeon/OH/USA/0709/2007	2007	USA	Class II	VI	VI.2.1.1.1
574	JX901339.1	Newcastle_disease_virus_isolate_APMV1/Pigeon/NJ/USA/0710/2007	2007	USA	Class II	VI	VI.2.1.1.1
575	JX901340.1	Newcastle_disease_virus_isolate_APMV1/Pigeon/PA/USA/0711/2007	2007	USA	Class II	VI	VI.2.1.1.1
576	JX901341.1	Newcastle_disease_virus_isolate_APMV1/Pigeon/PA/USA/0712/2007	2007	USA	Class II	VI	VI.2.2.1
577	JX901342.1	Newcastle_disease_virus_isolate_APMV1/Pigeon/MN/USA/0713/2007	2007	USA	Class II	VI	VI.2.1.1.1
578	JX901343.1	Newcastle_disease_virus_isolate_APMV1/Pigeon/MN/USA/0713.2/2007	2007	USA	Class II	II	II
579	JX901344.1	Newcastle_disease_virus_isolate_APMV1/Pigeon/ME/USA/0714/2007	2007	USA	Class II	VI	VI.2.1.1.1
580	JX901345.1	Newcastle_disease_virus_isolate_APMV1/Pigeon/MD/USA/0715/2007	2007	USA	Class II	II	II
581	JX901346.1	Newcastle_disease_virus_isolate_APMV1/Pigeon/PA/USA/0716/2007	2007	USA	Class II	VI	VI.2.1.1.1
582	JX901347.1	Newcastle_disease_virus_isolate_APMV1/Pigeon/NY/USA/0717/2007	2007	USA	Class II	VI	VI.2.1.1.1
583	JX901348.1	Newcastle_disease_virus_isolate_APMV1/Pigeon/PA/USA/0718/2007	2007	USA	Class II	VI	VI.2.1.1.1
584	JX901349.1	Newcastle_disease_virus_isolate_APMV1/Pigeon/MD/USA/0719/2007	2007	USA	Class II	VI	VI.2.1.1.1
585	JX901350.1	Newcastle_disease_virus_isolate_APMV1/Pigeon/PA/USA/0720/2007	2007	USA	Class II	VI	VI.2.1.1.1
586	JX901351.1	Newcastle_disease_virus_isolate_APMV1/Pigeon/NJ/USA/0721/2007	2007	USA	Class II	VI	VI.2.1.1.1
587	JX901352.1	Newcastle_disease_virus_isolate_APMV1/Pigeon/PA/USA/0723/2007	2007	USA	Class II	VI	VI.2.1.1.1
588	JX901353.1	Newcastle_disease_virus_isolate_APMV1/Pigeon/PA/USA/0724/2007	2007	USA	Class II	VI	VI.2.1.1.1

589	JX901354.1	Newcastle_disease_virus_isolate_APMV1/Pigeon/PA/USA/0725/2007	2007	USA	Class II	VI	VI.2.1.1.1
590	JX901355.1	Newcastle_disease_virus_isolate_APMV1/Pigeon/PA/USA/0726/2007	2007	USA	Class II	VI	VI.2.1.1.1
591	JX901356.1	Newcastle_disease_virus_isolate_APMV1/Pigeon/PA/USA/0727/2007	2007	USA	Class II	VI	VI.2.1.1.1
592	JX901357.1	Newcastle_disease_virus_isolate_APMV1/Pigeon/PA/USA/0728/2007	2007	USA	Class II	VI	VI.2.1.1.1
593	JX901358.1	Newcastle_disease_virus_isolate_APMV1/Pigeon/PA/USA/0801/2008	2008	USA	Class II	VI	VI.2.1.1.1
594	JX901359.1	Newcastle_disease_virus_isolate_APMV1/Pigeon/PA/USA/0802/2008	2008	USA	Class II	VI	VI.2.1.1.1
595	JX901360.1	Newcastle_disease_virus_isolate_APMV1/Pigeon/PA/USA/0803/2008	2008	USA	Class II	VI	VI.2.1.1.1
596	JX901361.1	Newcastle_disease_virus_isolate_APMV1/Pigeon/PA/USA/0804/2008	2008	USA	Class II	VI	VI.2.1.1.1
597	JX901362.1	Newcastle_disease_virus_isolate_APMV1/Pigeon/PA/USA/0805/2008	2008	USA	Class II	VI	VI.2.1.1.1
598	JX901363.1	Newcastle_disease_virus_isolate_APMV1/Pigeon/PA/USA/0806/2008	2008	USA	Class II	VI	VI.2.1.1.1
599	JX901364.1	Newcastle_disease_virus_isolate_APMV1/Pigeon/PA/USA/0807/2008	2008	USA	Class II	VI	VI.2.1.1.1
600	JX901365.1	Newcastle_disease_virus_isolate_APMV1/Pigeon/PA/USA/0808/2008	2008	USA	Class II	VI	VI.2.1.1.1
601	JX901366.1	Newcastle_disease_virus_isolate_APMV1/Pigeon/PA/USA/0809/2008	2008	USA	Class II	VI	VI.2.1.1.1
602	JX901367.1	Newcastle_disease_virus_isolate_APMV1/Pigeon/PA/USA/0810/2008	2008	USA	Class II	VI	VI.2.1.1.1
603	JX901368.1	Newcastle_disease_virus_isolate_APMV1/Pigeon/PA/USA/0811/2008	2008	USA	Class II	VI	VI.2.1.1.1
604	JX901369.1	Newcastle_disease_virus_isolate_APMV1/Pigeon/NJ/USA/0812/2008	2008	USA	Class II	VI	VI.2.1.1.1
605	JX901373.1	Newcastle_disease_virus_isolate_APMV1/Pigeon/PA/USA/0902/2009	2009	USA	Class II	VI	VI.2.1.1.1
606	JX901374.1	Newcastle_disease_virus_isolate_APMV1/Pigeon/OH/USA/0903/2009	2009	USA	Class II	VI	VI.2.1.1.1
607	JX901375.1	Newcastle_disease_virus_isolate_APMV1/Pigeon/PA/USA/0904/2009	2009	USA	Class II	VI	VI.2.1.1.1
608	JX901376.1	Newcastle_disease_virus_isolate_APMV1/Pigeon/PA/USA/0905/2009	2009	USA	Class II	VI	VI.2.1.1.1
609	JX901377.1	Newcastle_disease_virus_isolate_APMV1/Pigeon/PA/USA/0906/2009	2009	USA	Class II	VI	VI.2.1.1.1
610	KC143154.1	Newcastle_disease_virus_isolate_Ch98-1	1998	China	Class II	VI	VI.2.2.2
611	KC143156.1	Newcastle_disease_virus_isolate_NDV00-044	2000	China	Class II	VI	VI.2.2.2
612	KC143157.1	Newcastle_disease_virus_isolate_NDV02-044	2002	China	Class II	VI	VI.2.2.2
613	KC143158.1	Newcastle_disease_virus_isolate_NDV03-051	2003	China	Class II	VI	VI.2.2.2
614	KC143159.1	Newcastle_disease_virus_isolate_NDV05-027	2005	China	Class II	VI	VI.2.1.1.2.1

615	KC143160.1	Newcastle_disease_virus_isolate_NDV05-028	2005	China	Class II	VI	VI.2.2.2
616	KC143161.1	Newcastle_disease_virus_isolate_NDV05-029	2005	China	Class II	VI	VI.2.2.2
617	KC143164.1	Newcastle_disease_virus_isolate_SH215	2011	China	Class II	VI	VI.2.1.1.2.2
618	KC424430.1	Newcastle_disease_virus_isolate_spotted_dove/China/08	2008	China	Class II	IX	IX
619	KC568204.1	Avian_orthoavulavirus_1_isolate_pigeon/Nigeria/ZM/KN/PG01/N1/688/2009	2009	Nigeria	Class II	XVII	XVII
620	KC568210.1	Newcastle_disease_virus_isolate_NG-689/KT.MSH.15C	2009	Nigeria	Class II	XIV	XIV.2
621	KC568228.1	Newcastle_disease_virus_isolate_NG-713/YB.GSH1.4-6T	2009	Nigeria	Class II	XIV	XIV.2
622	KC915205.1	Pigeon_paramyxovirus_1_strain_NDV/pigeon/Macedonia/234/2007	2007	Macedonia	Class II	VI	VI.2.1.1.2.2
623	KC915206.1	Pigeon_paramyxovirus_1_strain_NDV/pigeon/Macedonia/1501/2007	2007	Macedonia	Class II	VI	VI.2.1.1.2.2
624	KC915207.1	Pigeon_paramyxovirus_1_strain_NDV/pigeon/Macedonia/1503/2007	2007	Macedonia	Class II	VI	VI.2.1.1.2.2
625	KC915208.1	Pigeon_paramyxovirus_1_strain_NDV/pigeon/Macedonia/230/2008	2008	Macedonia	Class II	VI	VI.2.1.1.2.2
626	KC915209.1	Pigeon_paramyxovirus_1_strain_NDV/pigeon/Macedonia/232/2008	2008	Macedonia	Class II	VI	VI.2.1.1.2.2
627	KC915210.1	Pigeon_paramyxovirus_1_strain_NDV/pigeon/Macedonia/962/2008	2008	Macedonia	Class II	VI	VI.2.1.1.2.2
628	KC915212.1	Pigeon_paramyxovirus_1_strain_NDV/pigeon/Macedonia/2810/2010	2010	Macedonia	Class II	VI	VI.2.1.1.2.2
629	KC915213.1	Pigeon_paramyxovirus_1_strain_NDV/pigeon/Macedonia/233/2011	2011	Macedonia	Class II	VI	VI.2.1.1.2.2
630	KC915214.1	Pigeon_paramyxovirus_1_strain_NDV/pigeon/Macedonia/415/2011	2011	Macedonia	Class II	VI	VI.2.1.1.2.2
631	KC915215.1	Pigeon_paramyxovirus_1_strain_NDV/pigeon/Macedonia/419/2011	2011	Macedonia	Class II	VI	VI.2.1.1.2.2
632	KC915216.1	Pigeon_paramyxovirus_1_strain_NDV/feral_pigeon/Macedonia/9701/2012	2012	Macedonia	Class II	VI	VI.2.1.1.2.2
633	KC934170.1	Newcastle_disease_virus_strain_SpottedDove/China/08	2008	China	Class II	IX	IX
634	KF828884.1	Newcastle_disease_virus_isolate_BJP13	2013	China	Class II	VI	VI.2.1.1.2.2
635	KF944359.1	Newcastle_disease_virus_strain_pigeon/IS/854/2013	2013	Israel	Class II	VI	VI.2.1.2
636	KJ136261.1	Newcastle_disease_virus_isolate_JLDH_2013	2013	China	Class II	VII	VII.1.1
637	KJ473708.1	Pigeon_paramyxovirus_1_strain_Eurasian_collared_dove/Arizona/145909/2009	2009	USA	Class II	VI	VI.2.1.1.1
638	KJ473709.1	Pigeon_paramyxovirus_1_strain_Eurasian_collared_dove/Montana/146350/2009	2009	USA	Class II	VI	VI.2.1.1.1
639	KJ473710.1	Pigeon_paramyxovirus_1_strain_Eurasian_collared_dove/Montana/146820/2010	2010	USA	Class II	VI	VI.2.1.1.1
640	KJ525672.1	Newcastle_disease_virus_isolate_Pi/SD/JN/2010	2010	China	Class II	VI	VI.2.1.1.2.1

641	KJ525673.1	Newcastle_disease_virus_isolate_Pi/SD/JN/2011	2011	China	Class II	VI	VI.2.1.1.2.1
642	KJ525674.1	Newcastle_disease_virus_isolate_Pi/SX/P1/2013	2013	China	Class II	VI	VI.2.1.1.2.1
643	KJ525675.1	Newcastle_disease_virus_isolate_Pi/SX/P2/2013	2013	China	Class II	VI	VI.2.1.1.2.1
644	KJ600777.1	Newcastle_disease_virus_isolate_Pi/China/SD/2012/132	2012	China	Class II	VI	VI.2.1.1.2.1
645	KJ600778.1	Newcastle_disease_virus_isolate_Pi/China/SH/2013/163	2013	China	Class II	VI	VI.2.1.1.2.2
646	KJ600779.1	Newcastle_disease_virus_isolate_Pi/China/SH/2013/167	2013	China	Class II	VI	VI.2.1.1.2.2
647	KJ600780.1	Newcastle_disease_virus_isolate_Pi/China/SH/2013/168	2013	China	Class II	VI	VI.2.1.1.2.2
648	KJ607163.1	Pigeon_paramyxovirus_1_strain_pi/CH/LJS/1/04	2004	China	Class II	VI	VI.2.2.2
649	KJ607164.1	Pigeon_paramyxovirus_1_strain_pi/CH/LJS/1/03	2003	China	Class II	VI	VI.2.2.2
650	KJ607165.1	Pigeon_paramyxovirus_1_strain_pi/CH/LHLJ/131237	2013	China	Class II	VI	VI.2.1.1.2.2
651	KJ607166.1	Pigeon_paramyxovirus_1_strain_pi/CH/LHLJ/131011	2013	China	Class II	VI	VI.2.1.1.2.2
652	KJ736742.1	Newcastle_disease_virus_isolate_R75/98	1998	Germany	Class II	VI	VI.2.1.1.1
653	KJ782376.1	Pigeon_paramyxovirus_1_strain_pi/CH/NM/0707/2011	2011	China	Class II	VI	VI.2.1.1.2.2
654	KJ808819.1	Newcastle_disease_virus_isolate_Pigeon/China/BJ2013	2013	China	Class II	VI	VI.2.1.1.2.2
655	KJ808820.1	Newcastle_disease_virus_isolate_Pigeon/China/SD2012	2012	China	Class II	VI	VI.2.2.2
656	KJ914671.1	Avian_orthoavulavirus_1_isolate_pigeon/Ukraine/Dnipropetrovsk/1-18-11/2011	2011	Ukraine	Class II	XXI	XXI.1.1
657	KJ914672.1	Avian_orthoavulavirus_1_isolate_pigeon/Ukraine/Ukromne/3-26-11/2011	2011	Ukraine	Class II	XXI	XXI.1.1
658	KJ920204.1	Newcastle_disease_virus_isolate_NDV/Altai/pigeon/770/2011	2011	Russia	Class II	XXI	XXI.1.1
659	KM374056.1	Newcastle_disease_virus_strain_pigeon/Yunnan/1111/2013	2013	China	Class II	VI	VI.2.1.1.2.2
660	KM374057.1	Newcastle_disease_virus_strain_pigeon/Anhui/2365/2012	2012	China	Class II	VI	VI.2.1.1.2.2
661	KM374058.1	Newcastle_disease_virus_strain_pigeon/Anhui/2369/2012	2012	China	Class II	VI	VI.2.1.1.2.2
662	KM374059.1	Newcastle_disease_virus_strain_pigeon/Guangxi/1015/2013	2013	China	Class II	VI	VI.2.1.1.2.1
663	KM374060.1	Newcastle_disease_virus_strain_pigeon/Shanghai/215/2011	2011	China	Class II	VI	VI.2.1.1.2.2
664	KM374061.1	Newcastle_disease_virus_strain_pigeon/Zhejiang/2036/2012	2012	China	Class II	VI	VI.2.1.1.2.2
665	KM669999.1	Newcastle_disease_virus_strain_SD02/14	2014	China	Class II	VI	VI.2.1.1.2.2
666	KP780870.1	Avian_orthoavulavirus_1_isolate_ND0002270	2013	USA	Class II	VI	VI.2.1.1.1

667	KP780871.1	Avian_orthoavulavirus_1_isolate_ND0007190	2013	USA	Class II	VI	VI.2.1.1.1
668	KP780872.1	Avian_orthoavulavirus_1_isolate_ND0007186	2013	USA	Class II	VI	VI.2.1.1.1
669	KP780873.1	Avian_orthoavulavirus_1_isolate_ND0007187	2013	USA	Class II	VI	VI.2.1.1.1
670	KP780874.2	Avian_orthoavulavirus_1_isolate_ROPI/USA/Allegheny/PA/ND0007199/840/2013	2013	USA	Class II	VI	VI.2.1.1.1
671	KP780875.1	Avian_orthoavulavirus_1_isolate_ND0003553	2013	USA	Class II	VI	VI.2.1.1.1
672	KP780876.1	Avian_orthoavulavirus_1_isolate_ND0003558	2013	USA	Class II	VI	VI.2.1.1.1
673	KP861633.1	Newcastle_disease_virus_isolate_Pigeon/China/SD069/2012	2012	China	Class II	VI	VI.2.1.1.2.2
674	KR014202.1	Avian_orthoavulavirus_1_isolate_APMV-1/Pigeon/Ethiopia/14VIR4296-2/2773/14/2/2014	2014	Ethiopia	Class II	VI	VI.2.1.2
675	KR014203.1	Avian_orthoavulavirus_1_isolate_APMV-1/Pigeon/Ethiopia/14VIR4296-4/2773/14/4/2014	2014	Ethiopia	Class II	VI	VI.2.1.2
676	KR014204.1	Avian_orthoavulavirus_1_isolate_APMV-1/Pigeon/Ethiopia/14VIR4296-6/2773/14/6/2014	2014	Ethiopia	Class II	VI	VI.2.1.2
677	KR014205.1	Avian_orthoavulavirus_1_isolate_APMV-1/Pigeon/Ethiopia/14VIR4296-10/2773/14/10/2014	2014	Ethiopia	Class II	VI	VI.2.1.2
678	KR082484.1	Newcastle_disease_virus_strain_NDV/pigeon/Desouk/Egypt/MS3I/2014	2014	Egypt	Class II	XXI	XXI.1.1
679	KR082486.1	Newcastle_disease_virus_strain_NDV/Dove/Bassioun/Egypt/MS2/2014	2014	Egypt	Class II	VII	VII.1.1
680	KT006286.1	Newcastle_disease_virus_strain_NDV/dove/Desouk/Egypt/MS5/2015	2015	Egypt	Class II	VII	VII.1.1
681	KT163261.1	Avian_orthoavulavirus_1_isolate_Pi/SH/CH/0163/2012	2012	China	Class II	VI	VI.2.1.1.2.2
682	KT163262.1	Avian_orthoavulavirus_1_isolate_Pi/SH/CH/0167/2013	2013	China	Class II	VI	VI.2.1.1.2.2
683	KT163263.1	Avian_orthoavulavirus_1_isolate_Pi/SH/CH/0168/2013	2013	China	Class II	VI	VI.2.1.1.2.2
684	KT163264.1	Avian_orthoavulavirus_1_isolate_Pi/SD/CH/0132/2012	2012	China	Class II	VI	VI.2.1.1.2.1
685	KT223818.1	Avian_orthoavulavirus_1_isolate_NDV/pigeon/China/Qinghai-01/2014	2014	China	Class I	1	1.1.2
686	KT223819.1	Avian_orthoavulavirus_1_isolate_NDV/pigeon/China/Qinghai-02/2014	2014	China	Class I	1	1.1.2
687	KT343662.1	Newcastle_disease_virus_isolate_NDPR2	2012	Iran	Class II	XXI	XXI.1.1
688	KT343663.1	Newcastle_disease_virus_isolate_NDP14	2012	Iran	Class II	XXI	XXI.1.1
689	KT343664.1	Newcastle_disease_virus_isolate_NDP11	2012	Iran	Class II	XXI	XXI.1.1
690	KT343665.1	Newcastle_disease_virus_isolate_NDP17	2012	Iran	Class II	XXI	XXI.1.1
691	KT343666.1	Newcastle_disease_virus_isolate_NDP19	2012	Iran	Class II	VI	VI.2.1.1.2.2
692	KT343667.1	Newcastle_disease_virus_isolate_NDP20	2012	Iran	Class II	I	I.1.1

693	KT343668.1	Newcastle_disease_virus_isolate_NDP21	2012	Iran	Class II	VI	VI.2.2.2
694	KT343669.1	Newcastle_disease_virus_isolate_NDP22	2012	Iran	Class II	VI	VI.2.2.2
695	KT343670.1	Newcastle_disease_virus_isolate_NDP25	2012	Iran	Class II	XXI	XXI.1.1
696	KT343671.1	Newcastle_disease_virus_isolate_NDP27	2012	Iran	Class II	XXI	XXI.1.1
697	KT343672.1	Newcastle_disease_virus_isolate_NDP61	2012	Iran	Class II	XXI	XXI.1.1
698	KT343673.1	Newcastle_disease_virus_isolate_NDP64	2012	Iran	Class II	XXI	XXI.1.1
699	KT343674.1	Newcastle_disease_virus_isolate_NDP79	2012	Iran	Class II	XXI	XXI.1.1
700	KT343675.1	Newcastle_disease_virus_isolate_NDP83	2012	Iran	Class II	XXI	XXI.1.1
701	KT343676.1	Newcastle_disease_virus_isolate_NDP92	2012	Iran	Class II	XXI	XXI.1.1
702	KT343677.1	Newcastle_disease_virus_isolate_NDP93	2012	Iran	Class II	XXI	XXI.1.1
703	KT343678.1	Newcastle_disease_virus_isolate_NDP95	2012	Iran	Class II	XXI	XXI.1.1
704	KT381587.1	Avian_orthoavulavirus_1_isolate_Pigeon/Guangdong/YF1/2014	2014	China	Class I	1	1.1.2
705	KT381592.1	Avian_orthoavulavirus_1_isolate_Pigeon/Guangdong/GZ293/2014	2014	China	Class II	VI	VI.2.1.1.2.2
706	KT381595.1	Avian_orthoavulavirus_1_isolate_Pigeon/Guangdong/GM1/2014	2014	China	Class II	VI	VI.2.1.1.2.1
707	KT381596.1	Avian_orthoavulavirus_1_isolate_Pigeon/Guangdong/GM8/2013	2013	China	Class II	VI	VI.2.2.2
708	KT381601.1	Avian_orthoavulavirus_1_isolate_Pigeon/Guangdong/GZ287/2013	2013	China	Class II	VI	VI.2.1.1.2.2
709	KT381602.1	Avian_orthoavulavirus_1_isolate_Pigeon/Guangdong/GZ288/2013	2013	China	Class II	VI	VI.2.1.1.2.2
710	KT381603.1	Avian_orthoavulavirus_1_isolate_Pigeon/Guangdong/GZ290/2013	2013	China	Class II	VI	VI.2.1.1.2.2
711	KT381604.1	Avian_orthoavulavirus_1_isolate_Pigeon/Guangdong/GZ292/2014	2014	China	Class II	VI	VI.2.1.1.2.2
712	KT381606.1	Avian_orthoavulavirus_1_isolate_Pigeon/Guangdong/GZ289/2014	2014	China	Class II	VI	VI.2.1.1.2.1
713	KT962979.1	Avian_orthoavulavirus_1_isolate_Altai/pigeon/777/2010	2010	Russia	Class II	XXI	XXI.1.1
714	KT965727.1	Avian_orthoavulavirus_1_isolate_columbalivia/KZ/EKO/15/2014	2014	Kazakhstan	Class II	XXI	XXI.1.1
715	KT965728.1	Avian_orthoavulavirus_1_isolate_columbalivia/KZ/Zhambyl/32/2014	2014	Zhambyl	Class II	XXI	XXI.1.1
716	KT965732.1	Avian_orthoavulavirus_1_isolate_columbalivia/KZ/Zhambyl/27/2014	2014	Zhambyl	Class II	XXI	XXI.1.1
717	KU133355.1	Avian_orthoavulavirus_1_isolate_pigeon/Bulgaria/Govedartzi/2002	2002	Bulgaria	Class II	II	II
718	KU133357.1	Avian_orthoavulavirus_1_isolate_Rock_pigeon/Brazil/8705/2009	2009	Brazil	Class II	II	II

719	KU133358.1	Avian_orthoavulavirus_1_isolate_Rock_pigeon/Brazil/UFRGS/2009	2009	Brazil	Class II	II	II
720	KU133359.1	Avian_orthoavulavirus_1_isolate_Rock_pigeon/Brazil/S67A/2009	2009	Brazil	Class II	II	II
721	KU133363.1	Avian_orthoavulavirus_1_isolate_pigeon/Ukraine/Pigeon/Dnipropetrovsk/07/2007	2007	Ukraine	Class II	II	II
722	KU133364.1	Avian_orthoavulavirus_1_isolate_pigeon/Ukraine/Kharkiv/1/2007	2007	Ukraine	Class II	II	II
723	KU133365.1	Avian_orthoavulavirus_1_isolate_pigeon/Ukraine/Kharkiv/2/2007	2007	Ukraine	Class II	II	II
724	KU159667.1	Avian_orthoavulavirus_1_isolate_rock_pigeon/USA/GA/1/2012	2012	USA	Class II	II	II
725	KU159668.1	Avian_orthoavulavirus_1_isolate_rock_pigeon/USA/GA/2/2012	2012	USA	Class II	II	II
726	KU159669.1	Avian_orthoavulavirus_1_isolate_rock_pigeon/USA/GA/3/2012	2012	USA	Class II	II	II
727	KU159670.1	Avian_orthoavulavirus_1_isolate_rock_pigeon/USA/GA/4/2012	2012	USA	Class II	II	II
728	KU159671.1	Avian_orthoavulavirus_1_isolate_rock_pigeon/USA/GA/5/2012	2012	USA	Class II	II	II
729	KU159672.1	Avian_orthoavulavirus_1_isolate_rock_pigeon/USA/GA/6/2012	2012	USA	Class II	II	II
730	KU159673.1	Avian_orthoavulavirus_1_isolate_rock_pigeon/USA/GA/7/2012	2012	USA	Class II	II	II
731	KU159674.1	Avian_orthoavulavirus_1_isolate_rock_pigeon/USA/GA/8/2012	2012	USA	Class II	II	II
732	KU159675.1	Avian_orthoavulavirus_1_isolate_rock_pigeon/USA/GA/9/2012	2012	USA	Class II	II	II
733	KU200240.1	Avian_orthoavulavirus_1_strain_Pigeon/China/Heilongjiang/YA05/2014	2014	China	Class II	II	II
734	KU200241.1	Avian_orthoavulavirus_1_strain_Pigeon/China/Jilin/YJ02/2015	2015	China	Class II	II	II
735	KU200247.1	Avian_orthoavulavirus_1_strain_Pigeon/China/Heilongjiang/TL03/2015	2015	China	Class II	VII	VII.1.1
736	KU200248.1	Avian_orthoavulavirus_1_strain_Pigeon/China/Heilongjiang/LS03/2014	2014	China	Class II	VII	VII.1.1
737	KU200964.1	Pigeon_paramyxovirus_1_isolate_Pigeon/China/GD130/2014	2014	China	Class II	VI	VI.2.1.1.2.2
738	KU200965.1	Pigeon_paramyxovirus_1_isolate_Pigeon/China/GD123/2010	2010	China	Class II	VI	VI.2.1.1.2.2
739	KU200966.1	Pigeon_paramyxovirus_1_isolate_Pigeon/China/GD128/2012	2012	China	Class II	VI	VI.2.1.1.2.2
740	KU377528.1	Pigeon_paramyxovirus_1_isolate_00VIR4400/turtledove/Italy/2000	2000	Italy	Class II	VI	VI.1
741	KU377529.1	Pigeon_paramyxovirus_1_isolate_02VIR1875/turtledove/Italy/2002	2002	Italy	Class II	VI	VI.2.1.1.2.1
742	KU377530.1	Pigeon_paramyxovirus_1_isolate_04VIR434/turtledove/Italy/2004	2004	Italy	Class II	VI	VI.2.1.1.2.1
743	KU377531.1	Pigeon_paramyxovirus_1_isolate_06VIR463-2/pigeon/Italy/2006	2006	Italy	Class II	VI	VI.1
744	KU377532.1	Pigeon_paramyxovirus_1_isolate_08VIR24/turtledove/Italy/2008	2008	Italy	Class II	VI	VI.2.1.1.2.1

745	KU377533.1	Pigeon_paramyxovirus_1_isolate_10VIR7155/turtledove/Italy/2010	2010	Italy	Class II	XXI	XXI.2
746	KU377534.1	Pigeon_paramyxovirus_1_isolate_11VIR5795/pigeon/Italy/2011	2011	Italy	Class II	XXI	XXI.1.2
747	KU377535.1	Pigeon_paramyxovirus_1_isolate_12VIR1876-1/turtledove/Italy/2012	2012	Italy	Class II	XXI	XXI.2
748	KU377536.1	Pigeon_paramyxovirus_1_isolate_12VIR604/turtledove/Italy/2012	2012	Italy	Class II	XXI	XXI.2
749	KU377537.1	Pigeon_paramyxovirus_1_isolate_14VIR8258-2/pigeon/Italy/2014	2014	Italy	Class II	VI	VI.2.1.1.2.1
750	KU522142.1	Pigeon_paramyxovirus_1_isolate_pigeon/Egypt/VRLCU/2014	2014	Egypt	Class II	VI	VI.2.1.1.2.2
751	KU527558.1	Avian_orthoavulavirus_1_strain_Pigeon/China/Jilin/DA08/2015	2015	China	Class II	II	II
752	KU527559.1	Avian_orthoavulavirus_1_strain_Pigeon/China/Jilin/DH09/2015	2015	China	Class II	VI	VI.2.1.1.2.2
753	KU527560.1	Avian_orthoavulavirus_1_strain_Pigeon/China/Jilin/NG05/2015	2015	China	Class II	VI	VI.2.1.1.2.2
754	KU644586.1	Avian_orthoavulavirus_1_isolate_Pi/MZS1-UVAS/2014	2014	Pakistan	Class II	XX	XX
755	KU710277.1	Avian_orthoavulavirus_1_isolate_pigeon/Ukraine/Simferopol/2-26-11/2011	2011	Ukraine	Class II	VII	VII.1.1
756	KU862297.1	Avian_orthoavulavirus_1_isolate_Pigeon/Pak/Lahore/AW-1/2014	2014	Pakistan	Class II	XX	XX
757	KU862298.1	Avian_orthoavulavirus_1_isolate_Pigeon/Pak/Lahore/AW-2/2015	2015	Pakistan	Class II	XXI	XXI.1.2
758	KU862299.1	Avian_orthoavulavirus_1_isolate_Pigeon/Pak/Lahore/AW-3/2015	2015	Pakistan	Class II	XXI	XXI.1.2
759	KU885390.1	Avian_orthoavulavirus_1_isolate_Bhopal_	2015	India	Class II	XXI	XXI.1.2
760	KU885949.1	Avian_orthoavulavirus_1_isolate_Pigeon/MZS-UVAS-Pak/2014	2014	Pakistan	Class II	XXI	XXI.1.2
761	KX065188.1	Avian_orthoavulavirus_1_strain_PPMV-1/QL-01/CH/15	2015	China	Class II	VI	VI.2.1.1.2.1
762	KX097024.1	Pigeon_paramyxovirus_1_strain_N1065/14	2014	Brazil	Class II	VI	VI.2.1.2
763	KX236100.2	Avian_avulavirus_1_isolate_pigeon/Pakistan/Lahore/21A/2015	2015	Pakistan	Class II	XXI	XXI.1.2
764	KX236101.2	Avian_avulavirus_1_isolate_pigeon/Pakistan/Lahore/25A/2015	2015	Pakistan	Class II	XX	XX
765	KX247376.1	Avian_orthoavulavirus_1_strain_PPMV-1/SX-01/Ch/15	2015	China	Class II	VI	VI.2.1.1.2.2
766	KX290116.1	Avian_orthoavulavirus_1_isolate_NDV-8	2016	India	Class II	XVIII	XVIII.1
767	KX290120.1	Avian_orthoavulavirus_1_isolate_NDV-20	2016	India	Class II	XVIII	XVIII.1
768	KX496962.1	Avian_orthoavulavirus_1_isolate_wild_pigeon/Pakistan/Lahore/20A/996/2015	2015	Pakistan	Class II	VII	VII.2
769	KX496964.1	Avian_orthoavulavirus_1_isolate_pigeon/Pakistan/Lahore/23A/997/2015	2015	Pakistan	Class II	VII	VII.2
770	KX496965.1	Avian_orthoavulavirus_1_isolate_pigeon/Pakistan/Lahore/21A/1084/2015	2015	Pakistan	Class II	VII	VII.2

771	KX496967.1	Avian_orthoavulavirus_1_isolate_racing_pigeon/Pakistan/Lahore/24A/1085/2015	2015	Pakistan	Class II	VII	VII.2
772	KX580975.1	Pigeon_paramyxovirus_1_isolate_pigeon/Egypt/Sharkia-1/2014	2014	Egypt	Class II	I	I.1.1
773	KX580976.1	Pigeon_paramyxovirus_1_isolate_pigeon/Egypt/Sharkia-2/2013	2013	Egypt	Class II	XXI	XXI.1.1
774	KX580977.1	Pigeon_paramyxovirus_1_isolate_pigeon/Egypt/Sharkia-4/2014	2014	Egypt	Class II	I	I.1.1
775	KX580978.1	Pigeon_paramyxovirus_1_isolate_pigeon/Egypt/Sharkia-5/2014	2014	Egypt	Class II	I	I.1.1
776	KX580979.1	Pigeon_paramyxovirus_1_isolate_pigeon/Egypt/Sharkia-6/2013	2013	Egypt	Class II	I	I.1.1
777	KX580980.1	Pigeon_paramyxovirus_1_isolate_pigeon/Egypt/Sharkia-10/2014	2014	Egypt	Class II	I	I.1.1
778	KX580981.1	Pigeon_paramyxovirus_1_isolate_pigeon/Egypt/Sharkia-11/2015	2015	Egypt	Class II	I	I.1.1
779	KX580982.1	Pigeon_paramyxovirus_1_isolate_pigeon/Egypt/Sharkia-12/2014	2014	Egypt	Class II	I	I.1.1
780	KX580983.1	Pigeon_paramyxovirus_1_isolate_pigeon/Egypt/Sharkia-13/2014	2014	Egypt	Class II	I	I.1.1
781	KX580984.1	Pigeon_paramyxovirus_1_isolate_pigeon/Egypt/Sharkia-14/2014	2014	Egypt	Class II	I	I.1.1
782	KX580985.1	Pigeon_paramyxovirus_1_isolate_pigeon/Egypt/Sharkia-15/2014	2014	Egypt	Class II	I	I.1.1
783	KX580986.1	Pigeon_paramyxovirus_1_isolate_pigeon/Egypt/Sharkia-17/2014	2014	Egypt	Class II	XXI	XXI.1.1
784	KX580987.1	Pigeon_paramyxovirus_1_isolate_pigeon/Egypt/Sharkia-18/2014	2014	Egypt	Class II	II	II
785	KX580988.1	Pigeon_paramyxovirus_1_isolate_pigeon/Egypt/Sharkia-19/2015	2015	Egypt	Class II	XXI	XXI.1.1
786	KX580989.1	Pigeon_paramyxovirus_1_isolate_pigeon/Egypt/Sharkia-20/2014	2014	Egypt	Class II	I	I.1.1
787	KX580990.1	Pigeon_paramyxovirus_1_isolate_pigeon/Egypt/Sharkia-21/2014	2014	Egypt	Class II	I	I.1.1
788	KX580991.1	Pigeon_paramyxovirus_1_isolate_pigeon/Egypt/Sharkia-22/2014	2014	Egypt	Class II	I	I.1.1
789	KX580992.1	Pigeon_paramyxovirus_1_isolate_pigeon/Egypt/Sharkia-23/2014	2014	Egypt	Class II	I	I.1.1
790	KX580993.1	Pigeon_paramyxovirus_1_isolate_pigeon/Egypt/Sharkia-24/2014	2014	Egypt	Class II	I	I.1.1
791	KX710209.1	Pigeon_paramyxovirus_1_isolate_D167	2015	India	Class II	VI	VI.2.2.2
792	KX710210.1	Pigeon_paramyxovirus_1_isolate_D168	2015	India	Class II	VI	VI.2.1.1.2.2
793	KX761865.1	Avian_orthoavulavirus_1_isolate_HB1103	2011	China	Class II	VI	VI.2.1.1.2.1
794	KX781723.1	Pigeon_paramyxovirus_1_isolate_pigeon/Egypt/Kafrelsheikh-2016	2016	Egypt	Class II	VI	VI.2.2.2
795	KX831612.1	Avian_orthoavulavirus_1_isolate_Pigeon/Portugal/16919/2015	2015	Portugal	Class II	XXI	XXI.2
796	KX831613.1	Avian_orthoavulavirus_1_isolate_Pigeon/Portugal/17991/2015	2015	Portugal	Class II	XXI	XXI.2

797	KX831614.1	Avian_orthoavulavirus_1_isolate_Pigeon/Portugal/19095/2015	2015	Portugal	Class II	XXI	XXI.2
798	KY042127.1	Avian_orthoavulavirus_1_isolate_pigeon/Ukraine/Kharkiv/23-01/967/2013	2013	Ukraine	Class II	XXI	XXI.1.1
799	KY042128.1	Avian_orthoavulavirus_1_isolate_pigeon/Ukraine/Doneck/3/968/2007	2007	Ukraine	Class II	XXI	XXI.1.1
800	KY042129.1	Avian_orthoavulavirus_1_isolate_pigeon/Egypt/Giza/11/1088/2015	2015	Egypt	Class II	XXI	XXI.1.1
801	KY042130.1	Avian_orthoavulavirus_1_isolate_pigeon/Egypt/Helwan/44/1106/2015	2015	Egypt	Class II	XXI	XXI.1.1
802	KY042131.1	Avian_orthoavulavirus_1_isolate_pigeon/Egypt/Qena/56/1107/2015	2015	Egypt	Class II	XXI	XXI.1.1
803	KY042132.1	Avian_orthoavulavirus_1_isolate_pigeon/Egypt/El_Fayom/73/1111/2015	2015	Egypt	Class II	XXI	XXI.1.1
804	KY042133.1	Avian_orthoavulavirus_1_isolate_pigeon/Egypt/El_Fayom/79/1112/2015	2015	Egypt	Class II	XXI	XXI.1.1
805	KY042134.1	Avian_orthoavulavirus_1_isolate_pigeon/Egypt/El_Fayom/84/1113/2015	2015	Egypt	Class II	XXI	XXI.1.1
806	KY042135.1	Avian_orthoavulavirus_1_isolate_pigeon/Pakistan/Lahore/22A/1001/2015	2015	Pakistan	Class II	XX	XX
807	KY042136.1	Avian_orthoavulavirus_1_isolate_pigeon/Pakistan/Lahore/125/2015	2015	Pakistan	Class II	XXI	XXI.1.1
808	KY042137.1	Avian_orthoavulavirus_1_isolate_pigeon/Pakistan/Jhang/115/2015	2015	Pakistan	Class II	XXI	XXI.1.1
809	KY042138.1	Avian_orthoavulavirus_1_isolate_pigeon/Pakistan/Lahore/126/2015	2015	Pakistan	Class II	XXI	XXI.1.1
810	KY042139.1	Avian_orthoavulavirus_1_isolate_pigeon/Pakistan/Lahore/146/2016	2016	Pakistan	Class II	XXI	XXI.1.1
811	KY042140.1	Avian_orthoavulavirus_1_isolate_pigeon/Pakistan/Jallo-Lahore/221A/2016	2016	Pakistan	Class II	XX	XX
812	KY042141.1	Avian_orthoavulavirus_1_isolate_pigeon/Pakistan/Jallo-Lahore/221B/2016	2016	Pakistan	Class II	XX	XX
813	KY174317.1	Pigeon_paramyxovirus_1_isolate_APMV1/Egypt/1/2016	2016	Egypt	Class II	I	I.1.1
814	KY174318.1	Pigeon_paramyxovirus_1_isolate_APMV1/Egypt/2/2016	2016	Egypt	Class II	XXI	XXI.1.2
815	KY174319.1	Pigeon_paramyxovirus_1_isolate_APMV1/Egypt/3/2016	2016	Egypt	Class II	I	I.1.1
816	KY174320.1	Pigeon_paramyxovirus_1_isolate_APMV1/Egypt/4/2016	2016	Egypt	Class II	I	I.1.1
817	KY569334.1	Avian_orthoavulavirus_1_isolate_pigeon/Malaysia/Selangor/1676/2006	2006	Malaysia	Class II	VI	VI.2.1.1.2.1
818	KY569335.1	Avian_orthoavulavirus_1_isolate_pigeon/Malaysia/Penang/14879/2011	2011	Malaysia	Class II	VI	VI.2.1.1.2.2
819	KY788667.1	Avian_orthoavulavirus_1_isolate_European_turtle_dove/Ch/GD/GZ23/2015	2015	China	Class II	VI	VI.2.1.1.2.2
820	KY788669.1	Avian_orthoavulavirus_1_isolate_Spotted_dove/Ch/GD/GZ232/2014	2014	China	Class II	IX	IX
821	LC055504.1	Pigeon_paramyxovirus_1strain:_PPMV1-2CN	2014	Iran	Class II	XXI	XXI.1.1
822	LC055505.1	Pigeon_paramyxovirus_1strain:_PPMV1-7TS	2014	Iran	Class II	XXI	XXI.1.1

823	LC055506.1	Pigeon_paramyxovirus_1strain:_PPMV1-8TS	2014	Iran	Class II	XXI	XXI.1.1
824	LC055507.1	Pigeon_paramyxovirus_1strain:_PPMV1-14B	2013	Iran	Class II	XXI	XXI.1.1
825	LC055508.1	Pigeon_paramyxovirus_1strain:_PPMV1-16T	2014	Iran	Class II	XXI	XXI.1.1
826	LC055509.1	Pigeon_paramyxovirus_1strain:_PPMV1-16CT	2014	Iran	Class II	XXI	XXI.1.1
827	LC055510.1	Pigeon_paramyxovirus_1strain:_PPMV1-18TS	2014	Iran	Class II	XXI	XXI.1.1
828	LC055511.1	Pigeon_paramyxovirus_1strain:_PPMV1-18CS	2014	Iran	Class II	XXI	XXI.1.1
829	LC055512.1	Pigeon_paramyxovirus_1strain:_PPMV1-21TS	2014	Iran	Class II	XXI	XXI.1.1
830	LC055513.1	Pigeon_paramyxovirus_1strain:_PPMV1-21CS	2014	Iran	Class II	XXI	XXI.1.1
831	LC055514.1	Pigeon_paramyxovirus_1strain:_PPMV1-51B	2014	Iran	Class II	XXI	XXI.1.1
832	LC055515.1	Pigeon_paramyxovirus_1strain:_PPMV1-52CT	2014	Iran	Class II	XXI	XXI.1.1
833	LC500946.1	Avian_orthoavulavirus_1_APMV1/pigeon/Japan/Kumamoto/2001	2001	Japan	Class II	VI	VI.2.1.1.2.1
834	LC500947.1	Avian_orthoavulavirus_1_APMV1/pigeon/Japan/Hiroshima/2002	2002	Japan	Class II	VI	VI.2.1.1.2.1
835	LC500948.1	Avian_orthoavulavirus_1_APMV1/pigeon/Japan/Shizuoka/2002	2002	Japan	Class II	VI	VI.2.1.1.2.1
836	LC500951.1	Avian_orthoavulavirus_1_APMV1/pigeon/Japan/Mie/2002	2002	Japan	Class II	VI	VI.2.1.1.2.1
837	LC500953.1	Avian_orthoavulavirus_1_APMV1/pigeon/Japan/Saitama-1/2003	2003	Japan	Class II	VI	VI.2.1.1.2.1
838	LC500954.1	Avian_orthoavulavirus_1_APMV1/pigeon/Japan/Saitama-2/2003	2003	Japan	Class II	VI	VI.2.1.1.2.1
839	LC500959.1	Avian_orthoavulavirus_1_APMV1/pigeon/Japan/Tokyo/2006	2006	Japan	Class II	VI	VI.2.1.1.2.1
840	LC500960.1	Avian_orthoavulavirus_1_APMV1/pigeon/Japan/Niigata/2007	2007	Japan	Class II	VI	VI.2.1.1.2.1
841	LC552053.1	Pigeon_paramyxovirus_1_PPMV1/BR/CCZ/2019	2019	Brazil	Class II	VI	VI.2.1.2
842	MF100759.1	Avian_orthoavulavirus_1_isolate_P/GD/GZ/178/2016	2016	China	Class I	1	1.1.2
843	MF100760.1	Avian_orthoavulavirus_1_isolate_P/GD/DG/466/2016	2016	China	Class I	1	1.1.2
844	MF100761.1	Avian_orthoavulavirus_1_isolate_P/GD/HZ/599/2016	2016	China	Class I	1	1.1.2
845	MF179097.1	Pigeon_paramyxovirus_1_isolate_Pigeon/Tehran/GH01/2014	2014	Tehran	Class II	XXI	XXI.1.1
846	MF179098.1	Pigeon_paramyxovirus_1_isolate_Pigeon/Tehran/GH02/2014	2014	Tehran	Class II	XXI	XXI.1.1
847	MF179099.1	Pigeon_paramyxovirus_1_isolate_Pigeon/Tehran/GH03/2014	2014	Tehran	Class II	XXI	XXI.1.1
848	MF179100.1	Pigeon_paramyxovirus_1_isolate_Pigeon/Tehran/GH04/2014	2014	Tehran	Class II	XXI	XXI.1.1

849	MF179101.1	Pigeon_paramyxovirus_1_isolate_Pigeon/Tehran/GH05/2014	2014	Tehran	Class II	XXI	XXI.1.1
850	MF278934.1	Avian_orthoavulavirus_1_strain_PG/GZ/HD/PN/2011	2011	China	Class II	XX	XX
851	MF375924.1	Pigeon_paramyxovirus_1_isolate_pigeon/Egypt/KFS/2017/2	2017	Egypt	Class II	XXI	XXI.1.1
852	MF580815.1	Pigeon_paramyxovirus_1_isolate_pi/CH/GX0094/2011	2011	China	Class II	VI	VI.2.1.1.2.2
853	MF580816.1	Pigeon_paramyxovirus_1_isolate_pi/CH/GX0001/2012	2012	China	Class II	VI	VI.2.1.1.2.2
854	MF580817.1	Pigeon_paramyxovirus_1_isolate_pi/CH/GX0012/2012	2012	China	Class II	VI	VI.2.1.1.2.2
855	MF580818.1	Pigeon_paramyxovirus_1_isolate_pi/CH/GX0019/2012	2012	China	Class II	VI	VI.2.1.1.2.2
856	MF580819.1	Pigeon_paramyxovirus_1_isolate_pi/CH/GX0022/2012	2012	China	Class II	VI	VI.2.1.1.2.2
857	MF580820.1	Pigeon_paramyxovirus_1_isolate_pi/CH/GX0029/2012	2012	China	Class II	VI	VI.2.1.1.2.2
858	MF580821.1	Pigeon_paramyxovirus_1_isolate_pi/CH/GX0031/2012	2012	China	Class II	VI	VI.2.1.1.2.2
859	MF580822.1	Pigeon_paramyxovirus_1_isolate_pi/CH/GX0034/2012	2012	China	Class II	VI	VI.2.1.1.2.2
860	MF580823.1	Pigeon_paramyxovirus_1_isolate_pi/CH/GX0505/2012	2012	China	Class II	VI	VI.2.1.1.2.2
861	MF580824.1	Pigeon_paramyxovirus_1_isolate_pi/CH/GX0004/2013	2013	China	Class II	VI	VI.2.1.1.2.2
862	MF580825.1	Pigeon_paramyxovirus_1_isolate_pi/CH/GX0119/2015	2015	China	Class II	VI	VI.2.1.1.2.2
863	MF580826.1	Pigeon_paramyxovirus_1_isolate_pi/CH/GX0126/2015	2015	China	Class II	VI	VI.2.1.1.2.2
864	MF580827.1	Pigeon_paramyxovirus_1_isolate_pi/CH/GX1103/2015	2015	China	Class II	VI	VI.2.1.1.2.2
865	MF614961.1	Pigeon_paramyxovirus_1_isolate_pigeon/Egypt/KFS/2017/5	2017	Egypt	Class II	II	II
866	MF681711.1	Avian_orthoavulavirus_1_isolate_4533	2017	Namibia	Class II	VI	VI.2.1.1.2.1
867	MF681712.1	Avian_orthoavulavirus_1_isolate_5072	2016	Namibia	Class II	VI	VI.2.1.1.2.1
868	MF681713.1	Avian_orthoavulavirus_1_isolate_7808	2016	Namibia	Class II	I	I.1.1
869	MF681714.1	Avian_orthoavulavirus_1_isolate_7855	2016	Namibia	Class II	I	I.1.1
870	MF681715.1	Avian_orthoavulavirus_1_isolate_8199	2016	Namibia	Class II	VI	VI.2.1.1.2.1
871	MF681716.1	Avian_orthoavulavirus_1_isolate_8636	2016	Namibia	Class II	I	I.1.1
872	MG018201.1	Avian_orthoavulavirus_1_isolate_Rock_Pigeon/USA/MA/1188-kidney/24998-1G/2014	2014	USA	Class II	VI	VI.2.1.1.1
873	MG018202.1	Avian_orthoavulavirus_1_isolate_Eurasian_Collared-Dove/USA/MT/1177-kidney/22919-1-B/2010	2010	USA	Class II	VI	VI.2.1.1.1
874	MG018203.1	Avian_orthoavulavirus_1_isolate_Eurasian_Collared-Dove/USA/MT/1177-liver/22919-1-B/2010	2010	USA	Class II	VI	VI.2.1.1.1

875	MG018204.1	Avian_orthoavulavirus_1_isolate_Eurasian_Collared-Dove/USA/MT/1177-spleen/22919-1-B/2010	2010	USA	Class II	VI	VI.2.1.1.1
876	MG018205.1	Avian_orthoavulavirus_1_isolate_Eurasian_Collared-Dove/USA/TX/1179-kidney/26041-3-C/2014	2014	USA	Class II	VI	VI.2.1.1.1
877	MG018206.1	Avian_orthoavulavirus_1_isolate_Eurasian_Collared-Dove/USA/TX/1180-kidney/26041-4-C/2014	2014	USA	Class II	VI	VI.2.1.1.1
878	MG018207.1	Avian_orthoavulavirus_1_isolate_Eurasian_Collared-Dove/USA/TX/1180-liver/26041-4-C/2014	2014	USA	Class II	VI	VI.2.1.1.1
879	MG018208.1	Avian_orthoavulavirus_1_isolate_Eurasian_Collared-Dove/USA/UT/1181-spleen/26594-1-A/2015	2015	USA	Class II	VI	VI.2.1.1.1
880	MG018209.1	Avian_orthoavulavirus_1_isolate_Eurasian_Collared-Dove/USA/TX/1182-spleen/26877-2-C/2016	2016	USA	Class II	VI	VI.2.1.1.1
881	MG018210.1	Avian_orthoavulavirus_1_isolate_Eurasian_Collared-Dove/USA/TX/1184-kidney/26981-2-C/2015	2015	USA	Class II	VI	VI.2.1.1.1
882	MG018211.1	Avian_orthoavulavirus_1_isolate_Eurasian_Collared-Dove/USA/TX/1185-kidney/26981-3-A/2015	2015	USA	Class II	VI	VI.2.1.1.1
883	MG018212.1	Avian_orthoavulavirus_1_isolate_Rock_Pigeon/USA/MA/1188-spleen/24998-1-B/2014	2014	USA	Class II	VI	VI.2.1.1.1
884	MG018213.1	Avian_orthoavulavirus_1_isolate_Eurasian_Collared-Dove/USA/KS/1191-spleen/W16-453-3/2016	2016	USA	Class II	VI	VI.2.1.1.1
885	MG018214.1	Avian_orthoavulavirus_1_isolate_Eurasian_Collared-Dove/USA/KS/1192-kidney/W16-454-2/2016	2016	USA	Class II	VI	VI.2.1.1.1
886	MG018215.1	Avian_orthoavulavirus_1_isolate_Eurasian_Collared-Dove/USA/KS/1194-kidney/W16-456-2/2016	2016	USA	Class II	VI	VI.2.1.1.1
887	MG018216.1	Avian_orthoavulavirus_1_isolate_Eurasian_Collared-Dove/USA/KS/1195-kidney/W16-457-2/2016	2016	USA	Class II	VI	VI.2.1.1.1
888	MG018217.1	Avian_orthoavulavirus_1_isolate_Eurasian_Collared-Dove/USA/TX/1180-spleen/26041-4-C/2014	2014	USA	Class II	VI	VI.2.1.1.1
889	MG018218.1	Avian_orthoavulavirus_1_isolate_Rock_Pigeon/USA/PA/1189-kidney/23844-3-C/2012	2012	USA	Class II	VI	VI.2.1.1.1
890	MG018219.1	Avian_orthoavulavirus_1_isolate_Eurasian_Collared-Dove/USA/KS/1193-kidney/W16-455-2/2016	2016	USA	Class II	VI	VI.2.1.1.1
891	MG018220.1	Avian_orthoavulavirus_1_isolate_Eurasian_Collared-Dove/USA/KS/1191-kidney/W16-453-3/2016	2016	USA	Class II	VI	VI.2.1.1.1
892	MG018221.1	Avian_orthoavulavirus_1_isolate_Eurasian_Collared-Dove/USA/TX/1182-kidney/26877-2-C/2016	2016	USA	Class II	VI	VI.2.1.1.1
893	MG018222.1	Avian_orthoavulavirus_1_isolate_Eurasian_Collared-Dove/USA/UT/1181-liver/26594-1-D/2015	2015	USA	Class II	VI	VI.2.1.1.1
894	MG018223.1	Avian_orthoavulavirus_1_isolate_Eurasian_Collared-Dove/USA/UT/1181-kidney/26594-1-D/2015	2015	USA	Class II	VI	VI.2.1.1.1
895	MG456676.1	Avian_orthoavulavirus_1_strain_APMV-1/collared_dove/Iran/2014	2014	Iran	Class II	XXI	XXI.2
896	MG840652.1	Avian_orthoavulavirus_1_strain_pigeon/Fujian/1233/2014	2014	China	Class II	VI	VI.2.1.1.2.2
897	MG840653.1	Avian_orthoavulavirus_1_strain_pigeon/Guizhou/1050/2017	2017	China	Class II	VI	VI.2.1.1.2.2
898	MG840654.1	Avian_orthoavulavirus_1_strain_pigeon/Ningxia/2068/2016	2016	China	Class II	VI	VI.2.1.1.2.2
899	MG840655.1	Avian_orthoavulavirus_1_strain_pigeon/Qinghai/1325/2017	2017	China	Class II	VI	VI.2.1.1.2.2
900	MG840656.1	Avian_orthoavulavirus_1_strain_pigeon/Qinghai/1344/2017	2017	China	Class II	VI	VI.2.1.1.2.2

901	MG840657.1	Avian_orthoavulavirus_1_strain_pigeon/Sichuan/2045/2014	2014	China	Class II	VI	VI.2.1.1.2.2
902	MG840658.1	Avian_orthoavulavirus_1_strain_pigeon/Yunnan/1205/2015	2015	China	Class II	VI	VI.2.1.1.2.2
903	MG840659.1	Avian_orthoavulavirus_1_strain_pigeon/Yunnan/1336/2015	2015	China	Class II	VI	VI.2.1.1.2.2
904	MG840660.1	Avian_orthoavulavirus_1_strain_pigeon/Yunnan/1453/2017	2017	China	Class II	VI	VI.2.1.1.2.2
905	MG878084.1	Pigeon_paramyxovirus_1_strain_Pi/CH/LN/2017	2017	China	Class II	VI	VI.2.1.1.2.2
906	MH044693.1	Avian_orthoavulavirus_1_isolate_Eurasian_collared_dove/Iran/Konarak/Barin/2017	2017	Iran	Class II	XXI	XXI.2
907	MH105248.1	Avian_orthoavulavirus_1_strain_pigeon/Yunnan/1135/2017	2017	China	Class II	VII	VII.2
908	MH247188.1	Avian_orthoavulavirus_1_strain_Pg/IR/MAM39/2017	2017	Iran	Class II	XXI	XXI.1.1
909	MH289936.1	Avian_orthoavulavirus_1_strain_NDV/Pigeon/CN/JX/163X/2016	2016	China	Class I	1	1.1.2
910	MH371057.1	Avian_orthoavulavirus_1_isolate_PHL137866	2011	Israel	Class II	VI	VI.2.1.2
911	MH371091.1	Avian_orthoavulavirus_1_isolate_PHL133813	2011	Israel	Class II	VI	VI.2.1.2
912	MH377247.1	Avian_orthoavulavirus_1_isolate_PHL37114.2	2008	Israel	Class II	VI	VI.2.1.2
913	MH377248.1	Avian_orthoavulavirus_1_isolate_PHL37114	2008	Israel	Class II	VII	VII.1.1
914	MH377250.1	Avian_orthoavulavirus_1_isolate_PHL76558	2009	Israel	Class II	VII	VII.1.1
915	MH377298.1	Avian_orthoavulavirus_1_isolate_PHL264746	2010	Israel	Class II	VI	VI.2.1.1.2.2
916	MH377302.1	Avian_orthoavulavirus_1_isolate_PHL264752	2012	Israel	Class II	VI	VI.2.1.2
917	MH577764.1	Avian_orthoavulavirus_1_strain_NDV/P/17/RSP-1_	2017	India	Class II	II	II
918	MH717070.1	Avian_orthoavulavirus_1_isolate_Pigeon/AJK/AW-p54/2018	2018	Pakistan	Class II	XXI	XXI.1.1
919	MH717071.1	Avian_orthoavulavirus_1_isolate_Pigeon/AJK/AW-p51/2018	2018	Pakistan	Class II	XX	XX
920	MH717072.1	Avian_orthoavulavirus_1_isolate_Pigeon/AJK/AW-p52/2018	2018	Pakistan	Class II	XX	XX
921	MH717073.1	Avian_orthoavulavirus_1_isolate_Pigeon/AJK/AW-p53/2018	2018	Pakistan	Class II	XX	XX
922	MH723605.1	Avian_orthoavulavirus_1_isolate_3pi/Egypt/ElSharkia/2016	2016	Egypt	Class II	XXI	XXI.1.1
923	MH723606.1	Avian_orthoavulavirus_1_isolate_1pi/Egypt/ElSharkia/2016	2016	Egypt	Class II	XXI	XXI.1.1
924	MH723607.1	Avian_orthoavulavirus_1_isolate_2pi/Egypt/ElSharkia/2016	2016	Egypt	Class II	XXI	XXI.1.1
925	MH723608.1	Avian_orthoavulavirus_1_isolate_5pi/Egypt/ElSharkia/2016	2016	Egypt	Class II	XXI	XXI.1.1
926	MH723609.1	Avian_orthoavulavirus_1_isolate_10pi/Egypt/ElSharkia/2016	2016	Egypt	Class II	XXI	XXI.1.1

927	MH723610.1	Avian_orthoavulavirus_1_isolate_8pi/Egypt/ElSharkia/2016	2016	Egypt	Class II	XXI	XXI.1.1
928	MH723611.1	Avian_orthoavulavirus_1_isolate_4pi/Egypt/ElSharkia/2016	2016	Egypt	Class II	XXI	XXI.1.1
929	MH807446.1	Pigeon_paramyxovirus_1_isolate_PPMV-1/BJ-01/CH	2016	China	Class II	VI	VI.2.1.1.2.2
930	MH996902.1	Avian_orthoavulavirus_1_isolate_pigeon/Bulgaria/Septemvriitsi/1157/1982	1982	Bulgaria	Class II	V	V.3
931	MH996904.1	Avian_orthoavulavirus_1_isolate_pigeon/Bulgaria/NovoSelo/1161/1995	1955	Bulgaria	Class II	III	III
932	MH996922.1	Avian_orthoavulavirus_1_isolate_pigeon/Nigeria/KT/MSH/15C/N2/689/2009	2009	Nigeria	Class II	XIV	XIV.2
933	MH996943.1	Avian_orthoavulavirus_1_isolate_pigeon/Nigeria/YB/GSH1/4-6T-XIV/N3/713/2009	2009	Nigeria	Class II	XIV	XIV.2
934	MH996944.1	Avian_orthoavulavirus_1_isolate_pigeon/Nigeria/YB/GSH1/4-6T-XVII/N3/713/2009	2009	Nigeria	Class II	XVII	XVII
935	MH996953.1	Avian_orthoavulavirus_1_isolate_pigeon/Nigeria/Kazaure/VRD231/42/2007	2007	Nigeria	Class II	XXI	XXI.1.1
936	MH996979.1	Avian_orthoavulavirus_1_isolate_pigeon/Nigeria/Gumel/VRD37A/51/2008	2008	Nigeria	Class II	VI	VI.2.1.2
937	MH996992.1	Avian_orthoavulavirus_1_isolate_pigeon/Nigeria/Alaba_rago/VRD32/15/32/2015	2015	Nigeria	Class II	VI	VI.2.1.2
938	MK005973.1	Avian_orthoavulavirus_1_isolate_pigeon/Egypt/Souqal-GomaaMarket/Cairo/39/CL-G23/1105/2015	2015	Egypt	Class II	XXI	XXI.1.1
939	MK005974.1	Avian_orthoavulavirus_1_isolate_pigeon/Egypt/Cairo/17/CL/PG-G2/2015	2015	Egypt	Class II	VII	VII.2
940	MK005995.1	Avian_orthoavulavirus_1_isolate_pigeon/Egypt/Souqal-GomaaMarket/Cairo/19/CL-G3/1090/2015	2015	Egypt	Class II	VII	VII.1.1
941	MK005996.1	Avian_orthoavulavirus_1_isolate_pigeon/Egypt/Souqal-GomaaMarket/Cairo/27/OP-G9/1091/2015	2015	Egypt	Class II	VII	VII.1.1
942	MK005997.1	Avian_orthoavulavirus_1_isolate_pigeon/Egypt/Souqal-GomaaMarket/Cairo/28/OP-G27/1109/2015	2015	Egypt	Class II	VII	VII.1.1
943	MK005998.1	Avian_orthoavulavirus_1_isolate_pigeon/Egypt/1095/Giza/15/CL/PG/1095/2015	2015	Egypt	Class II	VII	VII.1.1
944	MK005999.1	Avian_orthoavulavirus_1_isolate_pigeon/Egypt/1108/Cairo/24/OP/PG/1108/2015	2015	Egypt	Class II	VII	VII.1.1
945	MK006024.1	Avian_orthoavulavirus_1_isolate_pigeon/Vietnam/484/2002	2002	Vietnam	Class II	I	I.1.1
946	MK040379.1	Avian_orthoavulavirus_1_strain_pigeon/California/B1800323-2.1/2018	2018	USA	Class II	V	V.1
947	MK046916.1	Avian_orthoavulavirus_1_isolate_dove/Mexico/Jalisco/Ave2-01/2017	2017	Mexico	Class II	V	V.2
948	MK046917.1	Avian_orthoavulavirus_1_isolate_pigeon/Mexico/Jalisco/Ave3-01/2017	2017	Mexico	Class II	V	V.2
949	MK046918.1	Avian_orthoavulavirus_1_isolate_pigeon/Mexico/Jalisco/Ave4-01/2017	2017	Mexico	Class II	V	V.2
950	MK046919.1	Avian_orthoavulavirus_1_isolate_pigeon/Mexico/Jalisco/Ave5-01/2017	2017	Mexico	Class II	V	V.2
951	MK046921.1	Avian_orthoavulavirus_1_isolate_pigeon/Mexico/Jalisco/LV-7213/2017	2017	Mexico	Class II	VI	VI.2.1.1.1
952	MK335466.1	Avian_orthoavulavirus_1_strain_pi/CH/LNPPMV17_	2017	China	Class II	VI	VI.2.1.1.2.2

953	MK469964.1	Pigeon_paramyxovirus_1_strain_Pi/CH/GXG20/2015	2015	China	Class II	VI	VI.2.1.1.2.2
954	MK469965.1	Pigeon_paramyxovirus_1_strain_Pi/CH/GXG29/2016	2016	China	Class II	VI	VI.2.1.1.2.2
955	MK469966.1	Pigeon_paramyxovirus_1_strain_Pi/CH/GXG6/2015	2015	China	Class II	VI	VI.2.1.1.2.1
956	MK469967.1	Pigeon_paramyxovirus_1_strain_Pi/CH/GXG33/2016	2016	China	Class II	VI	VI.2.1.1.2.2
957	MK469968.1	Pigeon_paramyxovirus_1_strain_Pi/CH/GXG35/2017	2017	China	Class II	VI	VI.2.1.1.2.2
958	MK469969.1	Pigeon_paramyxovirus_1_strain_Pi/CH/GXG2/2012	2012	China	Class II	VI	VI.2.1.1.2.1
959	MK469970.1	Pigeon_paramyxovirus_1_strain_Pi/CH/GXG3/2012	2012	China	Class II	VI	VI.2.1.1.2.2
960	MK469971.1	Pigeon_paramyxovirus_1_strain_Pi/CH/GXG6/2013	2013	China	Class II	VI	VI.2.1.1.2.2
961	MK469972.1	Pigeon_paramyxovirus_1_strain_Pi/CH/GXG7/2014	2014	China	Class II	VI	VI.2.1.1.2.1
962	MK469973.1	Pigeon_paramyxovirus_1_strain_Pi/CH/GXG13/2014	2014	China	Class II	VI	VI.2.1.1.2.2
963	MK469974.1	Pigeon_paramyxovirus_1_strain_Pi/CH/GXG16/2013	2013	China	Class II	VI	VI.2.1.1.2.1
964	MK516200.1	Avian_orthoavulavirus_1_strain_Pigeon/China/SD01/2013	2013	China	Class II	VI	VI.2.1.1.2.2
965	MK516201.1	Avian_orthoavulavirus_1_strain_Pigeon/China/SD03/2013	2013	China	Class II	VI	VI.2.1.1.2.2
966	MK544894.1	Avian_orthoavulavirus_1_isolate_pigeon/NDV/India/IVRI/Bareilly/06/2017	2017	India	Class II	XXI	XXI.1.2
967	MK561384.1	Avian_orthoavulavirus_1_strain_AAvV-1/Pigeon/UAE/P783/2017	2017	United Arab Emirates	Class II	XXI	XXI.1.2
968	MK561387.1	Avian_orthoavulavirus_1_strain_AAvV-1/Pigeon/UAE/P1071/2016	2016	United Arab Emirates	Class II	XX	XX
969	MK561389.1	Avian_orthoavulavirus_1_strain_AAvV-1/Pigeon/UAE/P1127/2017	2017	United Arab Emirates	Class II	XX	XX
970	MK561401.1	Avian_orthoavulavirus_1_strain_AAvV-1/Pigeon/UAE/E5633/2016	2016	United Arab Emirates	Class II	XXI	XXI.1.2
971	MK561404.1	Avian_orthoavulavirus_1_strain_AAvV-1/Pigeon/UAE/E5638/2016	2016	United Arab Emirates	Class II	XVII	XVII
972	MK592884.1	Pigeon_paramyxovirus_1_isolate_pigeon/Iran/UT-EGV/2018	2018	Iran	Class II	XXI	XXI.1.1
973	MK693035.1	Avian_orthoavulavirus_1_isolate_AAvV-1/rock_dove/Southern_Kazakhstan/6444/2014	2014	Kazakhstan	Class II	XXI	XXI.1.1

974	MK749297.1	Pigeon_paramyxovirus_1_strain_Pi/CH/GXG1/2012	2012	China	Class II	VI	VI.2.1.1.2.2
975	MK749298.1	Pigeon_paramyxovirus_1_strain_Pi/CH/GXG22/2015	2015	China	Class II	VI	VI.2.1.1.2.2
976	MK749299.1	Pigeon_paramyxovirus_1_strain_Pi/CH/GXG24/2015	2015	China	Class II	VI	VI.2.1.1.2.2
977	MK749300.1	Pigeon_paramyxovirus_1_strain_Pi/CH/GXG25/2015	2015	China	Class II	VI	VI.2.1.1.2.2
978	MK749301.1	Pigeon_paramyxovirus_1_strain_Pi/CH/GXG28/2016	2016	China	Class II	VI	VI.2.1.1.2.2
979	MK749302.1	Pigeon_paramyxovirus_1_strain_Pi/CH/GXG31/2016	2016	China	Class II	VI	VI.2.1.1.2.2
980	MK764392.1	Avian_orthoavulavirus_1_isolate_TJ/1/15/Pi	2015	China	Class II	VI	VI.2.1.1.2.2
981	MK896851.1	Pigeon_paramyxovirus_1_isolate_pi/CH/SD0315/2018	2018	China	Class II	VI	VI.2.1.1.2.2
982	MK937786.1	Avian_orthoavulavirus_1_strain_ChinaSHJD132017	2017	China	Class II	VII	VII.1.1
983	MK973060.1	Avian_orthoavulavirus_1_strain_pigeon/hebei/08/2017	2017	China	Class II	VI	VI.2.1.1.2.2
984	MN087446.1	UNVERIFIED:_Avian_orthoavulavirus_1_strain_AAV1/pigeon/Iraq	?	Iraq	Class II	II	II
985	MN164450.1	Pigeon_paramyxovirus_1_isolate_PPMV-1/INDIA/1/2019	2019	India	Class II	XXI	XXI.1.2
986	MN164451.1	Pigeon_paramyxovirus_1_isolate_PPMV-1/INDIA/2/2019	2019	India	Class II	XXI	XXI.1.2
987	MN164452.1	Pigeon_paramyxovirus_1_isolate_PPMV-1/INDIA/3/2019	2019	India	Class II	XXI	XXI.1.2
988	MN164453.1	Pigeon_paramyxovirus_1_isolate_PPMV-1/INDIA/4/2019	2019	India	Class II	XXI	XXI.1.2
989	MN413534.1	Avian_orthoavulavirus_1_isolate_2011-3311-0004	2011	Australia	Class II	VI	VI.2.1.1.2.2
990	MN413535.1	Avian_orthoavulavirus_1_isolate_2011-3314	2011	Australia	Class II	VI	VI.2.1.1.2.2
991	MN413536.1	Avian_orthoavulavirus_1_isolate_2011-3313	2011	Australia	Class II	VI	VI.2.1.1.2.2
992	MN413537.1	Avian_orthoavulavirus_1_isolate_2011-3312	2011	Australia	Class II	VI	VI.2.1.1.2.2
993	MN413538.1	Avian_orthoavulavirus_1_isolate_2011-3315	2011	Australia	Class II	VI	VI.2.1.1.2.2
994	MN416768.1	Avian_orthoavulavirus_1_strain_SpottedDove/China/2008	2008	China	Class II	IX	IX
995	MN422262.1	Avian_orthoavulavirus_1_isolate_Pg/IR/AMMM160/2019	2019	Iran	Class II	VI	VI.2.1.2
996	MN462666.1	Avian_orthoavulavirus_1_isolate_2011-3132-3	2011	Australia	Class II	VI	VI.2.1.1.2.2
997	MN477454.1	Pigeon_paramyxovirus_1_strain_Pi/CH/GXG44/2018	2018	China	Class II	VI	VI.2.1.1.2.1
998	MN519685.1	Avian_orthoavulavirus_1_strain_NDV/Egypt/P/MR82/2018	2018	Egypt	Class II	XVIII	XVIII.2
999	MN519686.1	Avian_orthoavulavirus_1_strain_NDV/Egypt/P/MR84/2018	2018	Egypt	Class II	XVIII	XVIII.2

1000	MN610433.1	Pigeon_paramyxovirus_1_isolate_R1/Dakahlia/2015	2015	Egypt	Class II	XXI	XXI.1.1
1001	MN610434.1	Pigeon_paramyxovirus_1_isolate_R6/Dakahlia/2014	2014	Egypt	Class II	XXI	XXI.1.1
1002	MN610435.1	Pigeon_paramyxovirus_1_isolate_R7/Dakahlia/2015	2015	Egypt	Class II	XXI	XXI.1.1
1003	MN610436.1	Pigeon_paramyxovirus_1_isolate_R9/Dakahlia/2018	2018	Egypt	Class II	XXI	XXI.1.1
1004	MN615883.1	Avian_orthoavulavirus_1_isolate_Pg/IR/AMMM117/2018	2018	Iran	Class II	I	I.1.1
1005	MN632518.1	Avian_orthoavulavirus_1_isolate_pigeon/Taiwan/AHRI43/2010	2010	China	Class II	VI	VI.2.1.1.2.1
1006	MN632519.1	Avian_orthoavulavirus_1_isolate_pigeon/Taiwan/AHRI68/2012	2012	China	Class II	VI	VI.2.1.1.2.1
1007	MN632520.1	Avian_orthoavulavirus_1_isolate_pigeon/Taiwan/AHRI107/2016	2016	China	Class II	VI	VI.2.1.1.2.2
1008	MN632521.1	Avian_orthoavulavirus_1_isolate_pigeon/Taiwan/AHRI111/2017	2017	China	Class II	VI	VI.2.1.1.2.2
1009	MN632537.1	Avian_orthoavulavirus_1_isolate_dove/Taiwan/AHRI113/2017	2017	China	Class II	VI	VI.2.1.1.2.2
1010	MN632538.1	Avian_orthoavulavirus_1_isolate_pigeon/Taiwan/AHRI114/2017	2017	China	Class II	VI	VI.2.1.1.2.2
1011	MN632539.1	Avian_orthoavulavirus_1_isolate_dove/Taiwan/AHRI115/2017	2017	China	Class II	VI	VI.2.1.1.2.2
1012	MN632540.1	Avian_orthoavulavirus_1_isolate_dove/Taiwan/AHRI116/2017	2017	China	Class II	VI	VI.2.1.1.2.2
1013	MN632541.1	Avian_orthoavulavirus_1_isolate_dove/Taiwan/AHRI117/2017	2017	China	Class II	VI	VI.2.1.1.2.2
1014	MN632542.1	Avian_orthoavulavirus_1_isolate_dove/Taiwan/AHRI118/2017	2017	China	Class II	VI	VI.2.1.1.2.2
1015	MN632543.1	Avian_orthoavulavirus_1_isolate_dove/Taiwan/AHRI120/2017	2017	China	Class II	VI	VI.2.1.1.2.2
1016	MN632544.1	Avian_orthoavulavirus_1_isolate_pigeon/Taiwan/AHRI121/2017	2017	China	Class II	VI	VI.2.1.1.2.2
1017	MN632545.1	Avian_orthoavulavirus_1_isolate_dove/Taiwan/AHRI123/2017	2017	China	Class II	VI	VI.2.1.1.2.2
1018	MN632547.1	Avian_orthoavulavirus_1_isolate_dove/Taiwan/AHRI126/2017	2017	China	Class II	VI	VI.2.1.1.2.2
1019	MN632548.1	Avian_orthoavulavirus_1_isolate_pigeon/Taiwan/AHRI133/2018	2018	China	Class II	VI	VI.2.1.1.2.2
1020	MN636822.1	Avian_orthoavulavirus_1_isolate_Pg/IR/AMMM168/2019	2019	Iran	Class II	XXI	XXI.1.1
1021	MN700940.1	Pigeon_paramyxovirus_1_isolate_Pi/CH/YQ/2012	2012	China	Class II	VI	VI.2.1.1.2.2
1022	MN830309.1	UNVERIFIED:_Pigeon_paramyxovirus_1_isolate_pi/TR/219F	2014	Turkey	Class II	VI	VI.2.1.1.2.2
1023	MN830310.1	UNVERIFIED:_Pigeon_paramyxovirus_1_isolate_pi/TR/220F	2014	Turkey	Class II	VI	VI.2.1.1.2.2
1024	MN830311.1	UNVERIFIED:_Pigeon_paramyxovirus_1_isolate_pi/TR/222F	2014	Turkey	Class II	VI	VI.2.1.1.2.2
1025	MN862497.1	Pigeon_paramyxovirus_1_strain_Pigeon/Beijing/BJ96/1996	1996	China	Class II	VI	VI.2.2.2

1026	MN862498.1	Pigeon_paramyxovirus_1_strain_Pigeon/Shandong/JN08/2008	2008	China	Class II	VII	VII.1.1
1027	MN862499.1	Pigeon_paramyxovirus_1_strain_Pigeon/Shandong/TA14/2014	2014	China	Class II	VI	VI.2.1.1.2.1
1028	MN862500.1	Pigeon_paramyxovirus_1_strain_Pigeon/Shandong/PP09/2009	2009	China	Class II	VI	VI.2.2.2
1029	MN862501.1	Pigeon_paramyxovirus_1_strain_Pigeon/Shandong/TA13/2013	2013	China	Class II	VI	VI.2.1.1.2.1
1030	MN862502.1	Pigeon_paramyxovirus_1_strain_Pigeon/Shandong/C9/2013	2013	China	Class II	VI	VI.2.2.2
1031	MN862503.1	Pigeon_paramyxovirus_1_strain_Pigeon/Shandong/LCE4/2016	2016	China	Class II	VI	VI.2.2.2
1032	MN862504.1	Pigeon_paramyxovirus_1_strain_Pigeon/Shandong/LC/2017	2017	China	Class II	VI	VI.2.2.2
1033	MN862505.1	Pigeon_paramyxovirus_1_strain_Pigeon/Shandong/3P3/2018	2018	China	Class II	VI	VI.2.1.1.2.2
1034	MN862506.1	Pigeon_paramyxovirus_1_strain_Pigeon/Shandong/190610-2/2019	2019	China	Class II	VI	VI.2.1.1.2.2
1035	MN893303.1	Avian_orthoavulavirus_1_strain_pigeon/Guangdong/GZ08/2017	2017	China	Class II	VI	VI.2.1.1.2.2
1036	MN893304.1	Avian_orthoavulavirus_1_strain_pigeon/Guangdong/SZ12/2018	2018	China	Class II	VI	VI.2.1.1.2.2
1037	MN893305.1	Avian_orthoavulavirus_1_strain_pigeon/Guangdong/HY25/2019	2019	China	Class II	I	I.1.1
1038	MN953614.1	Avian_orthoavulavirus_1_strain_pigeon/Jiangsu/2453/2018	2018	China	Class II	VI	VI.2.1.1.2.2
1039	MN953615.1	Avian_orthoavulavirus_1_strain_pigeon/Ningxia/2364/2018	2018	China	Class II	VI	VI.2.1.1.2.2
1040	MN953616.1	Avian_orthoavulavirus_1_strain_pigeon/Jiangsu/1427/2019	2019	China	Class II	VI	VI.2.1.1.2.2
1041	MN953617.1	Avian_orthoavulavirus_1_strain_pigeon/Shanghai/1024/2019	2019	China	Class II	VI	VI.2.1.1.2.2
1042	MN953618.1	Avian_orthoavulavirus_1_strain_pigeon/Sichuan/1433/2019	2019	China	Class II	VI	VI.2.1.1.2.2
1043	MT081321.1	Avian_orthoavulavirus_1_isolate_KZ/Zhambyl/32/2014	2014	Zhambyl	Class II	XXI	XXI.1.1
1044	MT108196.1	Avian_orthoavulavirus_1_isolate_AOAV-1/pigeon/Eg/Q/F-VI/2009	2009	Egypt	Class II	XXI	XXI.1.2
1045	MT120189.1	Avian_orthoavulavirus_1_isolate_8_precursor	2011	China	Class II	VI	VI.2.2.2
1046	MT120191.1	Avian_orthoavulavirus_1_isolate_10_precursor	2009	China	Class II	VI	VI.2.2.2
1047	MT120200.1	Avian_orthoavulavirus_1_isolate_34_precursor	2018	China	Class II	II	II
1048	MT155812.1	Avian_orthoavulavirus_1_isolate_Pigeon/Philippines/2017/RPG17KVG10	2017	Philippines	Class II	VI	VI.2.1.1.1
1049	MT161597.1	Avian_orthoavulavirus_1_strain_pi/CH/GXP22/2000	2000	China	Class II	VI	VI.2.1.1.2.1
1050	MT161598.1	Avian_orthoavulavirus_1_strain_pi/CH/GX0019/2012	2012	China	Class II	VI	VI.2.1.1.2.2
1051	MT274648.1	Avian_orthoavulavirus_1_isolate_108/MIB/2014	2014	India	Class II	XXI	XXI.1.2

1052	MT362716.1	Avian_orthoavulavirus_1_isolate_P1	2017	India	Class II	XIII	XIII.2.2
1053	MT487719.1	Pigeon_paramyxovirus_1_isolate_pigeon/Iran/UT_EGV_1/2018	2018	Iran	Class II	XXI	XXI.1.1
1054	MT487720.1	Pigeon_paramyxovirus_1_isolate_pigeon/Iran/UT_EGV_2/2018	2018	Iran	Class II	XXI	XXI.1.1
1055	MT487721.1	Pigeon_paramyxovirus_1_isolate_pigeon/Iran/UT_EGV_3/2018	2018	Iran	Class II	XXI	XXI.1.1
1056	MT487722.1	Pigeon_paramyxovirus_1_isolate_pigeon/Iran/UT_EGV_4/2018	2018	Iran	Class II	XXI	XXI.1.2
1057	MT487723.1	Pigeon_paramyxovirus_1_isolate_pigeon/Iran/UT_EGV_5/2018	2018	Iran	Class II	XXI	XXI.1.1
1058	MT487724.1	Pigeon_paramyxovirus_1_isolate_pigeon/Iran/UT_EGV_6/2018	2018	Iran	Class II	XXI	XXI.1.1
1059	MT487725.1	Pigeon_paramyxovirus_1_isolate_pigeon/Iran/UT_EGV_7/2018	2018	Iran	Class II	XXI	XXI.1.1
1060	MT487726.1	Pigeon_paramyxovirus_1_isolate_pigeon/Iran/UT_EGV_8/2018	2018	Iran	Class II	XXI	XXI.1.1
1061	MT487727.1	Pigeon_paramyxovirus_1_isolate_pigeon/Iran/UT_EGV_9/2018	2018	Iran	Class II	XXI	XXI.1.1
1062	MT487728.1	Pigeon_paramyxovirus_1_isolate_pigeon/Iran/UT_EGV_10/2018	2018	Iran	Class II	XXI	XXI.1.1
1063	MT524978.1	Avian_orthoavulavirus_1_isolate_NDV/pigeon/Bangladesh/P13C/2019	2019	Bangladesh	Class II	XX	XX
1064	MT524979.1	Avian_orthoavulavirus_1_isolate_NDV/pigeon/Bangladesh/P11C/2019	2019	Bangladesh	Class II	XXI	XXI.1.2
1065	MT524980.1	Avian_orthoavulavirus_1_isolate_NDV/pigeon/Bangladesh/P14C/2019	2019	Bangladesh	Class II	XXI	XXI.1.2
1066	MT524981.1	Avian_orthoavulavirus_1_isolate_NDV/pigeon/Bangladesh/P21C/2019	2019	Bangladesh	Class II	XXI	XXI.1.2
1067	MT524982.1	Avian_orthoavulavirus_1_isolate_NDV/pigeon/Bangladesh/P32C/2019	2019	Bangladesh	Class II	XXI	XXI.1.2
1068	MT524983.1	Avian_orthoavulavirus_1_isolate_NDV/pigeon/Bangladesh/P34C/2019	2019	Bangladesh	Class II	XXI	XXI.1.2
1069	MT524984.1	Avian_orthoavulavirus_1_isolate_NDV/pigeon/Bangladesh/P37C/2019	2019	Bangladesh	Class II	XX	XX
1070	MT524985.1	Avian_orthoavulavirus_1_isolate_NDV/pigeon/Bangladesh/P46C/2019	2019	Bangladesh	Class II	XX	XX
1071	MT524986.1	Avian_orthoavulavirus_1_isolate_NDV/pigeon/Bangladesh/P56C/2019	2019	Bangladesh	Class II	XX	XX
1072	MT524987.1	Avian_orthoavulavirus_1_isolate_NDV/pigeon/Bangladesh/P57C/2019	2019	Bangladesh	Class II	XX	XX
1073	MT524988.1	Avian_orthoavulavirus_1_isolate_NDV/pigeon/Bangladesh/P63C/2019	2019	Bangladesh	Class II	XX	XX
1074	MT811571.1	Avian_orthoavulavirus_1_isolate_JS/03/11/Pi	2011	China	Class II	VI	VI.2.1.1.2.2
1075	MT811572.1	Avian_orthoavulavirus_1_isolate_ZJ/01/13/Pi	2013	China	Class II	VI	VI.2.1.1.2.2
1076	MT811573.1	Avian_orthoavulavirus_1_isolate_ZJ/02/13/Pi	2013	China	Class II	VI	VI.2.1.1.2.2
1077	MT811574.1	Avian_orthoavulavirus_1_isolate_ZJ/03/13/Pi	2013	China	Class II	VI	VI.2.1.1.2.2

1078	MT811576.1	Avian_orthoavulavirus_1_isolate_JS/03/15/Pi	2015	China	Class II	VI	VI.2.1.1.2.2
1079	MT811578.1	Avian_orthoavulavirus_1_isolate_JS/09/16/Pi	2016	China	Class II	VI	VI.2.1.1.2.2
1080	MT811579.1	Avian_orthoavulavirus_1_isolate_SD/10/16/Pi	2016	China	Class II	VI	VI.2.1.1.2.2
1081	MT811580.1	Avian_orthoavulavirus_1_isolate_JS/06/17/Pi	2017	China	Class II	VI	VI.2.1.1.2.2
1082	MT811582.1	Avian_orthoavulavirus_1_isolate_JS/05/19/Pi	2019	China	Class II	VI	VI.2.1.1.2.2
1083	MT811583.1	Avian_orthoavulavirus_1_isolate_JS/06/19/Pi	2019	China	Class II	VI	VI.2.1.1.2.2
1084	MT811584.1	Avian_orthoavulavirus_1_isolate_AH/01/19/Pi	2019	China	Class II	VI	VI.2.1.1.2.2
1085	MT811585.1	Avian_orthoavulavirus_1_isolate_AH/02/19/Pi	2019	China	Class II	VI	VI.2.1.1.2.2
1086	MT811586.1	Avian_orthoavulavirus_1_isolate_SD/09/19/Pi	2019	China	Class II	VI	VI.2.1.1.2.2
1087	MT811587.1	Avian_orthoavulavirus_1_isolate_JS/12/19/Pi	2019	China	Class II	VI	VI.2.1.1.2.2
1088	MT811588.1	Avian_orthoavulavirus_1_isolate_JS/13/19/Pi	2019	China	Class II	VI	VI.2.1.1.2.2
1089	MT920212.1	Avian_orthoavulavirus_1_isolate_Pigeon/Pakistan/Lahore/AW-1021/2019	2019	Pakistan	Class II	XX	XX
1090	MT920213.1	Avian_orthoavulavirus_1_isolate_Pigeon/Pakistan/Lahore/AW-1022/2019	2019	Pakistan	Class II	XX	XX
1091	MT920214.1	Avian_orthoavulavirus_1_isolate_Pigeon/Pakistan/Lahore/AW-1023/2019	2019	Pakistan	Class II	XX	XX
1092	MW147625.1	Avian_orthoavulavirus_1_strain_pigeon/China/TJ2017	2017	China	Class II	VI	VI.2.1.1.2.2
1093	MW147626.1	Avian_orthoavulavirus_1_strain_pigeon/China/BJ2018	2018	China	Class II	VI	VI.2.1.1.2.2
1094	MW271783.1	Avian_orthoavulavirus_1_isolate_JS/23/11/Pi	2011	China	Class II	VI	VI.2.1.1.2.2
1095	MW271784.1	Avian_orthoavulavirus_1_isolate_ZJ_03_13_Pi	2013	China	Class II	VI	VI.2.1.1.2.2
1096	MW271785.1	Avian_orthoavulavirus_1_isolate_JS/01/15/Pi	2015	China	Class II	VI	VI.2.1.1.2.2
1097	MW271786.1	Avian_orthoavulavirus_1_isolate_AH/06/16/Pi	2016	China	Class II	VI	VI.2.1.1.2.2
1098	MW271788.1	Avian_orthoavulavirus_1_isolate_GS/12/17/Pi	2017	China	Class II	VI	VI.2.1.1.2.2
1099	MW271789.1	Avian_orthoavulavirus_1_isolate_AH/01/20/Pi	2020	China	Class II	VI	VI.2.1.1.2.2
1100	MW271790.1	Avian_orthoavulavirus_1_isolate_JS/01/20/Pi	2020	China	Class II	VI	VI.2.1.1.2.2
1101	MW271791.1	Avian_orthoavulavirus_1_isolate_JS/06/20/Pi	2020	China	Class II	VI	VI.2.1.1.2.2
1102	MW271792.1	Avian_orthoavulavirus_1_isolate_JS/07/20/Pi	2020	China	Class II	VI	VI.2.1.1.2.2
1103	MW412840.1	Avian_orthoavulavirus_1_strain_VI-HZ	2017	China	Class II	VI	VI.2.2.2

1104	MZ029051.1	Avian_orthoavulavirus_1_isolate_NDV-Pigeon-Sharkia-ZAG.UNI-9-2020	2020	USA	Class II	VII	VII.1.1
1105	MZ029053.1	Avian_orthoavulavirus_1_isolate_NDV/Pigeon/Egypt/Cairo-ZAG.UNI-19/2019	2019	Egypt	Class II	XVIII	XVIII.2
1106	MZ101341.1	Avian_orthoavulavirus_1_isolate_pigeon/Ukraine/Kharkiv/Kuksov/13-05/2014	2014	Ukraine	Class II	XXI	XXI.1.1
1107	MZ101342.1	Avian_orthoavulavirus_1_isolate_pigeon/Ukraine/Kharkiv/Petruk/15-01/2015	2015	Ukraine	Class II	VI	VI.2.1.1.2.2
1108	MZ101344.1	Avian_orthoavulavirus_1_isolate_pigeon/Ukraine/Doneck/10/26-6/2008	2008	Ukraine	Class II	II	II
1109	MZ223436.1	Avian_orthoavulavirus_1_isolate_JS/02/20/Pi	2020	China	Class II	VI	VI.2.1.1.2.2
1110	MZ223437.1	Avian_orthoavulavirus_1_isolate_HN/01/20/Pi	2020	China	Class II	VI	VI.2.1.1.2.2
1111	MZ223438.1	Avian_orthoavulavirus_1_isolate_HN/02/20/Pi	2020	China	Class II	VI	VI.2.1.1.2.2
1112	MZ277620.1	Avian_orthoavulavirus_1_isolate_Pigeon/Tianjin/0044/2019	2019	China	Class II	VI	VI.2.1.1.2.2
1113	MZ277621.1	Avian_orthoavulavirus_1_isolate_Pigeon/Tianjin/0054/2019	2019	China	Class II	VI	VI.2.1.1.2.2
1114	MZ277622.1	Avian_orthoavulavirus_1_isolate_Pigeon/Beijing/0072/2019	2019	China	Class II	VI	VI.2.1.1.2.2
1115	MZ277623.1	Avian_orthoavulavirus_1_isolate_Pigeon/China/su/2019	2019	China	Class II	VI	VI.2.1.1.2.1
1116	MZ306211.1	Avian_orthoavulavirus_1_strain_pigeon/China/GXD12/2000	2000	China	Class II	I	I.1.1
1117	MZ306219.1	Avian_orthoavulavirus_1_strain_pigeon/China/GXD27/2018	2018	China	Class II	VI	VI.2.1.1.2.1
1118	MZ306222.1	Avian_orthoavulavirus_1_strain_Turtle_dove/China/GX11/2018	2018	China	Class II	VI	VI.2.1.1.2.2
1119	MZ363634.1	Avian_orthoavulavirus_1_strain_Pigeon/China/GX03/2017	2017	China	Class II	VI	VI.2.1.1.2.2
1120	MZ395248.1	Avian_orthoavulavirus_1_strain_Pigeon/China/GX04/2016	2016	China	Class II	VI	VI.2.1.1.2.2
1121	MZ395249.1	Avian_orthoavulavirus_1_strain_Turtle_dove/China/GX05/2017	2017	China	Class II	VI	VI.2.1.1.2.2
1122	MZ395250.1	Avian_orthoavulavirus_1_strain_Turtle_dove/China/GX06/2016	2016	China	Class II	VI	VI.2.1.1.2.2
1123	MZ395251.1	Avian_orthoavulavirus_1_strain_Pigeon/China/GX07/2017	2017	China	Class II	VI	VI.2.1.1.2.2
1124	MZ400779.1	Avian_orthoavulavirus_1_strain_Turtle_dove/China/GX08/2016	2016	China	Class II	VI	VI.2.1.1.2.2
1125	MZ400780.1	Avian_orthoavulavirus_1_strain_Turtle_dove/China/GX09/2016	2016	China	Class II	VI	VI.2.1.1.2.2
1126	MZ405131.1	Avian_orthoavulavirus_1_strain_Spotted_dove/China/GX07/2019	2019	China	Class II	VI	VI.2.1.1.2.2
1127	MZ405134.1	Avian_orthoavulavirus_1_strain_pigeon/China/GXD17/2006	2006	China	Class II	I	I.1.1
1128	MZ405135.1	Avian_orthoavulavirus_1_strain_pigeon/China/GXD31/2016	2016	China	Class II	VI	VI.2.1.1.2.1
1129	MZ405137.1	Avian_orthoavulavirus_1_strain_pigeon/China/GXD28/2020	2020	China	Class II	VI	VI.2.1.1.2.2

1130	MZ409510.1	Avian_orthoavulavirus_1_strain_VI-NJ	2006	China	Class II	VI	VI.2.1.1.2.1
1131	MZ458602.1	Avian_orthoavulavirus_1_isolate_NDV/pigeon/SP-Brazil/CCZ002/2019	2019	Brazil	Class II	VI	VI.2.1.2
1132	MZ620686.1	Avian_orthoavulavirus_1_strain_Spotted_dove/China/GX08/2017	2017	China	Class II	VI	VI.2.1.1.2.2
1133	MZ620687.1	Avian_orthoavulavirus_1_strain_Spotted_dove/China/GX09/2017	2017	China	Class II	VI	VI.2.1.1.2.2
1134	MZ620688.1	Avian_orthoavulavirus_1_strain_Turtle_dove/China/GX10/2018	2018	China	Class II	VI	VI.2.1.1.2.2
1135	MZ620689.1	Avian_orthoavulavirus_1_strain_Pigeon/China/GX12/2019	2019	China	Class II	VI	VI.2.1.1.2.1
1136	MZ620690.1	Avian_orthoavulavirus_1_strain_Spotted_dove/China/GX15/2018	2018	China	Class II	VI	VI.2.1.1.2.2
1137	MZ620693.1	Avian_orthoavulavirus_1_strain_Turtle_dove/China/GX16/2018	2018	China	Class II	VI	VI.2.1.1.2.2
1138	MZ620694.1	Avian_orthoavulavirus_1_strain_Turtle_dove/China/GX17/2018	2018	China	Class II	VI	VI.2.1.1.2.2
1139	MZ620696.1	Avian_orthoavulavirus_1_strain_Spotted_dove/China/GX06/2019	2019	China	Class II	VI	VI.2.1.1.2.2
1140	MZ620698.1	Avian_orthoavulavirus_1_strain_Turtle_dove/China/GX11/2019	2019	China	Class II	VI	VI.2.1.1.2.2
1141	MZ736924.1	Newcastle_disease_virus_class_1_isolate_PI/CH/LFJ/277/2017	2017	China	Class I	1	1.1.2
1142	MZ737065.1	Newcastle_disease_virus_class_1_isolate_PI/CH/LNX/38/2018	2018	China	Class I	1	1.1.2
1143	MZ737079.1	Newcastle_disease_virus_class_1_isolate_PI/CH/LSC/309/2018	2018	China	Class I	1	1.1.2
1144	MZ737087.1	Newcastle_disease_virus_class_1_isolate_PI/CH/LSH/101/2018	2018	China	Class I	1	1.1.2
1145	MZ802821.1	Avian_orthoavulavirus_1_isolate_APMV-1/dove/Taiwan/AHRI144/2019	2019	China	Class II	VI	VI.2.1.1.2.2
1146	MZ802822.1	Avian_orthoavulavirus_1_isolate_APMV-1/pigeon/Taiwan/AHRI147/2019	2019	China	Class II	VI	VI.2.1.1.2.2
1147	MZ928457.1	Avian_orthoavulavirus_1_isolate_NDV/Pigeon/Sudan/Khartoum-A19/2017	2017	Sudan	Class II	XXI	XXI.1.1
1148	OK135954.1	Avian_orthoavulavirus_1_isolate_NDV/Pigeon/Sudan/Khartoum-A2/	2017	Sudan	Class II	VII	VII.1.1
1149	OL513713.1	Avian_orthoavulavirus_1_isolate_pigeon/Shanghai/2095/2017	2017	China	Class I	1	1.1.2
1150	OL513763.1	Avian_orthoavulavirus_1_isolate_pigeon/Jiangxi/2360/2017	2017	China	Class I	1	1.1.2
1151	OL513985.1	Avian_orthoavulavirus_1_isolate_pigeon/Jiangxi/1027/2018	2018	China	Class I	1	1.1.2
1152	OL513986.1	Avian_orthoavulavirus_1_isolate_pigeon/Jiangxi/1419/2018	2018	China	Class I	1	1.1.2
1153	OL518991.1	Avian_orthoavulavirus_1_isolate_APMV-1/Rock_Dove/NYC/USA/NYCVH/21-0109/2021	2021	USA	Class II	VI	VI.2.1.1.1
1154	OL518992.1	Avian_orthoavulavirus_1_isolate_APMV-1/Rock_Dove/NYC/USA/NYCVH/21-0052/2021	2021	USA	Class II	VI	VI.2.1.1.1
1155	OL547962.1	Avian_orthoavulavirus_1_strain_Pigeon/Jiangsu/1482/2011	2011	China	Class I	1	1.1.2

1156	OL548139.1	Avian_orthoavulavirus_1_strain_Pigeon/Shanxi/2054/2012	2012	China	Class I	1	1.1.2
1157	OL548140.1	Avian_orthoavulavirus_1_strain_Pigeon/Shanxi/2081/2012	2012	China	Class I	1	1.1.2
1158	OL548141.1	Avian_orthoavulavirus_1_strain_Pigeon/Shanxi/2082/2012	2012	China	Class I	1	1.1.2
1159	OL548142.1	Avian_orthoavulavirus_1_strain_Pigeon/Shanxi/2088/2012	2012	China	Class I	1	1.1.2
1160	OL548143.1	Avian_orthoavulavirus_1_strain_Pigeon/Shanxi/2122/2012	2012	China	Class I	1	1.1.2
1161	OL548144.1	Avian_orthoavulavirus_1_strain_Pigeon/Shanxi/2125/2012	2012	China	Class I	1	1.1.2
1162	OL548145.1	Avian_orthoavulavirus_1_strain_Pigeon/Shanxi/2187/2012	2012	China	Class I	1	1.1.2
1163	OL548146.1	Avian_orthoavulavirus_1_strain_Pigeon/Shanxi/2188/2012	2012	China	Class I	1	1.1.2
1164	OL548164.1	Avian_orthoavulavirus_1_strain_Pigeon/Anhui/1151/2013	2013	China	Class I	1	1.1.2
1165	OL548165.1	Avian_orthoavulavirus_1_strain_Pigeon/Anhui/1155/2013	2013	China	Class I	1	1.1.2
1166	OL548166.1	Avian_orthoavulavirus_1_strain_Pigeon/Anhui/2195/2013	2013	China	Class I	1	1.1.2
1167	OL548167.1	Avian_orthoavulavirus_1_strain_Pigeon_water/Anhui/2224/2013	2013	China	Class I	1	1.1.2
1168	OL548168.1	Avian_orthoavulavirus_1_strain_Pigeon_water/Anhui/2259/2013	2013	China	Class I	1	1.1.2
1169	OL548180.1	Avian_orthoavulavirus_1_strain_Pigeon/Guizhou/2038/2013	2013	China	Class I	1	1.1.2
1170	OL548205.1	Avian_orthoavulavirus_1_strain_Pigeon/Henan/2047/2013	2013	China	Class I	1	1.1.2
1171	OL548222.1	Avian_orthoavulavirus_1_strain_Pigeon/Hubei/2200/2013	2013	China	Class I	1	1.1.2
1172	OL548317.1	Avian_orthoavulavirus_1_strain_Pigeon/Sichuan/2155/2013	2013	China	Class I	1	1.1.2
1173	OL548365.1	Avian_orthoavulavirus_1_strain_Pigeon/Chongqing/2129/2013	2013	China	Class I	1	1.1.2
1174	OL548432.1	Avian_orthoavulavirus_1_strain_Pigeon/Guizhou/1044/2014	2014	China	Class I	1	1.1.2
1175	OL548679.1	Avian_orthoavulavirus_1_strain_Pigeon/Shanghai/1056/2014	2014	China	Class I	1	1.1.2
1176	OL548829.1	Avian_orthoavulavirus_1_strain_Pigeon/Shanghai/3855/2014	2014	China	Class I	1	1.1.2
1177	OL580885.1	Avian_orthoavulavirus_1_isolate_pigeon/Jiangxi/2399/2019	2019	China	Class I	1	1.1.2
1178	OL580912.1	Avian_orthoavulavirus_1_isolate_pigeon/Jiangxi/2431/2019	2019	China	Class I	1	1.1.2
1179	OL581424.1	Avian_orthoavulavirus_1_isolate_pigeon/Jiangxi/2226/2020	2020	China	Class I	1	1.1.2
1180	OM258694.1	Avian_orthoavulavirus_1_strain_pigeon/Fujian/2121/2020	2020	China	Class II	VI	VI.2.1.1.2.2
1181	OM258695.1	Avian_orthoavulavirus_1_strain_pigeon/Jiangxi/2176/2020	2020	China	Class II	VI	VI.2.1.1.2.2

1182	OM258696.1	Avian_orthoavulavirus_1_strain_pigeon/Anhui/1350/2021	2021	China	Class II	VI	VI.2.1.1.2.2
1183	OM258697.1	Avian_orthoavulavirus_1_strain_pigeon/Fujian/1394/2021	2019	China	Class II	VI	VI.2.1.1.2.2
1184	OM418380.1	Avian_orthoavulavirus_1_isolate_MP003	2018	Brazil	Class II	VI	VI.2.1.2
1185	OM522660.1	Avian_orthoavulavirus_1_isolate_pigeon/Pakistan/Rawalpindi/2015	2015	Pakistan	Class II	XX	XX
1186	OM640464.1	Pigeon_paramyxovirus_1_strain_PPMV-1/pigeon/Gansu/China/02/2020	2020	China	Class II	VI	VI.2.1.1.2.2
1187	ON512717.1	Pigeon_paramyxovirus_1_isolate_D1705329	2017	USA	Class II	VI	VI.2.1.1.1
1188	ON512718.1	Pigeon_paramyxovirus_1_isolate_K1300482	2013	USA	Class II	VI	VI.2.1.1.1
1189	ON512719.1	Pigeon_paramyxovirus_1_isolate_K1400434	2014	USA	Class II	VI	VI.2.1.1.1
1190	ON512720.1	Pigeon_paramyxovirus_1_isolate_K1401255	2014	USA	Class II	VI	VI.2.1.1.1
1191	ON512721.1	Pigeon_paramyxovirus_1_isolate_K1401933	2014	USA	Class II	VI	VI.2.1.1.1
1192	ON512722.1	Pigeon_paramyxovirus_1_isolate_K1501131	2015	USA	Class II	VI	VI.2.1.1.1
1193	ON512723.1	Pigeon_paramyxovirus_1_isolate_K1501454	2015	USA	Class II	VI	VI.2.1.1.1
1194	ON512724.1	Pigeon_paramyxovirus_1_isolate_K1600456	2016	USA	Class II	VI	VI.2.1.1.1
1195	ON512725.1	Pigeon_paramyxovirus_1_isolate_K1600553	2016	USA	Class II	VI	VI.2.1.1.1
1196	ON512726.1	Pigeon_paramyxovirus_1_isolate_K1600644	2016	USA	Class II	VI	VI.2.1.1.1
1197	ON512727.1	Pigeon_paramyxovirus_1_isolate_K1600859	2016	USA	Class II	VI	VI.2.1.1.1
1198	ON512728.1	Pigeon_paramyxovirus_1_isolate_K1602689	2016	USA	Class II	VI	VI.2.1.1.1
1199	ON512729.1	Pigeon_paramyxovirus_1_isolate_K1603086	2016	USA	Class II	VI	VI.2.1.1.1
1200	ON512730.1	Pigeon_paramyxovirus_1_isolate_K1700451	2017	USA	Class II	VI	VI.2.1.1.1
1201	ON512731.1	Pigeon_paramyxovirus_1_isolate_K1700703	2017	USA	Class II	VI	VI.2.1.1.1
1202	ON512732.1	Pigeon_paramyxovirus_1_isolate_K1700817	2017	USA	Class II	VI	VI.2.1.1.1
1203	ON512733.1	Pigeon_paramyxovirus_1_isolate_K1700945	2017	USA	Class II	VI	VI.2.1.1.1
1204	ON512734.1	Pigeon_paramyxovirus_1_isolate_K1700972	2017	USA	Class II	VI	VI.2.1.1.1
1205	ON512735.1	Pigeon_paramyxovirus_1_isolate_K1701944	2017	USA	Class II	VI	VI.2.1.1.1
1206	ON512736.1	Pigeon_paramyxovirus_1_isolate_K1702213	2017	USA	Class II	VI	VI.2.1.1.1
1207	ON512737.1	Pigeon_paramyxovirus_1_isolate_K1702361	2017	USA	Class II	VI	VI.2.1.1.1

1208	ON512738.1	Pigeon_paramyxovirus_1_isolate_K1702469	2017	USA	Class II	VI	VI.2.1.1.1
1209	ON512739.1	Pigeon_paramyxovirus_1_isolate_K1801328	2018	USA	Class II	VI	VI.2.1.1.1
1210	ON512740.1	Pigeon_paramyxovirus_1_isolate_K1801329	2018	USA	Class II	VI	VI.2.1.1.1
1211	ON512741.1	Pigeon_paramyxovirus_1_isolate_K1801495	2018	USA	Class II	VI	VI.2.1.1.1
1212	ON512742.1	Pigeon_paramyxovirus_1_isolate_K1802082	2018	USA	Class II	VI	VI.2.1.1.1
1213	ON512743.1	Pigeon_paramyxovirus_1_isolate_S1402687	2014	USA	Class II	VI	VI.2.1.1.1
1214	ON512744.1	Pigeon_paramyxovirus_1_isolate_S1501211	2015	USA	Class II	VI	VI.2.1.1.1
1215	ON512745.1	Pigeon_paramyxovirus_1_isolate_S1700135	2017	USA	Class II	VI	VI.2.1.1.1
1216	ON512746.1	Pigeon_paramyxovirus_1_isolate_S1703718	2017	USA	Class II	VI	VI.2.1.1.1
1217	ON512747.1	Pigeon_paramyxovirus_1_isolate_S1708149	2017	USA	Class II	VI	VI.2.1.1.1
1218	ON512748.1	Pigeon_paramyxovirus_1_isolate_S2002105	2020	USA	Class II	VI	VI.2.1.1.1
1219	ON512749.1	Pigeon_paramyxovirus_1_isolate_T1501391	2015	USA	Class II	VI	VI.2.1.1.1
1220	ON512750.1	Pigeon_paramyxovirus_1_isolate_T1600201	2016	USA	Class II	VI	VI.2.1.1.1
1221	ON512751.1	Pigeon_paramyxovirus_1_isolate_T1800802	2018	USA	Class II	VI	VI.2.1.1.1
1222	ON637881.1	Pigeon_paramyxovirus_1_strain_NTU/C239/21	2021	China	Class II	VI	VI.2.1.1.2.2
1223	ON645962.1	Avian_orthoavulavirus_1_isolate_AOAV-1/pigeon/Macedonia/230/2008	2008	Macedonia	Class II	VI	VI.2.1.1.2.2
1224	ON645964.1	Avian_orthoavulavirus_1_isolate_AOAV-1/pigeon/Macedonia/232/2008	2008	Macedonia	Class II	VI	VI.2.1.1.2.2
1225	ON645965.1	Avian_orthoavulavirus_1_isolate_AOAV-1/pigeon/Macedonia/234/2007	2007	Macedonia	Class II	VI	VI.2.1.1.2.2
1226	ON645966.1	Avian_orthoavulavirus_1_isolate_AOAV-1/pigeon/Macedonia/415/2011	2011	Macedonia	Class II	VI	VI.2.1.1.2.2
1227	ON645967.1	Avian_orthoavulavirus_1_isolate_AOAV-1/pigeon/Macedonia/419/2011	2011	Macedonia	Class II	VI	VI.2.1.1.2.2
1228	ON645968.1	Avian_orthoavulavirus_1_isolate_AOAV-1/feralpigeon/Macedonia/9701/2012	2012	Macedonia	Class II	VI	VI.2.1.1.2.2
1229	ON645969.1	Avian_orthoavulavirus_1_isolate_AOAV-1/pigeon/Macedonia/1503/2007	2007	Macedonia	Class II	VI	VI.2.1.1.2.2
1230	ON645970.1	Avian_orthoavulavirus_1_isolate_AOAV-1/pigeon/Macedonia/962/2008	2008	Macedonia	Class II	VI	VI.2.1.1.2.2
1231	ON645971.1	Avian_orthoavulavirus_1_isolate_AOAV-1/pigeon/Macedonia/1501/2007	2007	Macedonia	Class II	VI	VI.2.1.1.2.2
1232	ON645972.1	Avian_orthoavulavirus_1_isolate_AOAV-1/pigeon/Macedonia/2810/2010	2010	Macedonia	Class II	VI	VI.2.1.1.2.2
1233	ON918567.1	Avian_orthoavulavirus_1_strain_NDV/P1/22/RSP-2	2022	India	Class II	II	II

1234	ON918568.1	Avian_orthoavulavirus_1_strain_NDV/P2/22/RSP-2	2022	India	Class II	II	II
1235	ON918569.1	Avian_orthoavulavirus_1_strain_NDV/P3/22/RSP-2	2022	India	Class II	II	II
1236	ON939087.1	Pigeon_paramyxovirus_1_strain_Pigeon/HeBei/210818	2021	China	Class II	VI	VI.2.1.1.2.2
1237	ON986202.1	Avian_orthoavulavirus_1_isolate_PPMV-1/pigeon/Switzerl/P147-ND52/2021	2021	Switzerl	Class II	VI	VI.2.1.1.2.2
1238	ON986203.1	Avian_orthoavulavirus_1_isolate_PPMV-1/pigeon/Switzerl/P228-ND53/2021	2021	Switzerl	Class II	VI	VI.2.1.1.2.2
1239	ON986204.1	Avian_orthoavulavirus_1_isolate_PPMV-1/pigeon/Switzerl/P297-ND54/2021	2021	Switzerl	Class II	VI	VI.2.1.1.2.2
1240	ON986205.1	Avian_orthoavulavirus_1_isolate_PPMV-1/pigeon/Switzerl/P382-ND55/2021	2021	Switzerl	Class II	VI	VI.2.1.1.2.2
1241	ON986206.1	Avian_orthoavulavirus_1_isolate_PPMV-1/pigeon/Switzerl/V051-1/2022	2022	Switzerl	Class II	VI	VI.2.1.1.2.2
1242	OP221244.1	Pigeon_paramyxovirus_1_isolate_Pigeon/Egypt/Qalyubia/Kb1/2020	2020	Egypt	Class II	XXI	XXI.1.1
1243	OP535454.1	Avian_orthoavulavirus_1_isolate_pi/Pak/Lhr/XXI.1.1/SA_1/2017	2017	Pakistan	Class II	XXI	XXI.1.1
1244	OP535455.1	Avian_orthoavulavirus_1_isolate_pi/Pak/Lhr/XXI.1.2/SA_2/2017	2017	Pakistan	Class II	XX	XX
1245	OP641847.1	Avian_orthoavulavirus_1_isolate_APMV-1/Pigeon/Bangladesh/SG-47/2020	2020	Bangladesh	Class II	XX	XX
1246	OP641848.1	Avian_orthoavulavirus_1_isolate_APMV-1/Pigeon/Bangladesh/LT-43/2020	2020	Bangladesh	Class II	XX	XX
1247	OP641849.1	Avian_orthoavulavirus_1_isolate_APMV-1/Pigeon/Bangladesh/SG-57/2021	2021	Bangladesh	Class II	XX	XX
1248	OP751936.1	Avian_orthoavulavirus_1_isolate_pigeon/China/WZ2201/2022	2022	China	Class II	VI	VI.2.1.1.2.2
1249	OP796698.1	Avian_orthoavulavirus_1_isolate_pigeon/China/WZ2205/2022	2022	China	Class II	VI	VI.2.1.1.2.2
1250	OQ144642.1	Avian_orthoavulavirus_1_isolate_pigeon/China/Anhui/2432/2021	2019	China	Class II	VI	VI.2.1.1.2.1
1251	OQ144643.1	Avian_orthoavulavirus_1_isolate_pigeon/China/Fujian/2168/2021	2019	China	Class II	VI	VI.2.1.1.2.1
1252	OQ144644.1	Avian_orthoavulavirus_1_isolate_pigeon/China/Fujian/1187/2021	2019	China	Class II	VI	VI.2.1.1.2.2
1253	OQ144645.1	Avian_orthoavulavirus_1_isolate_pigeon/China/Jiangxi/2027/2021	2019	China	Class II	VI	VI.2.1.1.2.1
1254	OQ144646.1	Avian_orthoavulavirus_1_isolate_pigeon/China/Yunnan/1078/2022	2022	China	Class II	VI	VI.2.1.1.2.2
1255	OQ144647.1	Avian_orthoavulavirus_1_isolate_pigeon/China/Ningxia/1111/2022	2022	China	Class II	VI	VI.2.1.1.2.2
1256	OQ144648.1	Avian_orthoavulavirus_1_isolate_pigeon/China/Guangxi/1228/2022	2022	China	Class II	VI	VI.2.1.1.2.2
1257	OQ144649.1	Avian_orthoavulavirus_1_isolate_pigeon/China/Guangdong/2285/2022	2022	China	Class II	VI	VI.2.1.1.2.1
1258	OQ295860.1	Avian_orthoavulavirus_1_isolate_dove/Pak/Bal/WECD-121/2020	2020	Pakistan	Class II	XX	XX
1259	OQ295861.1	Avian_orthoavulavirus_1_isolate_dove/Pak/Bal/WECD-122/2020	2020	Pakistan	Class II	XX	XX

1260	OQ407702.1	Avian_orthoavulavirus_1_isolate_Pigeon/Pak/Karachi/AW-2/2015	2015	Pakistan	Class II	XXI	XXI.1.1
1261	OQ407703.1	Avian_orthoavulavirus_1_isolate_Pigeon/Pak/Multan/5-126/2014	2014	Pakistan	Class II	XXI	XXI.1.1
1262	OQ407704.1	Avian_orthoavulavirus_1_isolate_Pigeon/Pak/Gujranwala/998/2015	2015	Pakistan	Class II	XXI	XXI.1.1
1263	OQ407705.1	Avian_orthoavulavirus_1_isolate_Pigeon/Pak/Buner/KPK/1002/2015	2015	Pakistan	Class II	XXI	XXI.1.1
1264	OQ407706.1	Avian_orthoavulavirus_1_isolate_Pigeon/Pak/Buner/KPK/1003/2015	2015	Pakistan	Class II	XXI	XXI.1.1
1265	OQ407707.1	Avian_orthoavulavirus_1_isolate_Pigeon/Pak/Peshawar/KPK/8/2016	2016	Pakistan	Class II	XXI	XXI.1.1
1266	OQ407708.1	Avian_orthoavulavirus_1_isolate_Pigeon/Pak/Lahore/AW-71/2020	2020	Pakistan	Class II	XX	XX
1267	OQ407709.1	Avian_orthoavulavirus_1_isolate_Pigeon/Pak/Lahore/AW-72/2020	2020	Pakistan	Class II	XXI	XXI.1.2
1268	OQ407710.1	Avian_orthoavulavirus_1_isolate_Pigeon/Pak/Lahore/AW-73/2020	2020	Pakistan	Class II	XXI	XXI.1.2
1269	OQ407711.1	Avian_orthoavulavirus_1_isolate_Pigeon/Pak/Lahore/139/2015	2015	Pakistan	Class II	XX	XX
1270	OQ407712.1	Avian_orthoavulavirus_1_isolate_Pigeon/Pak/Lahore/123/2015	2015	Pakistan	Class II	XX	XX
1271	OQ407713.1	Avian_orthoavulavirus_1_isolate_Pigeon/Pak/Lahore/147/2015	2015	Pakistan	Class II	XX	XX
1272	OQ407714.1	Avian_orthoavulavirus_1_isolate_Pigeon/Pak/Pathoki/129/2017	2017	Pakistan	Class II	XX	XX
1273	OQ407715.1	Avian_orthoavulavirus_1_isolate_Pigeon/Pak/R-Pindi/SFR-17/2016	2016	Pakistan	Class II	XX	XX
1274	OQ407716.1	Avian_orthoavulavirus_1_isolate_Pigeon/Pak/Mirpur/AW-p55/2017	2017	Pakistan	Class II	XX	XX
1275	OQ407717.1	Avian_orthoavulavirus_1_isolate_Pigeon/Pak/Mirpur/AW-p56/2017	2017	Pakistan	Class II	XX	XX
1276	OQ407718.1	Avian_orthoavulavirus_1_isolate_Pigeon/Pak/Bhimber/AW-p57/2018	2018	Pakistan	Class II	XX	XX
1277	OQ407719.1	Avian_orthoavulavirus_1_isolate_Pigeon/Pak/Bhimber/AW-p58/2018	2018	Pakistan	Class II	XX	XX
1278	OQ407720.1	Avian_orthoavulavirus_1_isolate_Pigeon/Pak/Kotli/AW-p60/2018	2018	Pakistan	Class II	XX	XX
1279	OQ407721.1	Avian_orthoavulavirus_1_isolate_Pigeon/Pak/Kotli/AW-p62/2018	2018	Pakistan	Class II	XX	XX
1280	OQ407722.1	Avian_orthoavulavirus_1_isolate_Pigeon/Pak/Kotli/AW-p63/2018	2018	Pakistan	Class II	XX	XX
1281	OQ407723.1	Avian_orthoavulavirus_1_isolate_Pigeon/Pak/Pigeon/AW-b01/2019	2019	Pakistan	Class II	XX	XX
1282	OQ407724.1	Avian_orthoavulavirus_1_isolate_Pigeon/Pak/Pigeon/AW-b02/2020	2020	Pakistan	Class II	XX	XX
1283	OQ407725.1	Avian_orthoavulavirus_1_isolate_Pigeon/Pak/Badhana/1081/2015	2015	Pakistan	Class II	XX	XX
1284	OQ407726.1	Avian_orthoavulavirus_1_isolate_Pigeon/Pak/Chak_Shahzad/1001/2015	2015	Pakistan	Class II	XX	XX
1285	OQ407727.1	Avian_orthoavulavirus_1_isolate_Pigeon/Pakistan/Jhang/117/2017	2017	Pakistan	Class II	VII	VII.2

1286	OQ407728.1	Avian_orthoavulavirus_1_isolate_pigeon/Pakistan/Bhimber/AW-p59/2018	2018	Pakistan	Class II	VII	VII.2
1287	OQ407729.1	Avian_orthoavulavirus_1_isolate_pigeon/Pakistan/Badhana/998/2015	2015	Pakistan	Class II	VII	VII.2
1288	OQ435203.1	Avian_orthoavulavirus_1_isolate_Pi-Rus-Kostroma-3606-2017	2017	Russia	Class II	XXI	XXI.1.1
1289	OQ435204.1	Avian_orthoavulavirus_1_isolate_Pi-Rus-Vladimir-1141-2018	2018	Russia	Class II	XXI	XXI.1.1
1290	OQ435205.1	Avian_orthoavulavirus_1_isolate_Pi-Rus-Vladimir-1142-2018	2018	Russia	Class II	XXI	XXI.1.1
1291	OQ435206.1	Avian_orthoavulavirus_1_isolate_Pi-Rus-Tatarstan-311-1-2020	2020	Russia	Class II	XXI	XXI.1.1
1292	OQ435207.1	Avian_orthoavulavirus_1_isolate_Pi-Rus-Tatarstan-311-2-2020	2020	Russia	Class II	XXI	XXI.1.1
1293	OQ641418.1	Avian_orthoavulavirus_1_isolate_PI/CH/LAH/323/2022	2022	China	Class I	1	1.1.2
1294	OR085370.1	Avian_orthoavulavirus_1_strain_Pigeon/China/GX01/2019	2019	China	Class II	VI	VI.2.1.1.2.2
1295	OR085371.1	Avian_orthoavulavirus_1_strain_Pigeon/China/GX03/2019	2019	China	Class II	I	I.2
1296	OR085375.1	Avian_orthoavulavirus_1_strain_Pigeon/China/GX09/2019	2019	China	Class II	I	I.2
1297	OR099824.1	Avian_orthoavulavirus_1_strain_Turtle_dove/China/GX01/2018	2018	China	Class II	I	I.2
1298	OR099825.1	Avian_orthoavulavirus_1_strain_Pigeon/China/GX02/2019	2019	China	Class II	I	I.2
1299	OR099828.1	Avian_orthoavulavirus_1_strain_Pigeon/China/GX03/2018	2018	China	Class I	1	1.1.2
1300	OR233946.1	Avian_orthoavulavirus_1_isolate_186	2023	Israel	Class II	VI	VI.2.1.1.2.2
1301	OR233951.1	Avian_orthoavulavirus_1_isolate_12	2023	Israel	Class II	VII	VII.1.1
1302	OR233953.1	Avian_orthoavulavirus_1_isolate_78	2023	Israel	Class II	VI	VI.2.1.2
1303	OR253509.1	Avian_orthoavulavirus_1_isolate_APMV-1/pigeon/Heilongjiang/BS1/2018	2018	China	Class II	VI	VI.2.1.1.2.2
1304	OR253510.1	Avian_orthoavulavirus_1_isolate_APMV-1/pigeon/Heilongjiang/BS2/2018	2018	China	Class II	VI	VI.2.1.1.2.2
1305	OR260259.1	Avian_orthoavulavirus_1_isolate_74	2019	Israel	Class II	VI	VI.2.1.2
1306	OR364786.1	Avian_orthoavulavirus_1_isolate_88-2020	2020	Israel	Class II	XXI	XXI.2
1307	OR364787.1	Avian_orthoavulavirus_1_isolate_89-2020	2020	Israel	Class II	XXI	XXI.2
1308	OR364790.1	Avian_orthoavulavirus_1_isolate_205-2020	2020	Israel	Class II	VI	VI.2.1.2
1309	OR364799.1	Avian_orthoavulavirus_1_isolate_633-2020	2020	Israel	Class II	VI	VI.2.1.2
1310	OR364816.1	Avian_orthoavulavirus_1_isolate_363-Safsufa	2014	Israel	Class II	XXI	XXI.1.1
1311	OR364818.1	Avian_orthoavulavirus_1_isolate_375-2014	2014	Israel	Class II	VI	VI.2.1.2

1312	OR367435.1	UNVERIFIED:_Avian_orthoavulavirus_1_isolate_AoAV-1/UVAS-Pak/Pigeon/2011	2011	Pakistan	Class II	XX	XX
1313	OR367442.1	UNVERIFIED:_Avian_orthoavulavirus_1_isolate_AoAV-1/UVAS-Pak/Pigeon/2016	2016	Pakistan	Class II	XX	XX
1314	OR542540.1	Pigeon_paramyxovirus_1_strain_Pigeon/Hubei/187/2022	2022	China	Class II	VI	VI.2.1.1.2.2
1315	OR542541.1	Pigeon_paramyxovirus_1_strain_Pigeon/Hubei/188/2022	2022	China	Class II	VI	VI.2.1.1.2.2
1316	OR542542.1	Pigeon_paramyxovirus_1_strain_Pigeon/Hubei/190/2022	2022	China	Class II	VI	VI.2.1.1.2.2
1317	OR542543.1	Pigeon_paramyxovirus_1_strain_Pigeon/Zhejiang/1-3/2022	2022	China	Class II	VI	VI.2.1.1.2.2
1318	OR542544.1	Pigeon_paramyxovirus_1_strain_Pigeon/Zhejiang/3-2/2022	2022	China	Class II	VI	VI.2.1.1.2.2
1319	OR542545.1	Pigeon_paramyxovirus_1_strain_Pigeon/Zhejiang/5-2/2022	2022	China	Class II	VI	VI.2.1.1.2.2
1320	OR677829.1	Avian_orthoavulavirus_1_isolate_SA/Pigeon/F1/2022	2022	Saudi Arabia	Class II	I	I.1.1
1321	OR677830.1	Avian_orthoavulavirus_1_isolate_SA/Pigeon/F3/2021	2021	Saudi Arabia	Class II	I	I.1.1
1322	OR677831.1	Avian_orthoavulavirus_1_isolate_SA/Pigeon/F4/2022	2022	Saudi Arabia	Class II	I	I.1.1
1323	OR677832.1	Avian_orthoavulavirus_1_isolate_SA/Pigeon/F2/2021	2021	Saudi Arabia	Class II	VI	VI.2.1.2
1324	OR677833.1	Avian_orthoavulavirus_1_isolate_SA/Pigeon/I2/2021	2021	Saudi Arabia	Class II	VI	VI.2.1.2
1325	OR677834.1	Avian_orthoavulavirus_1_isolate_SA/Pigeon/I3/2021	2021	Saudi Arabia	Class II	VI	VI.2.1.2
1326	OR860421	Pigeon/China/HB2306/2023	2023	China	Class II	VI	VI.2.1.1.2.2
1327	OR860422	Pigeon/China/HB2307/2023	2023	China	Class II	VI	VI.2.1.1.2.2
1328	OR860423	Pigeon/China/HB2308/2023	2023	China	Class II	VI	VI.2.1.1.2.2
1329	OR860424	Pigeon/China/HB2309/2023	2023	China	Class II	VI	VI.2.1.1.2.2
